# Natural products as sources of acetylcholinesterase inhibitors: Synthesis, biological activities, and molecular docking studies of osthole-based ester derivatives

**DOI:** 10.3389/fpls.2022.1054650

**Published:** 2022-11-18

**Authors:** Xiang Yu, Yan Zhang, Minjie Zhang, Yafang Chen, Wude Yang

**Affiliations:** ^1^ College of Pharmacy, Guizhou University of Traditional Chinese Medicne, Guiyang, China; ^2^ Guizhou Joint Laboratory for International Cooperation in Ethnic Medicine, Guizhou University of Traditional Chinese Medicne, Guiyang, China

**Keywords:** osthole, structural modification, acetylcholinesterase inhibitor, molecular docking, network pharmacology

## Abstract

Osthole is a natural coumarin compound which isolated from *Cnidium monnieri (L.)* Cusson, has extensive pharmacological activities and could be used as a leading compound for drug research and development. In a continuous effort to develop new acetylcholinesterase inhibitors from natural products, eighteen osthole esters were designed, synthesized, and confirmed by ^1^H NMR, ^13^C NMR and HRMS. The anti-AChE activity of These derivatives was measured at a concentration of 1.0 mol/mL *in vitro* by Ellman's method, and the result showed that 4m and 4o had moderate inhibitory activities with 68.8% and 62.6%, respectively. Molecular docking study results further revealed AChE interacted optimally with docking poses 4m and 4o. Network pharmacology also predicted that compound 4m could be involved in Ras signaling pathway, which made it a potential therapeutic target of AD.

## Introduction

Alzheimer’s disease (AD) is a neurodegenerative brain disorder characterized by memory loss and cognitive impairments, which has affected 50 million people worldwide, with numbers projected to reach 135.5 million by 2050 (Lane et al., 2018; Bertram and Tanz, 2020). The neuropathological hallmarks of the disease are the presence of numerous senile amyloid β-peptide (Aβ) plaques, neurofibrillary tangles (NFT), synaptic loss and cholinergic neuron degeneration, or atrophy in the basal forebrain (Roberson and Harrell, 1997). With the loss of basal forebrain cholinergic cells, acetylcholine (ACh) decreases sharply, which is thought to contribute to cognitive impairments associated with AD (Bartus et al., 1982; Dunnett and Fobiger, 1993). Currently, one of the most common AD treatments is to suppress acetylcholinesterase activity in the brain in order to improve cognitive function.

Acetylcholinesterase (AChE), which is crucial for nerve conduction, primarily degrades acetylcholine (Nazir et al., 2018; Penumala et al., 2018). Acetylcholine is rapidly hydrolyzed by it at cholinergic synapses to terminate nerve impulse transmission (Silman and Sussman, 2005). X-ray crystallography studies revealed that there were two binding sites, the catalytic active site (CAS) at the bottom and the peripheral anionic site (PAS) near the entrance of the gorge (Bourne et al., 2003; Baharloo et al., 2015). Some studies have revealed that AChE could also play a key role in accelerating Aβ plaque deposition (Inestrosa et al., 1996; Hardy and Selkoe, 2002). AChE was also reported to interact with Aβ and promote amyloid fibril formation *via* a pool of amino acids located in proximity of the PAS (De Ferrari et al., 2001). Therefore, many pharmaceuticals have been developed for AD symptomatic treatment, such as rivastigmine, galantamine, tacrine, and donepezil (Schneider, 2000; Aranda-Abreu et al., 2011). However, these AChE inhibitors are commonly used in patients with Alzheimer’s to improve their cognitive function. These medications can cause nausea, diarrhea, anorexia, and abdominal pain (Shah and Reichman, 2006; Costantino et al., 2008; Jia et al., 2013). Accordingly, it is being attempted to develop natural AChE inhibitors that can replace the existing AChE inhibitors (Hansen et al., 2008).

**Figure 1:**
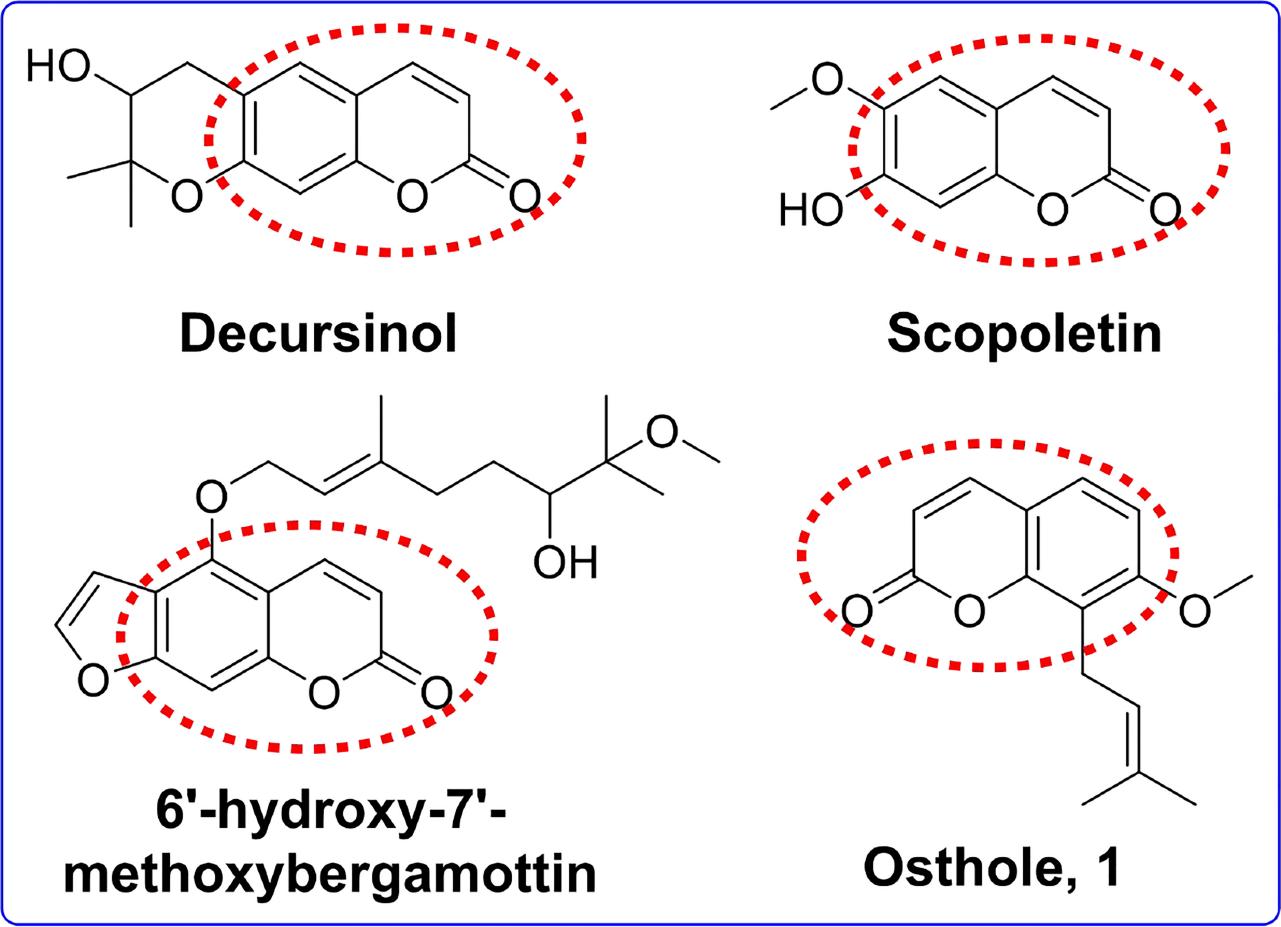
The chemical structures of osthole and several potent acetylcholinesterase inhibitors with coumarin moiety.

Coumarins are a group of plant natural products obtained from the phenylpropanoid pathway, found in a wide range of plant species in nature, and are classified into four main groups (Hawryl et al., 2000; Lin et al., 2013). The biological activities of coumarins have been found to include anticancer, anti-inflammatory, antiviral, antimicrobial, antiasthmatic, antioxidant, antinociceptive, antidiabetic, and antidepressant effects (Nawrot-Modranka et al., 2006; Fylaktakidou et al., 2008; Smyth et al., 2009; Hassan et al., 2016). Some studies also indicated that coumarins exhibited potent AChE inhibitory activity. For instance, decursinol (**Figure 1**) and scopoletin (**Figure 1**) were reported to exhibit the most potent AChE inhibition (Kang et al., 2001; Rollinger et al., 2004). Youkwan et al. found that 6′-hydroxy-7′-methoxybergamottin (**Figure 1**) exhibited anti-AChE activity with IC_50_ values of 11.2 μM (Youkwan et al., 2010). Thus, scientists increasingly seek to explore the coumarin template for synthesizing novel AChE inhibitors.

Osthole (1, **Figure 1**), mainly isolated from *Cnidium monnieri* (L.) Cusson and other forest plant species, is a natural coumarin compound and has extensive pharmacological features, such as anticancer, anti-inflammatory, and neuroprotective activities (Liu et al., 2015; Zhang et al., 2015; Fan et al., 2019; Tang et al., 2020; Bae et al., 2021; Sun et al., 2021). Researchers have found that osthole suppressed inflammation and apoptosis in mouse models of stab wound injuries, thus reducing secondary brain damage, enhancing the memory and learning functions in mechanical brain injury mice, and increasing the number of neurons in the affected brain regions (Xia et al., 2015; Yan et al., 2018). These all show that osthole is a promising skeleton for developing anti-Alzheimer’s drugs. However, few reports are related to the anti-AChE activity of osthole. Following the abovementioned interesting results, and as part of our ongoing search for new potential natural-product-based AChE inhibitors (Yu et al., 2021), in this paper, as part of our study, we prepared a series of osthole-based ester derivatives, measured the anti-AChE activity by Ellman’s method, and explored possible mechanisms of action using molecular modeling. On the other hand, we also used network pharmacology to screen other potential targets of derivatives in AD and molecular mechanisms.

## Results and discussion

### Chemical synthesis

**Figure 2:**
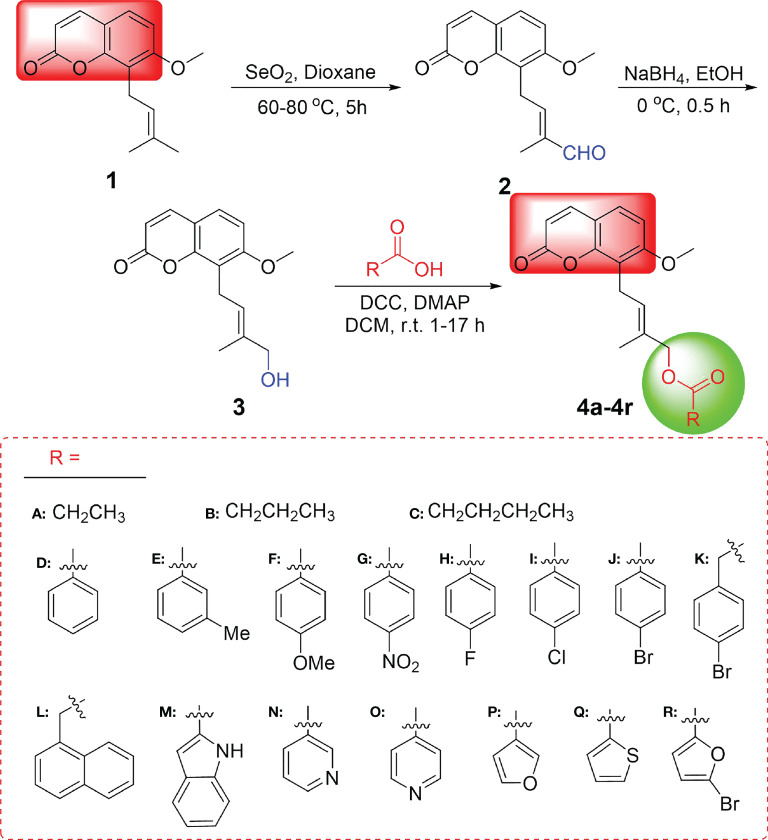
Synthetic route for target compounds 4a–4r.

The synthesis of osthole-based ester derivatives was performed as illustrated in **Figure 2** *via* our previously reported methods. Firstly, oxidation of osthol (1) with SeO_2_ obtained 3′-formaldehydylosthole (2) in 46% (Yu et al., 2016). After being reduced by NaBH_4_ at 0° C, compound 2 yielded 4′-hydroxyosthole (3) (Yu et al., 2015). Finally, a series of osthole esters derivatives (4a–4r) were converted in 50%–69% yields by esterification of intermediate 3 with various carboxylic acids using dicyclohexylcarbodiimide (DCC) and 4-dimethylaminopyridine (DMAP) (Yu et al., 2015). They were characterized using ^1^H NMR, ^13^C NMR, and HRMS, and exemplary data are listed in **Supplementary Data**.

### Anti-AChE activity *in vitro*

A preliminary bioassay of these derivatives’ activities inhibiting AChE was performed *in vitro* using the Ellman method at 0.01, 0.1, and 1 μmol/ml, respectively. As shown in **Table 1**, most of the target compounds had better inhibitory activities against AChE than raw material **1** at 1 μmol/ml; especially 4e, 4m, and 4o had significant inhibitory effects with inhibitory rates exceeding 50% but did not surpass tacrine. Among them, the most active of these was 4m, which showed an inhibitory rate of 68.8%, followed by 4o, which showed a rate of 62.6%. On the other hand, a structure–activity relationship for these osthole-based esters was also examined. Compounds 4m and 4o, which had superior inhibitory effects on AChE, contained aromatic heterocycles and suggested that the anti-AChE activity might be enhanced by the introduction of aromatic heterocycles compared with compound **1**. Our previous research also showed that the introduction of heterocycles in coumarins could improve biological activity (Yu et al., 2021). On the contrary, target compounds with alkyl groups showed lower inhibitory activity at 1 μmol/ml; it showed that alkyl groups did not significantly increase activity when introduced (e.g., 19.4% for 4a, 22.3% for 4b>, 20.2% for 4a).

**Table 1:** The inhibitory activity of titled compounds (**4a–4r**) against AChE *in vitro*.

Compound	Inhibition rate^a^ (%)		
	0.01 μmol/ml	0.1 μmol/ml	1 μmol/ml
**4a**	5.9 ± 2.6	6.2 ± 3.0	19.4 ± 2.3hij^c^
**4b**	8.4 ± 4.9	16.9 ± 3.0	22.3 ± 3.9ghi
**4c**	8.7 ± 1.2	18.4 ± 3.4	20.2 ± 2.8hij
**4d**	9.1 ± 3.3	17.0 ± 4.9	33.0 ± 2.8de
**4e**	12.2 ± 3.4	32.2± 3.1	56.7 ± 3.3b
**4f**	3.7 ± 3.2	10.5 ± 3.2	28.7 ± 2.1efg
**4g**	1.6 ± 3.6	9.4 ± 0.3	28.5 ± 4.2efg
**4h**	1.8 ± 2.4	3.9 ± 5.5	13.8 ± 5.4j
**4i**	2.3 ± 2.1	18.5 ± 5.2	38.6 ± 4.3d
**4j**	3.8 ± 2.4	13.9 ± 2.6	28.2 ± 2.3efg
**4k**	3.6 ± 3.5	8.4 ± 2.9	23.9 ± 5.5fghi
**4l**	1.2 ± 1.5	2.5 ± 3.5	18.8 ± 1.9ij
**4m**	21.0 ± 2.4	43.0 ± 2.8	68.8 ± 2.6a
**4n**	11.2 ± 2.1	21.4 ± 2.0	48.7 ± 0.5c
**4o**	18.4 ± 3.6	32.6 ± 1.6	62.6 ± 1.6ab
**4p**	3.5 ± 2.4	14.5 ± 2.2	26.3 ± 3.0efgh
**4q**	1.5 ± 2.3	5.6 ± 1.7	23.4 ± 2.0fghi
**4r**	2.3 ± 2.1	8.0 ± 0.3	30.6 ± 2.0ef
**1**	2.3 ± 2.6	3.6 ± 2.4	16.7 ± 3.2ij
**Tacrine** ^b^	22.8 ± 2.6	53.5 ± 1.7	67.6 ± 2.6

a Values were the mean ± SD of three replicates.

b Tacrine was controlled and tested in 0.01, 0.01, and 1 μmol/l.

c Multiple- range test using Duncan’s test (p < 0.05). The same letters denote treatments that are not significantly different from each other.

Meanwhile, compared with the inhibitory rates of 4m and 4o at different concentrations, we found that the inhibitory rates increased in a linear manner with an increase in compound concentration. It indicated that there was a positive correlation between inhibitory activities and concentration.

### Molecular docking results

**Figure 3:**
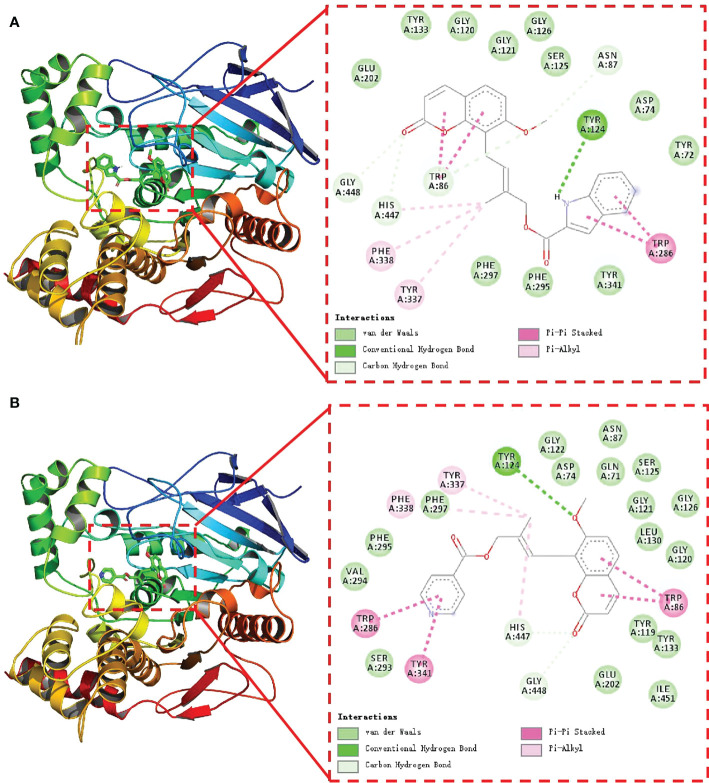
Docking pose of compound 4m **(A)** and 4o **(B)** inside AChE (the green dotted lines show the hydrogen bonds, the pink full lines show the π–π interactions.).

**Figure 4:**
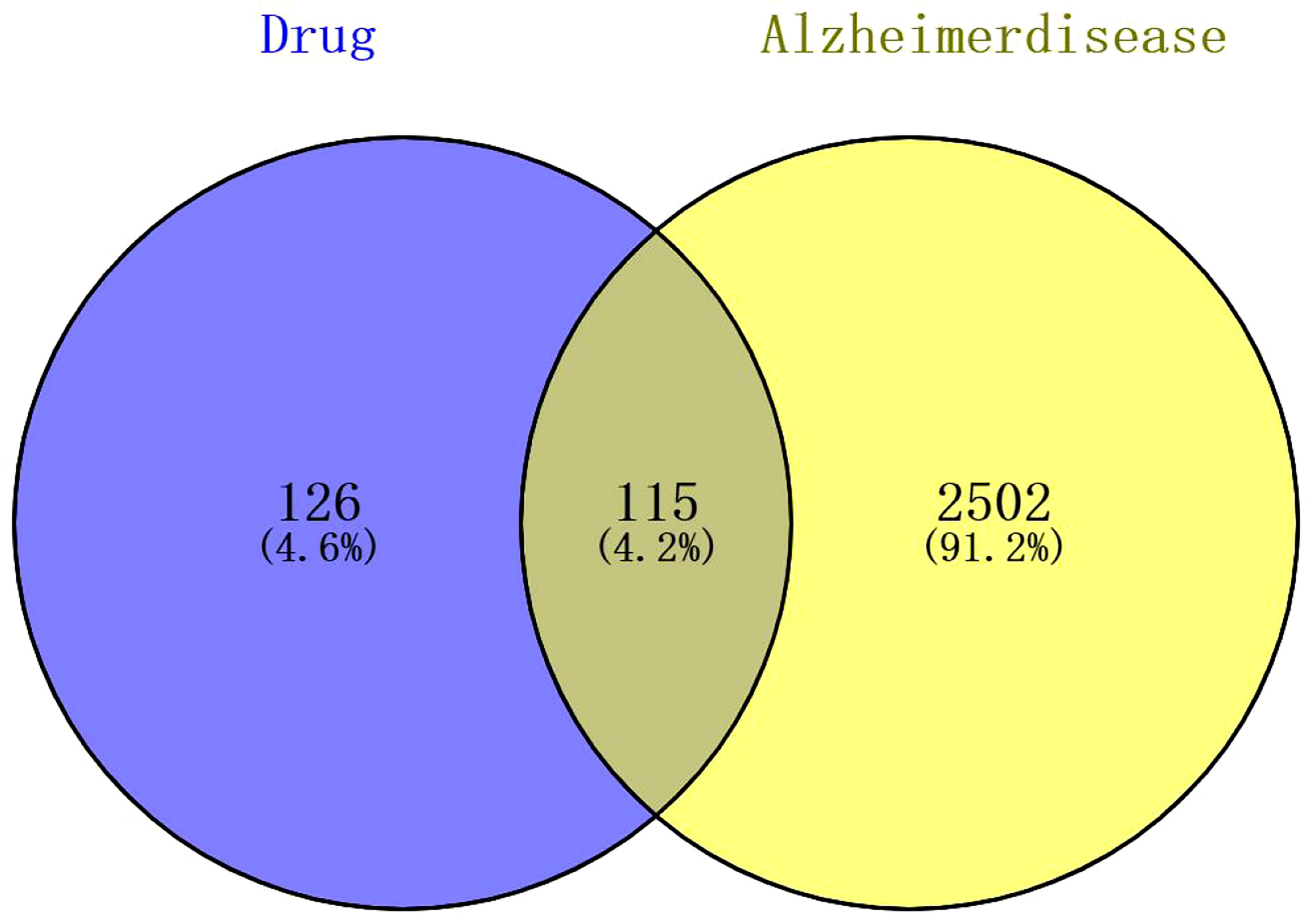
The Venn diagram of compound 4m and AD targets.

In our previous study, we found that coumarin could conjugate with the amino acid residues of acetylcholinesterase, thus showing a certain inhibitory activity (Yu et al., 2021). To explore the possible inhibition mechanism of the potent compound, molecular modeling studies were also performed on compounds 4m and 4o in the active site of AChE. AChE’s 3D structure was selected for docking studies from the RCSB database (PDB code: 3DHP). In this study, compounds 4m, 4o and AChE binding energies were -11.5 and -10.0 kcal/mol, respectively, which indicated that 4m and 4o had better binding activity with core targets. As illustrated in **Figure 3**, AChE’s active channel substrate (combinations 4m in **Figure 3A** and 4o in **Figure 3B**) contains the coumarin portion, whereas its channel entrance contains the aromatic heterocycles. In parallel, the conjugated aromatic ring of coumarin made a π–π interaction with the Trp86 residue to locate the coumarin core in AChE’s active site. In addition, the aromatic heterocycles were bonded to the Trp286 residue of the channel entrance *via* the π–π interaction. Acetylcholine could not enter the catalytic center of AChE since 4m and 4o occupied the catalytic site. In combination with the biological assay results, this molecular docking result suggested that compounds 4m and 4o might inhibit AChE.

### Analysis of network pharmacology

By searching the public databases (PubChem, SwissTargetPrediction, PharmMapper, SEA, GEO, GeneCard, OMIM), confining the result to “Homo sapiens,” 241 targets related to compound 4m and 2617 AD targets were collected, respectively. By using R software, the intersection of 4m targets and AD disease targets was calculated, and a Venn diagram was drawn to obtain 115 intersection targets (**Figure 4**). After that, PPI networks were constructed using target proteins and their corresponding ingredients in the STRING database (http://string-db.org/), and high confidence of protein interaction data with a score >0.7 was selected. By removing free proteins that do not interact, 4m and AD share 98 proteins.

**Figure 5:**
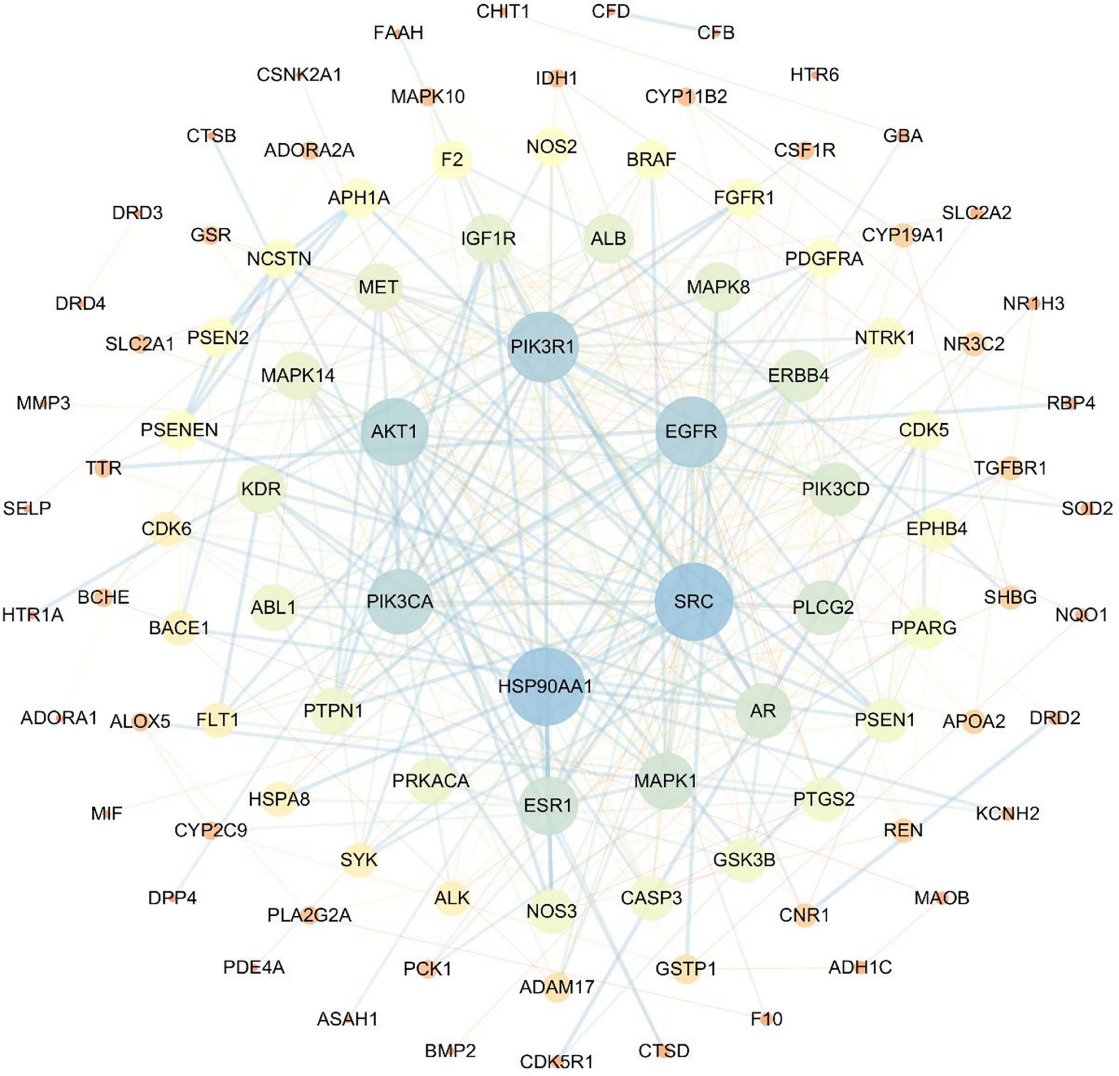
Topological network schematic of proteins targeted by 4m and associated with AD.

Based on the protein–protein interaction networks, 98 proteins and 330 interactions were identified as potential interactions between compound 4m and AD (**Figure 5**).

**Figure 6:**
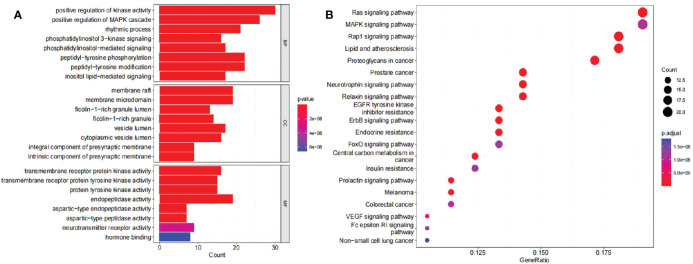
Analysis of potential targets of 4m for AD treatment based on GO and KEGG enrichment. **(A)** GO enrichment analysis identified genes involved in GO-BP analysis, GO-CC analysis, GO-MF analysis; **(B)** KEGG pathway analyses from bioinformatics data for the molecular signal pathway).

GO enrichment analysis and KEGG pathway enrichment analysis were performed to elucidate the functions and enriched pathways of compound 4m’s potential anti-AD genes. As a result of the GO analysis, 2,151 statistically significant terms were obtained with 1,896 of biological processes (BP), 96 of cellular components (CC), and 159 of molecular functions (MF), according to p < 0.05. As shown in **Figure 6A**, the bar plot diagram displayed top eight significant enrichment terms of BP, CC, and MF with the highest gene counts, and redder dots indicated a lower q value and greater GO term enrichment. The results showed that compound 4m’s targets in treating AD were mostly enriched by positive regulation of kinase activity, positive regulation of MAPK cascade, rhythmic process, and other biological processes; in membrane raft, membrane microdomain, ficolin-1-rich granule lumen, and other cellular components; and in transmembrane receptor protein kinase activity, transmembrane receptor protein tyrosine kinase activity, protein tyrosine kinase activity, and other molecular functions. In order to explore the functions and signaling pathways of 4m’s identified anti-AD targets, KEGG pathways were applied. As a result, 151 signaling pathways related to 4m-AD were statistically significant, including Ras signaling pathway, MAPK signaling pathway, and Pap1 signaling pathway. An illustrated bubble diagram displayed the top 20 pathways showing significant enrichment potential with the highest number of genes (**Figure 6B** **)**.

**Figure 7:**
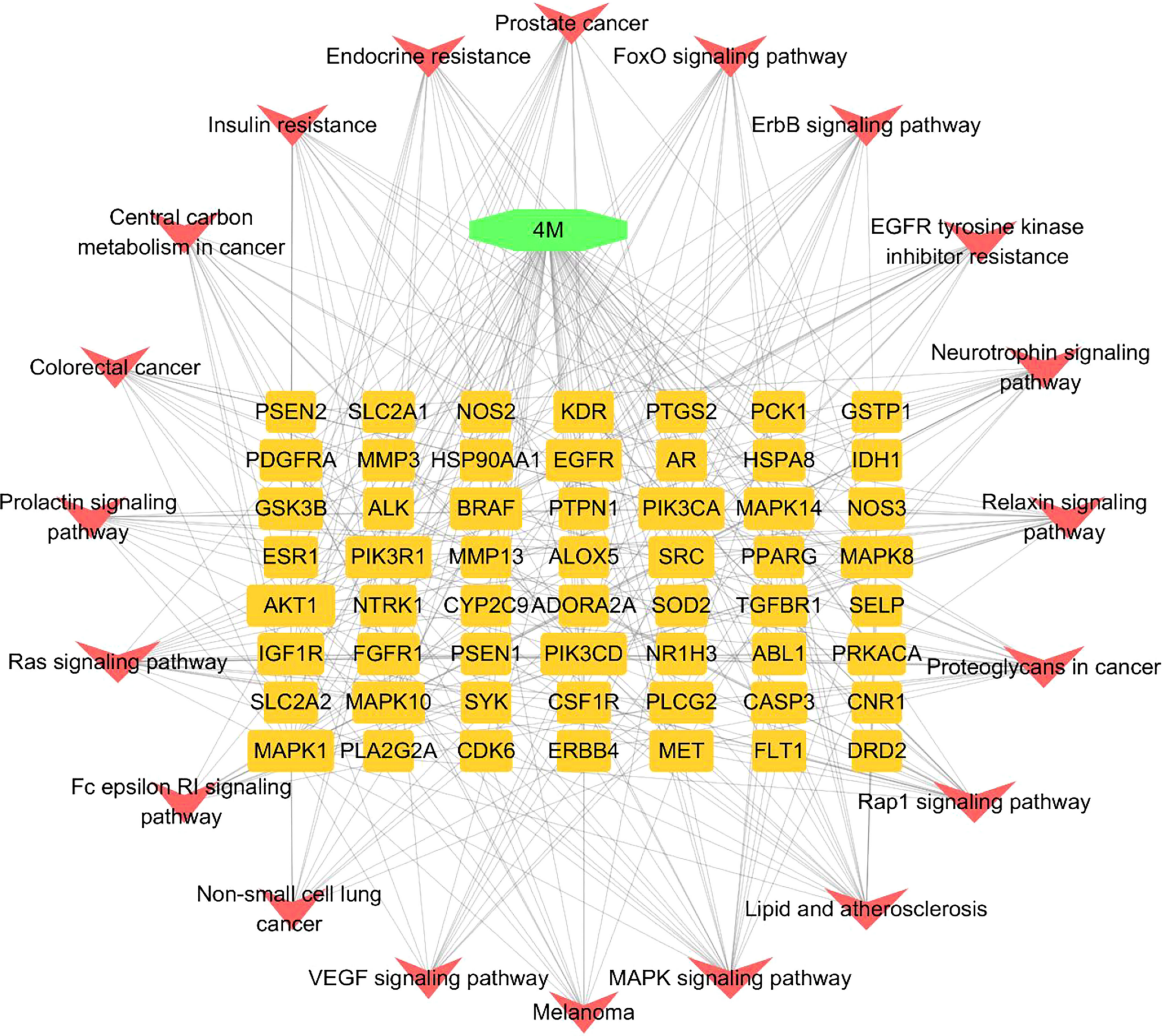
Constituent–target–pathway network of top 20 pathways. (The nodes in green stands for compound 4m. Each yellow oblong on the inner circle stands targets. Each red V node stands for each pathway.).

**Figure 8:**
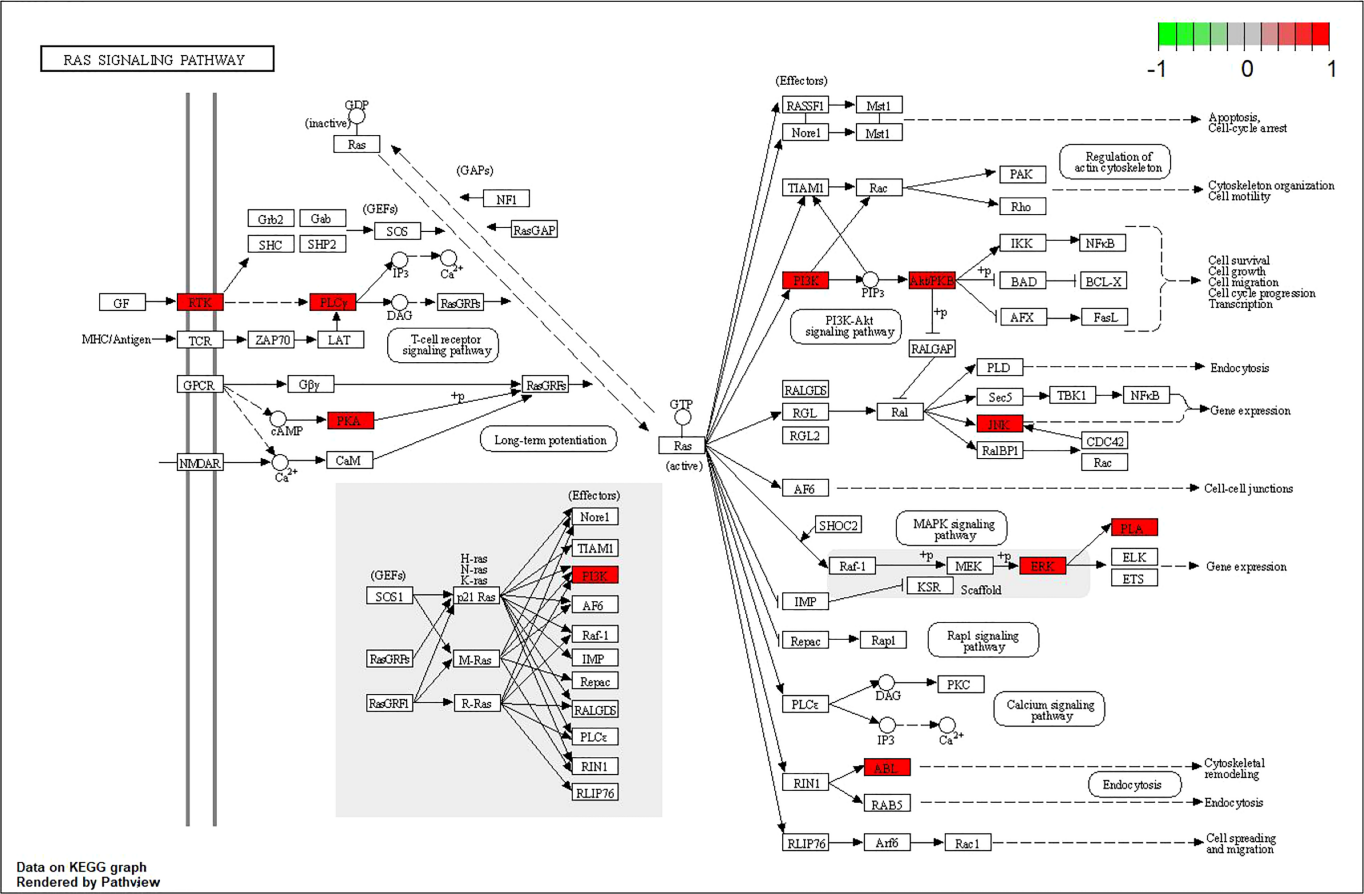
Ras signaling pathway map (nodes in red represent 4m-AD-related genes).

In addition, a constituent–target–pathway network containing 77 nodes and 348 edges was constructed to examine the interrelationships between ingredients, targets, and the top 20 pathways (**Figure 7**). Among the top three pathway counts, we found Ras signaling pathway, MAPK signaling pathway, and Pap1 signaling pathway, which may be responsible for the anti-AD effect of 4m. The Ras signaling pathway, in which it was proved that Ras farnesylation was significantly higher than in the elderly with non-cognitive disorders in the brain of AD patients (Dineley et al., 2001), contributed to the most genes and might be the most important 4m-AD pathway (**Figure 8**).

**Figure 9:**
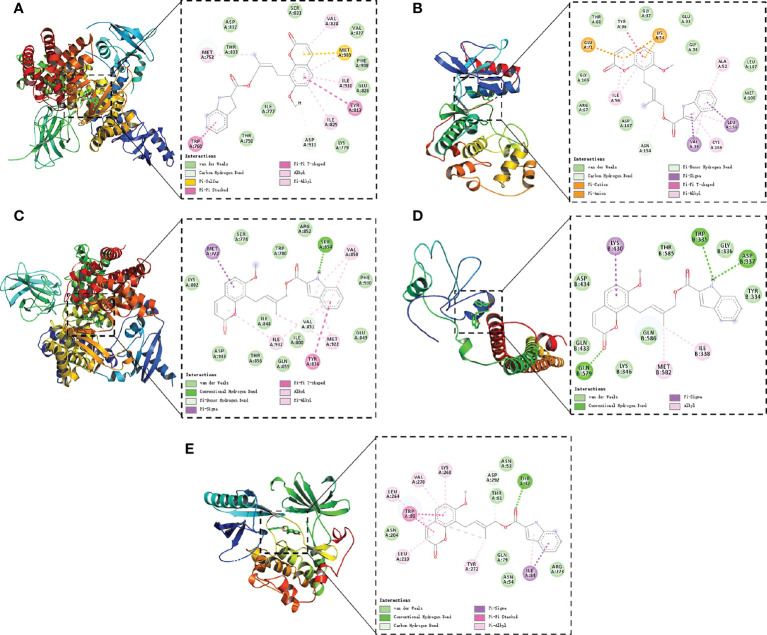
Molecular models of 4m binding to AKT1 **(A)**, PIK3CD **(B)**, PIK3CA **(C)**, PIK3R1 **(D)**, and MAPK1 **(E)**. The green dotted lines show the hydrogen bonds; the pink full lines show the π–π interactions.).

Finally, molecular docking was conducted between compound 4m and the five key targets (AKT1, PIK3CD, PIK3CA, PIK3R1MAPK1). As shown in **Figure 9**, it was found that compound 4m bound to target proteins with binding energies lower than -7 kcal/mol, suggesting that 4m inhibited the docking pocket from binding to the target receptor, making it an effective treatment for Alzheimer’s disease.

## Conclusion

In the present study, we designed, synthesized, and evaluated 18 derivatives of the osthole ester derivative for their *in vitro* inhibitory activity against AChE. The derivatives 4m and 4o showed moderate inhibitory activities, which were positively correlated with concentrations. Molecular docking results further revealed compounds 4m and 4o could bind to AChE through hydrogen bonds and hydrophobic contact. Network pharmacology also predicted that compound 4m could be involved in the Ras signaling pathway, which made it a potential therapeutic target of AD.

## Materials and methods

Materials and Methods see **Supplementary Data**.

## Data availability statement

The original contributions presented in the study are included in the article/**Supplementary Material**. Further inquiries can be directed to the corresponding authors.

## Author contributions

XY, YC, and WY conceived and designed the experiments. XY performed the synthetic experiments, and wrote the manuscript. MZ performed the bioassays. YZ analyzed the data. YZ, YC, and WY revised the manuscript. All authors contributed to the article and approved the submitted version.

## Funding

Our work was supported by the Natural Science Foundation of Guizhou Province [QKH-J(2020)1Y070], the Natural Science Foundation of China (No. 82260833), and the Youth Talent Development Project of Education Department of Guizhou Province [2018]209.

## Conflict of interest

The authors declare that the research was conducted in the absence of any commercial or financial relationships that could be construed as a potential conflict of interest.

## Publisher’s note

All claims expressed in this article are solely those of the authors and do not necessarily represent those of their affiliated organizations, or those of the publisher, the editors and the reviewers. Any product that may be evaluated in this article, or claim that may be made by its manufacturer, is not guaranteed or endorsed by the publisher.
