# Supplementary material for: Natural products as sources of acetylcholinesterase inhibitors: Synthesis, biological activities, and molecular docking studies of osthole-based ester derivatives
Source: Front Plant Sci. 2022 Nov 18;13:1054650. doi: 10.3389/fpls.2022.1054650 (PMC9716088; doi:10.3389/fpls.2022.1054650)
Supplement: Supplementary file 1 [file DataSheet_1.doc]

**Materials and Methods**

**Materials and Chemicals**

All reagents and solvents required for the compounds were of reagent grade or purified according to standard methods before use. Analytical thin-layer chromatography (TLC) and preparative thin-layer chromatography (PTLC) were performed with silica gel plates using silica gel 60 GF_254_ (Qingdao Haiyang Chemical Co., Ltd.). Melting points were determined on a XT-4 digital melting point apparatus (Beijing Tech Instrument Co., Ltd., Beijing, China) and were uncorrected. Nuclear magnetic resonance spectra were recorded on a Bruker Avance DEO 400 or 600 MHz instrument (Bruker, Bremerhaven, Germany) in CDCl_3_ or DMSO-*d*_6_ using TMS (tetramethylsilane) as the internal standard. The chemical shifts for the NMR spectra were reported in δ ppm. Peak multiplicities were expressed as singlet (s), doublet (d), triplet (t), and multiplet (m). Mass spectrometry (MS) was carried out with a Waters XEVO TQ-D instrument (Waters, Massachusetts, USA). High-resoiution mass spectrometry (HRMS) was carried out with a Xevo G2-SQTOF instrument (Waters, Massachusetts, USA).

**Synthetic Procedures**

**Synthesis of intermediate 2.** To a solution of osthole (**1**, 1 mmol, 244.3 mg) in dioxane (10 mL) at 60 ^o^C was added in SeO_2_ (1.1 mmol, 122.1 mg), and then the reaction temperature rapidly increased 80 ^o^C. When the reaction mixture was stirred for 5 h (checked by TLC) and cooled to room temperature, the precipitates were filtered off, washed thoroughly with CH_2_Cl_2_ (20 mL), and the combined organic phase dried over anhydrous Na_2_SO_4_ and concentrated. The residue was separated by preparative thin-layer chromatography (PTLC) to abtain pure compound **2** in 46% yield, White solid, mp 116-117 ^o^C; ^1^H NMR (400 MHz, DMSO-*d*_6_) *δ*: 9.36 (s, 1H, CHO), 7.99 (d, *J* = 9.6 Hz, 1H, 4-H), 7.64 (d, *J* = 8.4 Hz, 1H, 5-H), 7.11 (d, *J* = 8.8 Hz, 1H, 6-H), 6.57 (t, *J* = 7.2 Hz, 1H, 2’-H), 6.30 (d, *J* = 9.6 Hz, 1H, 3-H, ), 3.93 (s, 3H, OCH_3_), 3.77 (d, *J* = 7.6 Hz, 2H, 1’-H), 1.85 (s, 3H, 5’-H); MS (ESI, *m/z*): 281.1 ([M + Na]^+^, 100).

**Synthesis of intermediate 3.** To a stirred solution of compound **2** (1 mmol) in ethanol (10 mL), sodium borohydride (1.2 mmol) in ethanol (2 mL) was added at 0 °C. After the reaction was complete checked by TLC after 0.5 h, it was quenched by 0.2 M aqueous HCl (5 mL). The reaction mixture was extracted with CH_2_Cl_2_ (10 mL×3), the combined organic layer was dried over anhydrous Na_2_SO_4_ and purified by PTLC to give compound **3** in 95% yield, white solid, mp 118-120 °C; ^1^H NMR (600 MHz, CDCl_3_) *δ*: 7.62 (d, *J* = 9.4 Hz, 1H, 4-H), 7.31 (d, *J* = 8.4 Hz, 1H, 5-H), 6.84 (d, *J* = 8.5 Hz, 1H, 6-H), 6.24 (d, *J* = 9.4 Hz, 1H, 3-H), 5.50 (t, *J* = 7.2 Hz, 1H, 2’-H), 3.98 (s, 2H, 4’-H), 3.92 (s, 3H, OCH_3_), 3.60 (d, *J* = 7.2 Hz, 2H, 1’-H), 1.89 (s, 3H, 5’-H); MS (ESI, *m/z*): 283.1 ([M+Na]^+^, 100).

**General synthetic procedure for target compounds 4a-4r.** A mixture of compound **3** (0.5 mmol), carboxylic acids RCO_2_H (0.6 mmol), N,N’-dicyclohexylcarbodiimide (DCC, 0.7 mmol), and 4-dimethylaminopyridine (DMAP, 0.1 mmol) in dry CH_2_Cl_2_ (10 mL) was stirred at room temperature. When the reaction was complete according to TLC analysis, the mixture was diluted by CH_2_Cl_2_ (20 mL), washed by water (15 mL), aqueous HCl (0.1 M, 15 mL), 5% aqueous Na_2_CO_3_ (15 mL) and brine (15 mL). Finally, the organic phase was dried over anhydrous Na_2_SO_4_, concentrated under reduced pressure, and the residue was puried by PTLC to give the pure target products **4a-4r** in 64–97% yields.

**4a**: Yield = 69%, white solid, mp 92-94 °C; ^1^H NMR (600 MHz, CDCl_3_) δ: 7.62 (d, *J* = 9.4 Hz, 1H, 4-H), 7.31 (d, *J* = 8.6 Hz, 1H, 5-H), 6.84 (d, *J* = 8.5 Hz, 1H, 6-H), 6.24 (d, *J* = 9.4 Hz, 1H, 3-H), 5.55 (t, *J* = 7.4 Hz, 1H, 2’-H), 4.44 (s, 2H, 4’-H), 3.92 (s, 3H, OCH_3_), 3.60 (d, *J* = 7.4 Hz, 2H, 1’-H), 2.33 (q, *J* = 7.6 Hz, 2H, CH_2_CH_3_), 1.88 (s, 3H, 5’-H), 1.13 (t, *J* = 7.6 Hz, 3H, CH_2_CH_3_); ^13^C NMR (101 MHz, CDCl_3_) δ: 174.4, 161.2, 160.2, 152.8, 143.7, 131.2, 126.6, 125.8, 116.6, 113.0, 112.9, 107.3, 69.9, 56.0, 27.6, 21.6, 14.1, 9.1; HRESIMS m/z 339.1212 [M+Na]^+^ (calcd for C_18_H_20_O_5_Na, 339.1202).

**4b**: Yield = 56%, white solid, mp 88-90 °C; ^1^H NMR (600 MHz, CDCl_3_) δ: 7.62 (d, *J* = 9.4 Hz, 1H, 4-H), 7.31 (d, *J* = 8.6 Hz, 1H, 5-H), 6.84 (d, *J* = 8.5 Hz, 1H, 6-H), 6.24 (d, *J* = 9.4 Hz, 1H, 3-H), 5.55 (t, *J* = 6.4 Hz, 1H, 2’-H), 4.44 (s, 2H, 4’-H), 3.92 (s, 3H, OCH_3_), 3.60 (d, *J* = 7.3 Hz, 2H, 1’-H), 2.29 (t, *J* = 7.4 Hz, 2H, CH_2_CH_2_CH_3_), 1.88 (s, 3H, 5’-H), 1.64 (h, *J* = 7.4 Hz, 2H, CH_2_CH_2_CH_3_), 0.93 (t, *J* = 7.4 Hz, 3H, CH_2_CH_2_CH_3_); ^13^C NMR (101 MHz, CDCl_3_) δ: 173.6, 161.2, 160.2, 152.8, 143.7, 131.2, 126.5, 125.8, 116.7, 113.0, 112.9, 107.3, 69.8, 56.0, 36.2, 21.6, 18.4, 14.1, 13.6; HRESIMS m/z 353.1361 [M+Na]^+^ (calcd for C_19_H_22_O_5_Na, 353.1359).

**4c**: Yield = 54%, white solid, mp 84-86 °C; ^1^H NMR (600 MHz, CDCl_3_) δ: 7.62 (d, *J* = 9.5 Hz, 1H, 4-H), 7.31 (d, *J* = 8.6 Hz, 1H, 5-H), 6.84 (d, *J* = 8.6 Hz, 1H, 6-H), 6.24 (d, *J* = 9.4 Hz, 1H, 3-H), 5.55 (t, *J* = 8.1 Hz, 1H, 2’-H), 4.44 (s, 2H, 4’-H), 3.92 (s, 3H, OCH_3_), 3.60 (d, *J* = 7.4 Hz, 2H, 1’-H), 2.31 (t, *J* = 7.6 Hz, 2H, CH_2_CH_2_CH_2_CH_3_), 1.88 (s, 3H, 5’-H), 1.63 – 1.57 (m, 2H, CH_2_CH_2_CH_2_CH_3_), 1.33 (h, *J* = 7.4 Hz, 2H, CH_2_CH_2_CH_2_CH_3_), 0.90 (t, *J* = 7.4 Hz, 3H, CH_2_CH_2_CH_2_CH_3_); ^13^C NMR (101 MHz, CDCl_3_) δ: 173.9, 161.2, 160.2, 152.8, 143.7, 131.2, 126.6, 125.8, 116.6, 113.0, 112.9, 107.3, 69.8, 56.0, 34.0, 27.0, 22.2, 21.6, 14.1, 13.7; HRESIMS m/z 367.1512 [M+Na]^+^ (calcd for C_20_H_24_O_5_Na, 367.1515).

**4d**: Yield = 61%, white solid, mp 97-99 °C; ^1^H NMR (600 MHz, CDCl_3_) δ: 8.04 (d, *J* = 7.0 Hz, 2H, ArH), 7.62 (d, *J* = 9.4 Hz, 1H, 4-H), 7.54 (t, *J* = 7.4 Hz, 1H, ArH), 7.43 (t, *J* = 7.8 Hz, 2H, ArH), 7.32 (d, *J* = 8.6 Hz, 1H, 5-H), 6.84 (d, *J* = 8.4 Hz, 1H, 6-H), 6.25 (d, *J* = 9.4 Hz, 1H, 3-H), 5.66 (t, *J* = 7.2 Hz, 1H, 2’-H), 4.69 (s, 2H, 4’-H), 3.92 (s, 3H, OCH_3_), 3.64 (d, *J* = 7.3 Hz, 2H, 1’-H), 1.97 (s, 3H, 5’-H); ^13^C NMR (101 MHz, CDCl_3_) δ: 166.4, 161.2, 160.2, 152.8, 143.7, 132.8 131.1, 130.4, 129.6×2, 128.3×2, 126.6, 126.2, 116.6, 113.0, 113.0, 107.3, 70.6, 56.0, 21.6, 14.2; HRESIMS m/z 387.1195 [M+Na]^+^ (calcd for C_22_H_20_O_5_Na, 387.1202).

**4e**: Yield = 54%, white solid, mp 108-110 °C; ^1^H NMR (600 MHz, CDCl_3_) δ: 7.90-7.80 (m, 2H, ArH), 7.62 (d, *J* = 9.5 Hz, 1H, 4-H), 7.40-7.29 (m, 3H, ArH, 5-H), 6.85 (d, *J* = 8.6 Hz, 1H, 6-H), 6.25 (d, *J* = 9.4 Hz, 1H, 3-H), 5.66 (t, *J* = 7.3 Hz, 1H, 2’-H), 4.68 (s, 2H, 4’-H), 3.92 (s, 3H, OCH_3_), 3.64 (d, *J* = 7.3 Hz, 2H, 1’-H), 2.39 (s, 3H, ArCH_3_), 1.97 (s, 3H, 5’-H); ^13^C NMR (101 MHz, CDCl_3_) δ: 166.6, 161.2, 160.3, 152.8, 143.7, 138.1, 133.6, 131.2, 130.3, 130.1, 128.2, 126.7, 126.6, 126.2, 116.8, 113.0, 113.0.107.3, 70.5, 56.0, 21.6, 21.3, 14.2; HRESIMS m/z 401.1367 [M+Na]^+^ (calcd for C_23_H_22_O_5_Na, 401.1359).

**4f**: Yield = 54%, white solid, mp 115-117 °C; ^1^H NMR (600 MHz, CDCl_3_) δ: 7.99 (d, *J* = 8.9 Hz, 2H, ArH), 7.62 (d, *J* = 9.4 Hz, 1H, 4-H), 7.32 (d, *J* = 8.5 Hz, 1H, 5-H), 6.91 (d, *J* = 8.9 Hz, 2H, ArH), 6.84 (d, *J* = 8.6 Hz, 1H, 6-H), 6.25 (d, *J* = 9.4 Hz, 1H, 3-H), 5.65 (t, *J* = 6.7 Hz, 1H, 2’-H), 4.66 (s, 2H, 4’-H), 3.92 (s, 3H, OCH_3_), 3.85 (s, 3H, OCH_3_), 3.64 (d, *J* = 7.4 Hz, 2H, 1’-H), 1.97 (s, 1H, 5’-H); ^13^C NMR (101 MHz, CDCl_3_) δ: 166.2, 163.2, 161.2, 160.3, 152.8, 143.7, 131.6×2, 131.3, 126.6, 125.9, 122.8, 116.7, 113.5×2, 113.0, 113.0, 107.3, 70.3, 56.0, 55.4, 21.6, 14.2; HRESIMS m/z 417.1306 [M+Na]^+^ (calcd for C_23_H_22_O_6_Na, 417.1308).

**4g**: Yield = 50%, white solid, mp 130-132 °C; ^1^H NMR (600 MHz, CDCl_3_) δ: 8.28 (d, *J* = 8.8 Hz, 2H, ArH), 8.21 (d, *J* = 8.8 Hz, 2H, ArH), 7.64 (d, *J* = 9.4 Hz, 1H, 4-H), 7.34 (d, *J* = 8.6 Hz, 1H, 5-H), 6.86 (d, *J* = 8.6 Hz, 1H, 6-H), 6.26 (d, *J* = 9.4 Hz, 1H, 3-H), 5.69 (t, *J* = 8.0 Hz, 1H, 2’-H), 4.73 (s, 2H, 4’-H), 3.94 (s, 3H, OCH_3_), 3.65 (d, *J* = 7.4 Hz, 2H, 1’-H), 1.99 (s, 3H, 5’-H); ^13^C NMR (101 MHz, CDCl_3_) δ: 164.5, 161.2, 160.2, 152.9, 150.4, 143.7, 135.8, 130.7×2, 130.5, 127.2, 126.7, 123.5×2, 116.4, 113.1, 113.0, 107.3, 71.5, 56.1, 21.7, 14.3; HRESIMS m/z 432.1056 [M+Na]^+^ (calcd for C_22_H_19_NO_7_Na, 432.1053).

**4h**: Yield = 63%, white solid, mp 138-140 °C; ^1^H NMR (600 MHz, CDCl_3_) δ: 8.05 (dd, *J* = 8.8, 5.5 Hz, 2H, ArH), 7.63 (d, *J* = 9.4 Hz, 1H, 4-H), 7.32 (d, *J* = 8.5 Hz, 1H, 5-H), 7.10 (t, *J* = 8.7 Hz, 2H, ArH), 6.85 (d, *J* = 8.6 Hz, 1H, 6-H), 6.25 (d, *J* = 9.4 Hz, 1H, 3-H), 5.66 (t, *J* = 6.7 Hz, 1H, 2’-H), 4.67 (s, 2H, 4’-H), 3.92 (s, 3H, OCH_3_), 3.64 (d, *J* = 7.3 Hz, 2H, 1’-H), 1.97 (s, 3H, 5’-H); ^13^C NMR (101 MHz, CDCl3) δ: 166.9, 165.5, 164.4, 161.2, 160.2, 152.8, 143.7, 132.2, 132.1, 131.0, 126.6, 126.4, 116.5, 115.5, 115.3, 113.1, 113.0, 107.3, 70.7, 56.0, 21.6, 14.2; HRESIMS m/z 405.1108 [M+Na]^+^ (calcd for C_22_H_19_FO_5_Na, 405.1108).

**4i**: Yield = 57%, white solid, mp 88-100 °C; ^1^H NMR (600 MHz, CDCl_3_) δ: 7.97 (d, *J* = 8.6 Hz, 2H ArH), 7.62 (d, *J* = 9.5 Hz, 1H, 4-H), 7.40 (d, *J* = 8.5 Hz, 2H, ArH), 7.32 (d, *J* = 8.5 Hz, 1H, 5-H), 6.85 (d, *J* = 8.6 Hz, 1H, 6-H), 6.25 (d, *J* = 9.4 Hz, 1H, 3-H), 5.66 (t, *J* = 7.2 Hz, 1H, 2’-H), 4.68 (s, 2H, 4’-H), 3.92 (s, 3H, OCH_3_), 3.64 (d, *J* = 7.3 Hz, 2H, 1’-H), 1.96 (s, 3H, 5’-H); ^13^C NMR (101 MHz, CDCl^3^) δ: 165.6, 161.1, 160.2, 152.9, 143.7, 139.2, 131.0×2, 130.9, 128.8, 128.6×2, 126.6, 126.5, 116.6, 113.1, 113.0, 107.3, 70.8, 56.0, 21.6, 14.2; HRESIMS m/z 421.0817 [M+Na]^+^ (calcd for C_22_H_19_ClO_5_Na, 421.0813).

**4j**: Yield = 54%, white solid, mp 170-172 °C; ^1^H NMR (600 MHz, CDCl_3_) δ: 7.89 (d, *J* = 8.6 Hz, 2H, ArH), 7.62 (d, *J* = 9.4 Hz, 1H, 4-H), 7.57 (d, *J* = 8.6 Hz, 2H, ArH), 7.32 (d, *J* = 8.5 Hz, 1H, 5-H), 6.85 (d, *J* = 8.6 Hz, 1H, 6-H), 6.25 (d, *J* = 9.4 Hz, 1H, 3-H), 5.66 (t, *J* = 7.4 Hz, 1H, 2’-H), 4.68 (s, 2H, 4’-H), 3.92 (s, 3H, OCH_3_), 3.64 (d, *J* = 7.3 Hz, 2H, 1’-H), 1.96 (s, 3H, 5’-H); ^13^C NMR (101 MHz, CDCl_3_) δ: 165.7, 161.2, 160.2, 152.8, 143.7, 131.6×2, 131.1×2, 130.9, 129.3, 127.9, 126.6, 126.5, 116.5, 113.1, 113.0, 107.3, 70.8, 56.0, 21.6, 14.2; HRESIMS m/z 465.0302 [M+Na]^+^ (calcd for C_22_H_19_BrO_5_Na, 465.0308).

**4k**: Yield = 50%, white solid, mp 114-116 °C; ^1^H NMR (600 MHz, CDCl_3_) δ: 7.62 (d, *J* = 9.4 Hz, 1H, 4-H), 7.41 (d, *J* = 8.4 Hz, 2H, ArH), 7.32 (d, *J* = 8.5 Hz, 1H, 5-H), 7.14 (d, *J* = 8.4 Hz, 2H, ArH), 6.84 (d, *J* = 8.6 Hz, 1H, 6-H), 6.25 (d, *J* = 9.4 Hz, 1H, 3-H), 5.52 (t, *J* = 6.6 Hz, 1H, 2’-H), 4.45 (s, 2H, 2’-H), 3.90 (s, 3H, OCH_3_), 3.59 (d, *J* = 7.3 Hz, 2H,, 1’-H), 3.57 (s, 2H, ArCH2), 1.84 (s, 3H, 5’-H); ^13^C NMR (101 MHz, CDCl_3_) δ: 170.9, 161.2, 160.2, 152.8, 143.7, 133.0, 131.6×2, 131.0×2, 130.8, 126.6, 126.2, 121.0, 116.5, 113.1, 112.9, 107.3, 70.5, 56.0, 40.7, 21.6, 14.1；HRESIMS m/z 479.0463 [M+Na]^+^ (calcd for C_23_H_21_BrO_5_Na, 479.0464).

**4l**: Yield = 58%, white solid, mp 92-94 °C; ^1^H NMR (600 MHz, CDCl_3_) δ: 7.99 (d, *J* = 8.7 Hz, 1H, ArH), 7.84 (dd, *J* = 7.8, 1.6 Hz, 1H, ArH), 7.79-7.74 (m, 1H, ArH), 7.62 (d, *J* = 9.4 Hz, 1H, 4-H), 7.52-7.44 (m, 2H, ArH), 7.43-7.38 (m, 2H, ArH), 7.31 (d, *J* = 8.5 Hz, 1H, 5-H), 6.82 (d, *J* = 8.6 Hz, 1H, 6-H), 6.25 (d, *J* = 9.4 Hz, 1H, 3-H), 5.50 (t, *J* = 6.5 Hz, 1H, 2’-H), 4.45 (s, 2H, 4’-H), 4.07 (s, 2H, ArCH2), 3.87 (s, 3H, OCH_3_), 3.56 (d, *J* = 7.3 Hz, 2H, 1’-H), 1.77 (s, 3H, 5’-H); ^13^C NMR (101 MHz, CDCl_3_) δ: 171.4, 161.2, 160.2, 152.8, 143.7, 133.7, 132.1, 130.9, 130.6, 128.6, 128.0, 128.0, 126.6, 126.3, 126.1, 125.7, 125.4, 123.8, 116.6, 113.0, 112.9, 107.3, 70.5, 56.0, 39.2, 21.6, 14.0; HRESIMS m/z 451.1519 [M+Na]^+^ (calcd for C_27_H_24_O_5_Na, 451.1515).

**4m**: Yield = 56%, white solid, mp 164-166 °C; ^1^H NMR (600 MHz, CDCl_3_) δ: 9.01 (s, 1H, NH), 7.68 (dd, *J* = 8.0, 1.1 Hz, 1H, Indole-H), 7.62 (d, *J* = 9.4 Hz, 1H, 4-H), 7.44 (dd, *J* = 8.3, 1.1 Hz, 1H, Indole-H), 7.35-7.28 (m, 2H, Indole-H), 7.24 (dd, *J* = 2.1, 1.0 Hz, 1H, Indole-H), 7.14 (td, *J* = 7.5, 1.0 Hz, 1H, Indole-H), 6.85 (d, *J* = 8.6 Hz, 1H, 5-H), 6.26 (d, *J* = 9.4 Hz, 1H, 3-H), 5.67 (t, *J* = 7.1 Hz, 1H, 2’-H), 4.71 (s, 2H, 4’-H), 3.92 (s, 3H, OCH_3_), 3.65 (d, *J* = 7.3 Hz, 2H, 1’-H), 1.99 (s, 3H, 5’-H); ^13^C NMR (101 MHz, CDCl_3_) δ:161.8, 161.3, 160.2, 152.9, 143.8, 136.9, 130.9, 127.4, 127.3, 126.7, 126.6, 125.3, 122.5, 120.7, 116.5, 113.1, 113.0, 112.0, 108.8, 107.4, 70.5, 56.1, 21.7, 14.3; HRESIMS m/z 426.1305[M+Na]^+^ (calcd for C_24_H_21_NO_5_Na, 426.1311).

**4n**: Yield = 58%, white solid, mp 126-128 °C; ^1^H NMR (600 MHz, CDCl_3_) δ: 9.22 (s, 1H, ArH), 8.77 (d, *J* = 3.1 Hz, 1H, ArH), 8.30 (dt, *J* = 7.9, 2.0 Hz, 1H, ArH), 7.63 (d, *J* = 9.5 Hz, 1H, 4-H), 7.39 (dd, *J* = 7.9, 4.8 Hz, 1H, ArH), 7.33 (d, *J* = 8.6 Hz, 1H, 5-H), 6.85 (d, *J* = 8.6 Hz, 1H, 6-H), 6.25 (d, *J* = 9.4 Hz, 1H, 3-H), 5.67 (t, *J* = 6.6 Hz, 1H, 2’-H), 4.72 (s, 1H, 4’-H), 3.93 (s, 3H, OCH_3_), 3.65 (d, *J* = 7.3 Hz, 2H, 1’-H), 1.98 (s, 3H, 5’-H); ^13^C NMR (101 MHz, CDCl_3_) δ: 165.1, 161.2, 160.2, 153.3, 152.8, 150.9, 143.7, 137.1, 130.7, 126.8, 126.7, 126.2, 123.3, 116.4, 113.1, 113.0, 107.3, 71.0, 56.1, 21.6, 14.2; HRESIMS m/z 388.1149 [M+Na]^+^ (calcd for C_21_H_19_NO_5_Na, 388.1155).

**4o**: Yield = 54%, white solid, mp 122-124 °C; ^1^H NMR (600 MHz, CDCl_3_) δ: 8.77 (d, *J* = 6.0 Hz, 2H, ArH), 7.85 (d, *J* = 6.0 Hz, 2H, ArH), 7.63 (d, *J* = 9.5 Hz, 1H, 4-H), 7.33 (d, *J* = 8.6 Hz, 1H, 5-H), 6.85 (d, *J* = 8.6 Hz, 1H, 6-H), 6.25 (d, *J* = 9.5 Hz, 1H, 3-H), 5.68 (t, *J* = 7.0 Hz, 1H, 2’-H), 4.72 (s, 2H, 4’-H), 3.93 (s, 3H, OCH_3_), 3.65 (d, *J* = 7.3 Hz, 2H, 1’-H), 1.98 (s, 3H, 5’-H); ^13^C NMR (101 MHz, CDCl_3_) δ:164.9, 161.2, 160.2, 152.8, 150.5×2, 143.7, 137.5, 130.5, 127.1, 126.7, 122.9×2, 116.4, 113.1, 113.0, 107.3, 71.4, 56.0, 21.7, 14.2; HRESIMS m/z 388.1151 [M+Na]^+^ (calcd for C_21_H_19_NO_5_Na, 388.1155).

**4p**: Yield = 65%, white solid, mp 140-142 °C; ^1^H NMR (600 MHz, CDCl_3_) δ: 8.01 (s, 1H, Furan-H), 7.63 (d, *J* = 9.4 Hz, 1H, 4-H), 7.41 (s, 1H, Furan-H), 7.32 (d, *J* = 8.5 Hz, 1H, 5-H), 6.85 (d, *J* = 8.7 Hz, 1H, 6-H), 6.74 (s, 1H, Furan-H), 6.25 (d, *J* = 9.4 Hz, 1H, 3-H), 5.64 (t, *J* = 7.2 Hz, 1H, 2’-H), 4.62 (s, 2H, 4’-H), 3.92 (s, 3H, OCH_3_), 3.63 (d, *J* = 7.3 Hz, 2H, 1’-H), 1.93 (s, 3H, 5’-H); ^13^C NMR (101 MHz, CDCl_3_) δ: 163.0, 161.2, 160.2, 152.8, 147.7, 143.7, 143.6, 131.0, 126.6, 126.2, 119.4, 116.6, 113.0, 113.0, 109.8, 107.3, 70.0, 56.0, 21.6, 14.1; HRESIMS m/z 377.0998 [M+Na]^+^ (calcd for C_20_H_18_O_6_Na, 377.0995).

**4q**: Yield = 55%, white solid, mp 147-149 °C; ^1^H NMR (600 MHz, CDCl_3_) δ: 7.79 (d, *J* = 4.3 Hz, 1H, Thiophene-H), 7.63 (d, *J* = 9.4 Hz, 1H, 4-H), 7.54 (d, *J* = 5.0 Hz, 1H, Thiophene-H), 7.32 (d, *J* = 8.6 Hz, 1H, 5-H), 7.09 (t, *J* = 4.4 Hz, 1H, Thiophene-H), 6.85 (d, *J* = 8.6 Hz, 1H, 6-H), 6.25 (d, *J* = 9.4 Hz, 1H, 3-H), 5.65 (t, *J* = 7.4 Hz, 1H, 2’-H), 4.66 (s, 2H, 4’-H), 3.92 (s, 3H, OCH_3_), 3.63 (d, *J* = 7.4 Hz, 2H, 1’-H), 1.95 (s, 3H, 5’-H); 13C NMR (101 MHz, CDCl_3_) δ:162.1, 161.2, 160.3, 152.8, 143.7, 133.9, 133.3, 132.2, 130.9, 127.7, 126.6, 126.3, 116.6, 113.1, 113.0, 107.3, 70.6, 56.0, 21.6, 14.1; HRESIMS m/z 393.0762 [M+Na]^+^ (calcd for C_20_H_18_O_5_S Na, 393.0767).

**4r**: Yield = 64%, white solid, mp 128-130 °C; ^1^H NMR (600 MHz, CDCl_3_) δ: 7.62 (d, *J* = 9.4 Hz, 1H, 4-H), 7.32 (d, *J* = 8.6 Hz, 1H, 5-H), 7.11 (d, *J* = 3.5 Hz, 1H, Furan-H), 6.85 (d, *J* = 8.6 Hz, 1H, 6-H), 6.44 (d, *J* = 3.5 Hz, 1H, Furan-H), 6.24 (d, *J* = 9.4 Hz, 1H, 3-H), 5.64 (t, *J* = 7.1 Hz, 1H, 2’-H), 4.66 (s, 2H, 4’-H), 3.93 (s, 3H, OCH_3_), 3.62 (d, *J* = 7.4 Hz, 2H, 1’-H), 1.94 (s, 3H, 5-H); ^13^C NMR (101 MHz, CDCl_3_) δ: 161.1, 160.2, 157.5, 152.8, 146.3, 143.7, 130.6, 127.4, 126.8, 126.6, 120.0, 116.5, 113.8, 113.0, 113.0, 107.3, 70.6, 56.0, 21.6, 14.1; HRESIMS m/z 455.0096 [M+Na]^+^ (calcd for C_20_H_17_BrO_6_Na, 455.0100).

**Anti-acetylcholinesterase *Assay in vitro***

The anti-AChE activity of the 7-diethylaminocoumarin-based 1,3,4-oxadiazole derivatives was determined by the method of Ellman. The *in vitro* inhibition assays of AChE from electric eel run in phosphate buffer 0.1 M, at pH 7.4. Acetylthiocholine iodide was used as substrates, and 5,5′-Dithiobis(2-nitrobenzoic acid) (DTNB) was used as the chromophoric reagent. To a 96-well plate of the sample solution (10 μl), phosphate buffer solution (PBS) (40 μl, 0.1 M, pH = 7.4), DTNB (20 μl, 2.5 mM in 0.1 M PBS, pH = 8.0) and AChE solution (10 μl, 0.2 U/ml in 0.1 M PBS, pH = 7.4) were added sequentially, shaken well, and incubated at 37 °C for 10 min. Then, acetylthiocholine iodide (20 μl, 10 mM in 0.1 M PBS, pH = 7.4) was added, shaken well again, and incubated at 37 °C for 10 min. Assays were carried out with a blank containing all components except AChE in order to account for non-enzymatic reaction. The absorbance at 405 nm of the samples was measured using a spectrophotometer, the sample solution was set to three different concentrations (0.01, 0.1, 1 μmol/ml ) and the experiment was repeated three times. Tacrine was used as a positive control. Inhibitory effect (%) =[(OD_0_−OD_1_)−(OD_2_−OD_3_)]/(OD_0_−OD_1_)×100%, where OD_0_ represented the absorbance of blank group; OD_1_ represented the absorbance of blank group without AChE; OD_2_ represented the absorbance of sample group; OD_3_ represented the absorbance of sample group without AChE.

**Molecular Docking**

The 3D-structure of human AChE was downloaded from the RCSB database (PDB ID: 3DHP), a chain A of the structure was used for docking study. Then, Autodock Tool was used to determine the atom types and calculate the partial charges of the protein, and the pdbqt file was generated for docking.The 2D structure of compound **4m** and **4o** were drawn by ChemDraw2019, and saved as cdx file. Then, MM2 force field in Chem3D 2019 was used to optimize the 3D structure of the ligand. Again, Autodock Tool was used to determine the atom types and calculate the partial charges of the ligand, and pdbqt file was generated for docking. Docking of the ligand was carried out using the Autodock vina 1.1.2 program. A sphere of 20 Å around the carbonyl group of Gly122 was defined as the binding site for the ligand docking and 250 confirmations was allowed. Pymol was used to analyze the docking solutions, and the conformation of lowest affinity value was chosen for further analysis in ligplot.

**Analysis of Network Pharmacology**

**Prediction of disease targets of compound 4m.** Draw compound **4m** in ChemBioDraw and save as “sdf” file format. Import this file into PharmMapper database (http://lilab.ecust.edu.cn/ pharmmapper), Swiss target prediction database (http://www.swisstargetprediction.ch), SEA database (https://sea.bkslab.org/). Input the protein names with the species limited to “Homo sapiens” and we can receive their official symbol. After these operations, proteins information of compound targets and known targets was obtained.

**Gene screening of Alzheimer disease related targets**. Using “Connective Tissue Disease-associated with Alzheimer disease” as the search keywords, we searched Geo database (https://www.ncbi.nlm.nih.gov/geo/), GeneCards database (http://lilab.ecust.edu.cn/pharmmapper/index.php) and OMIM database ([http://www.omim.org](http://www.omim.org/)) to excavate potential targets associated with Alzheimer disease.

**Construction of protein-protein interaction (PPI) network**. Using R X 64 4.0.2 software, the intersection of the compound **4m** target and related AD target was obtained, then the Venn diagram was drawn. To further identify the core regulatory targets, PPI analysis was performed by submitting overlapping targets of active compounds in AD to the STRING database ([https://string-db.org](https://string-db.org/)). The species type was set to “Homo sapiens”, the minimum interaction threshold was set to “highest confidence” (>0.7), and the rest were set as the default. Finally, the PPI result was imported into Cytoscape 3.8.0 software to construct a PPI network.

**Hub gene analysis**. Hub gene of PPI network of **4m** against AD was calculated by MCC algorithm in Cytohubba plugin of CytoScape, then the related protein targets network was constructed. Finally, the core targets of the top 20 were also exhibited.

**Go and KEGG enrichment analysis**. To clarify the role of target proteins interacting with **4m** target genes in gene function and signalling pathways, we conducted GO and KEGG enrichment analysis of potential targets of **4m** intervention AD by R Software (R 4.0.2 for Windows). Save the data results and use R software for visual analysis.

**Molecular docking**. Molecular docking was performed among the top 5 potential target proteins of AD with **4m**. The 3D structure of the target protein was downloaded from the PDB database ([https://www.rcsb.org](https://www.rcsb.org/)). The water molecules and the original ligands were removed from the target protein through PyMOL, Later, the target proteins were imported into AutoDock Tools 1.5.6 for hydrogenation, charge calculation, and non-polar hydrogen combination, and then the result was stored in PDBQT format. Finally, run AutoDock Vina using CMD command characters for molecular docking, and use PyMOL to visualize the results.


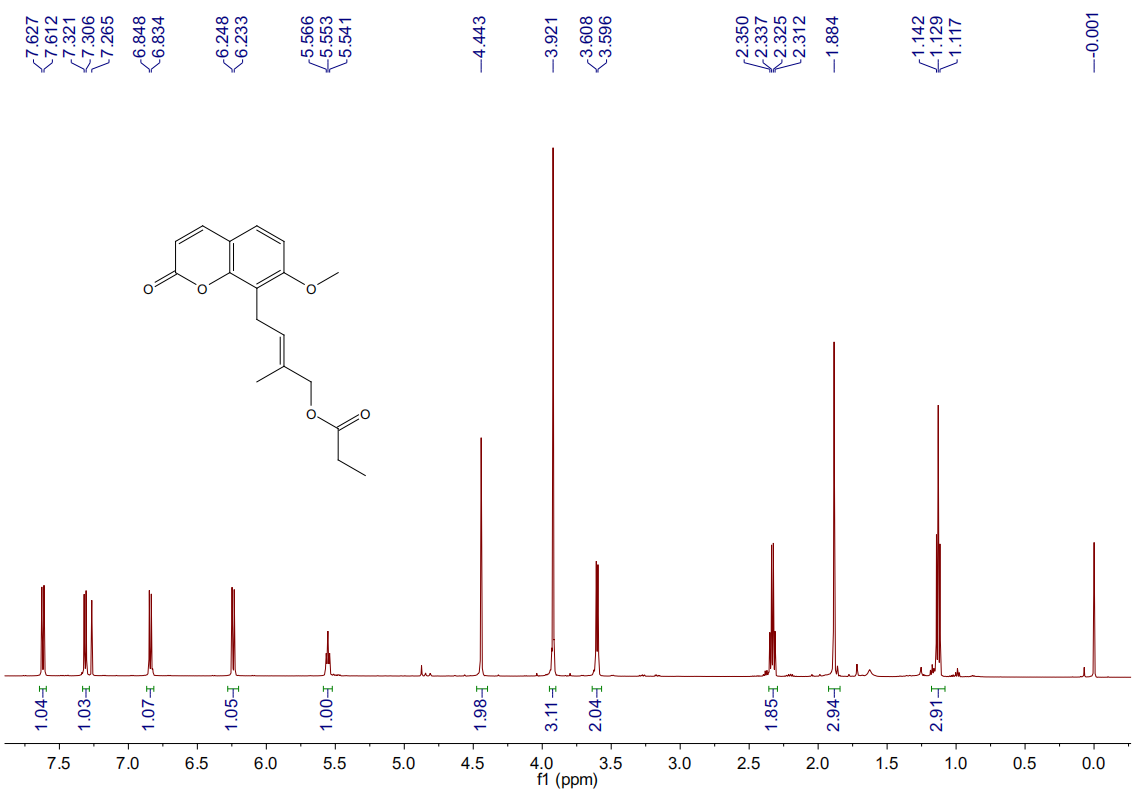


**Figure S1**. ^1^H NMR spectrum of compound **4a**


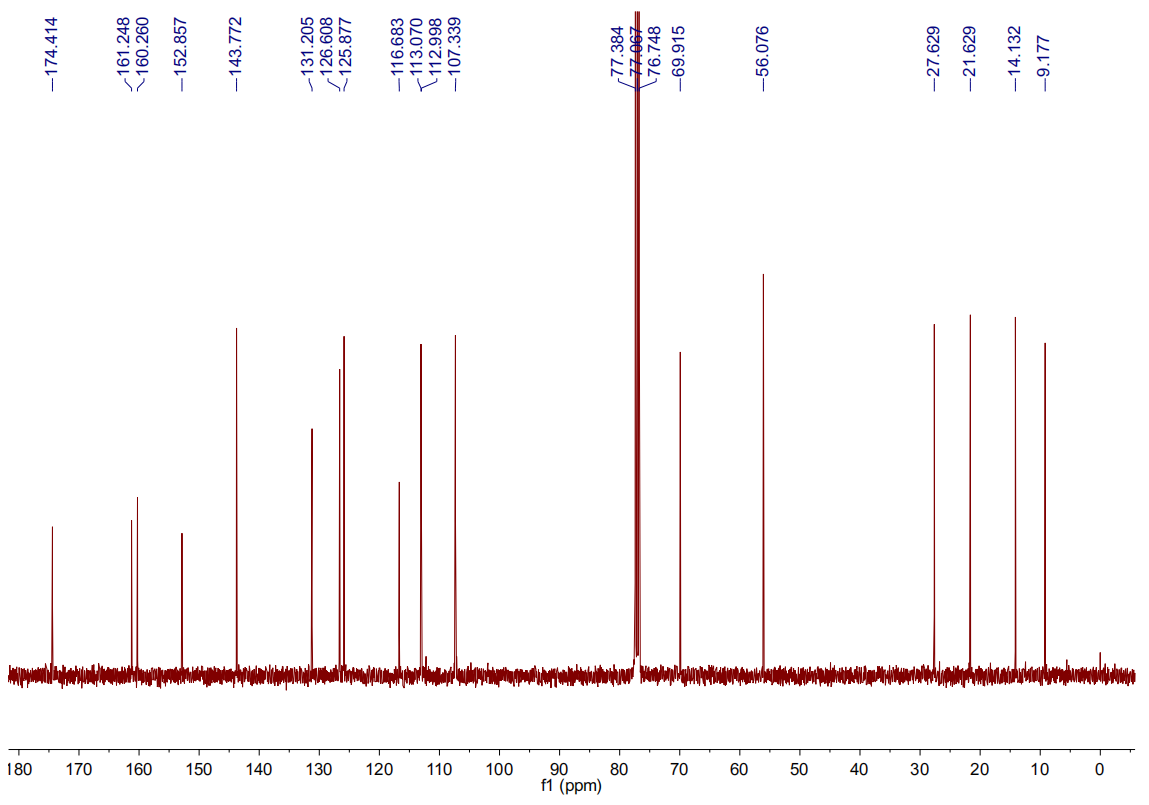


**Figure S2**. ^13^C NMR spectrum of compound **4a**


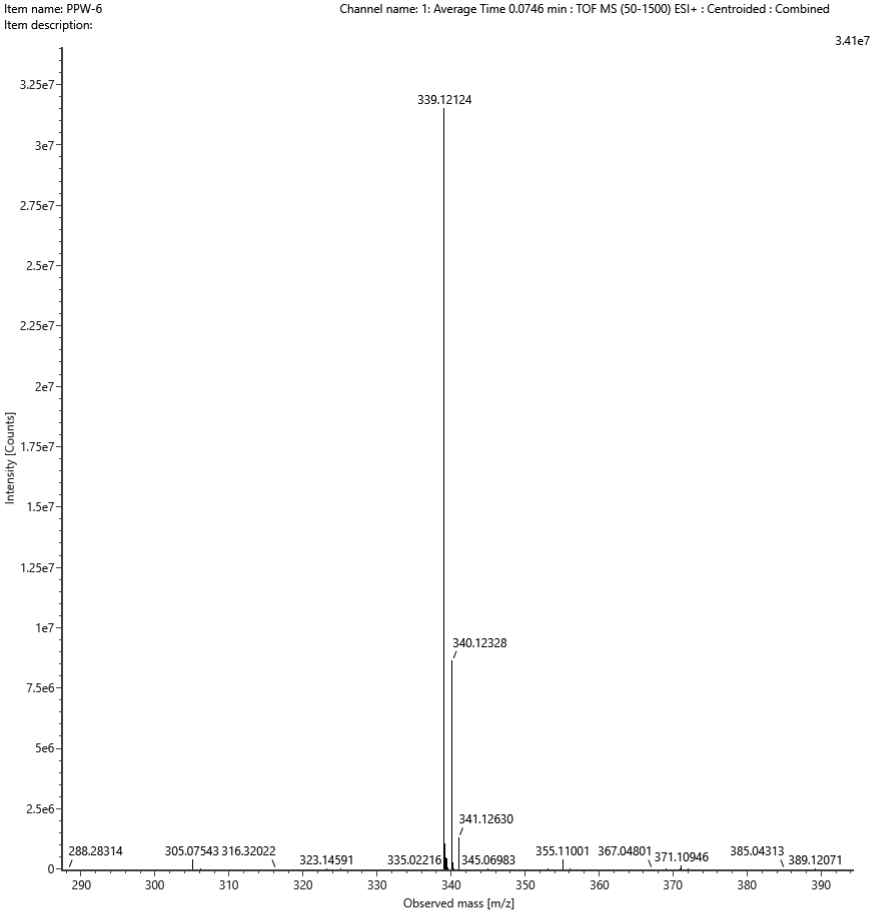


**Figure S3**. HRMS spectrum of compound **4a**


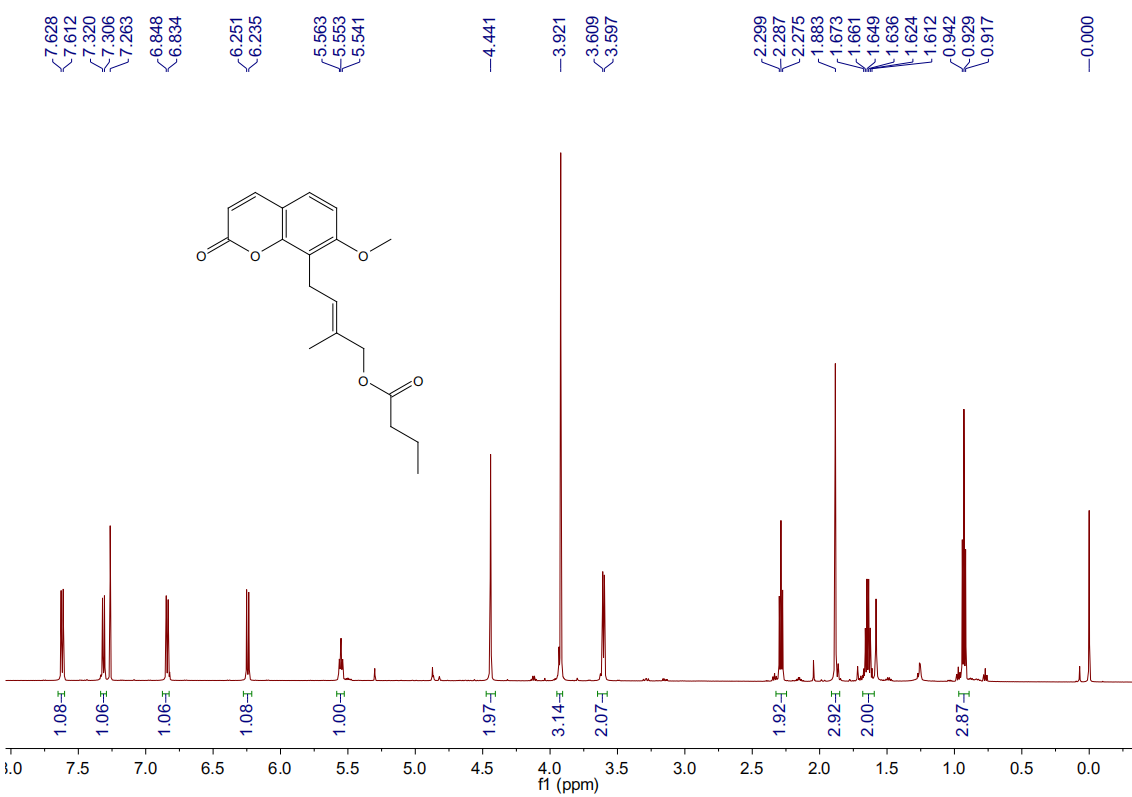


**Figure S4**. ^1^H NMR spectrum of compound **4b**


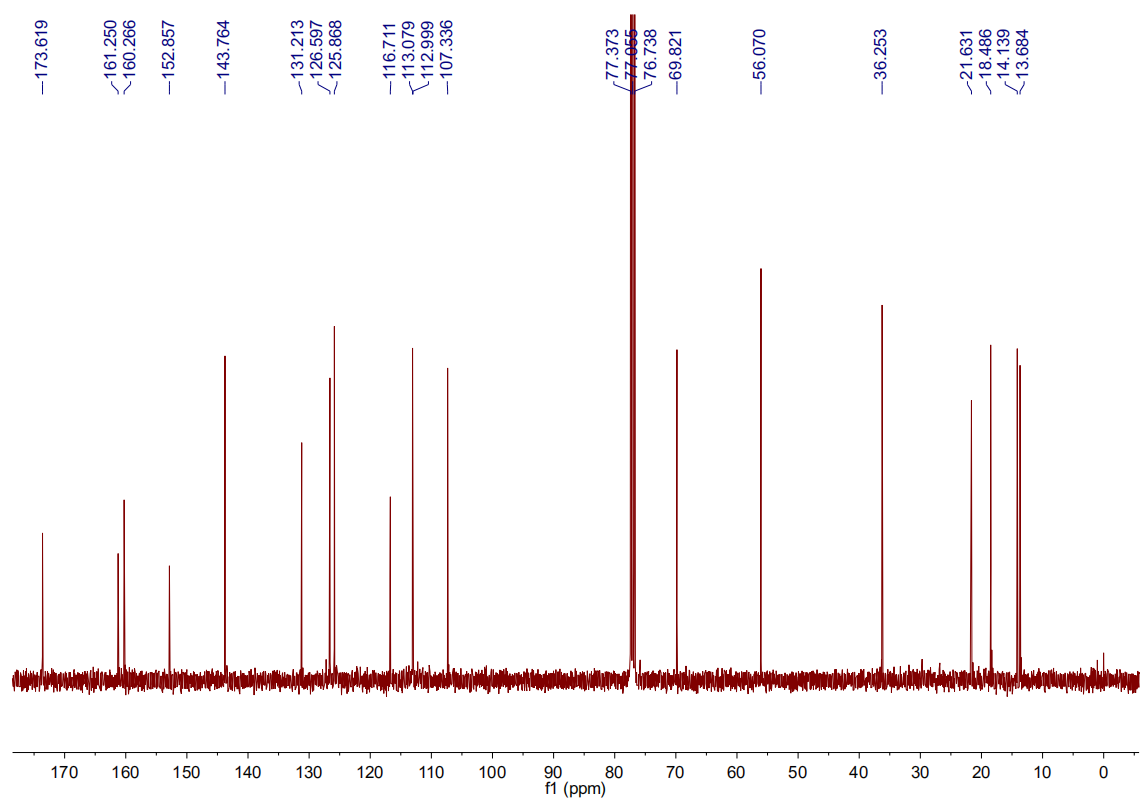


**Figure S5**. ^13^C NMR spectrum of compound **4b**


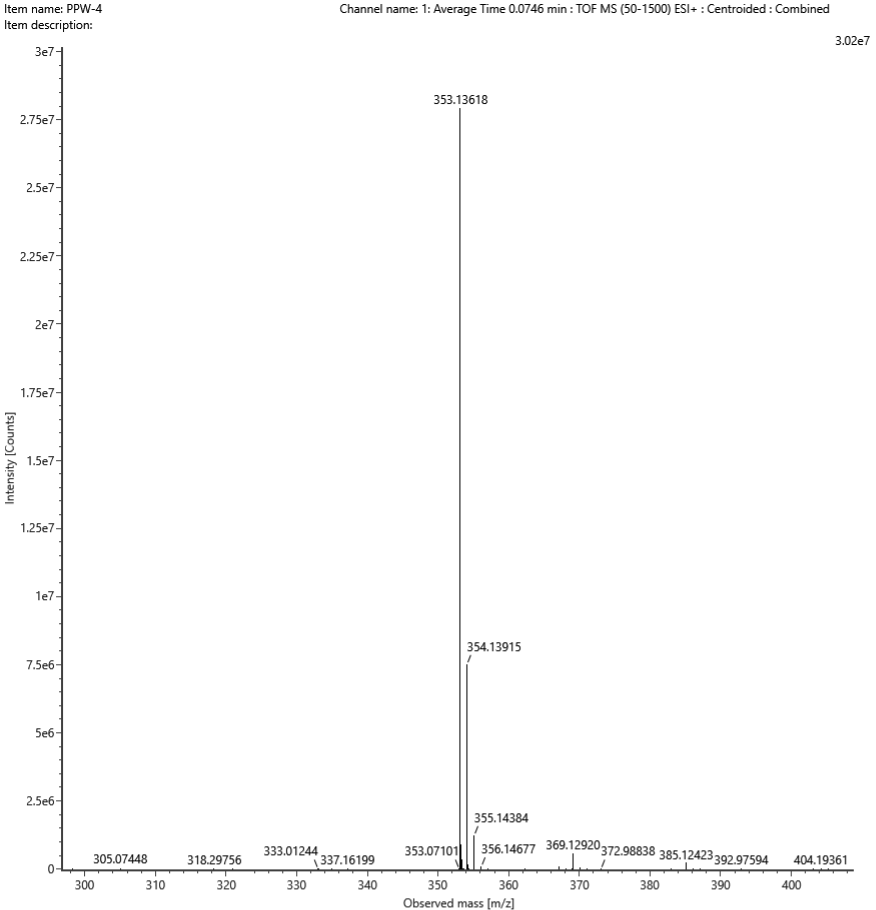


**Figure S6**. HRMS spectrum of compound **4b**


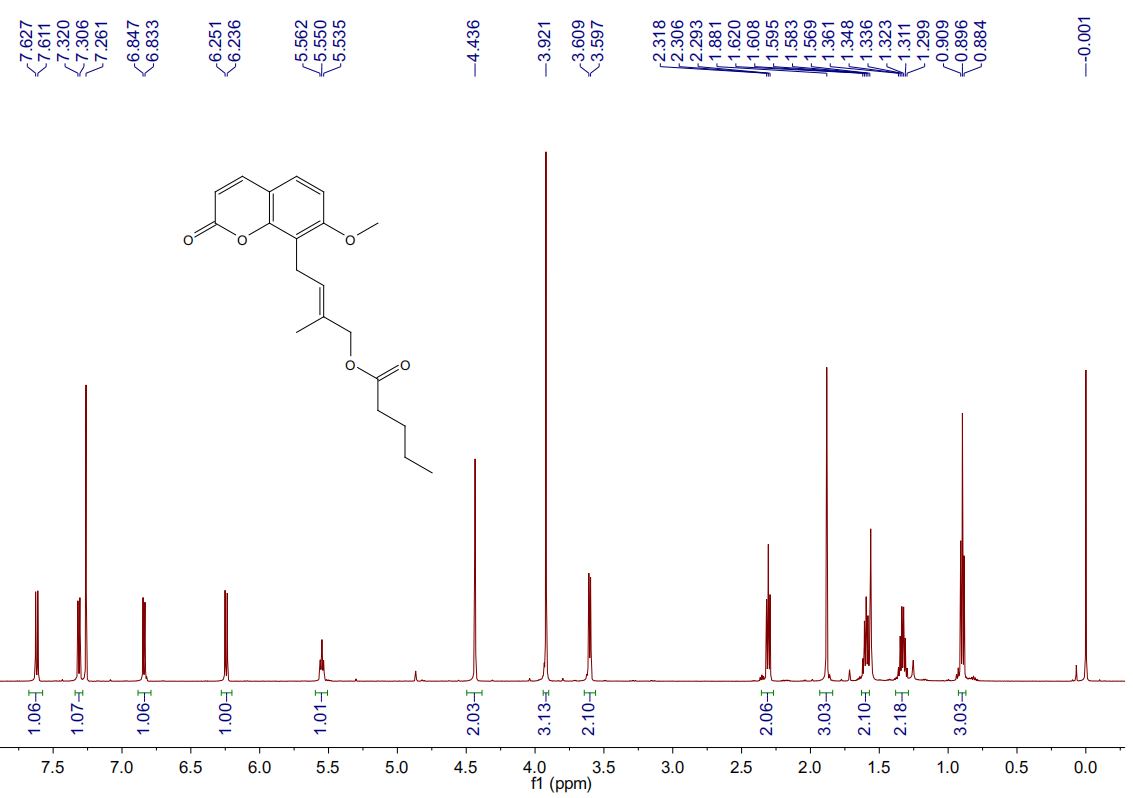


**Figure S7**. ^1^H NMR spectrum of compound **4c**


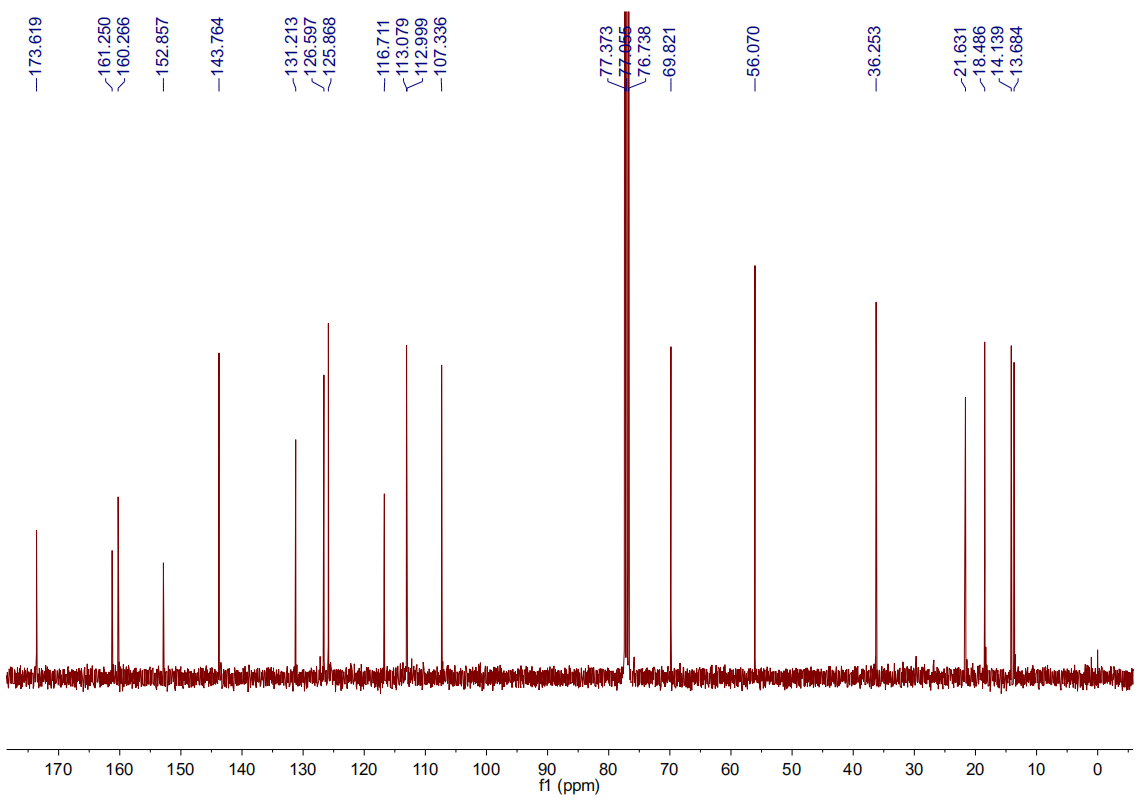


**Figure S8**. ^13^C NMR spectrum of compound **4c**


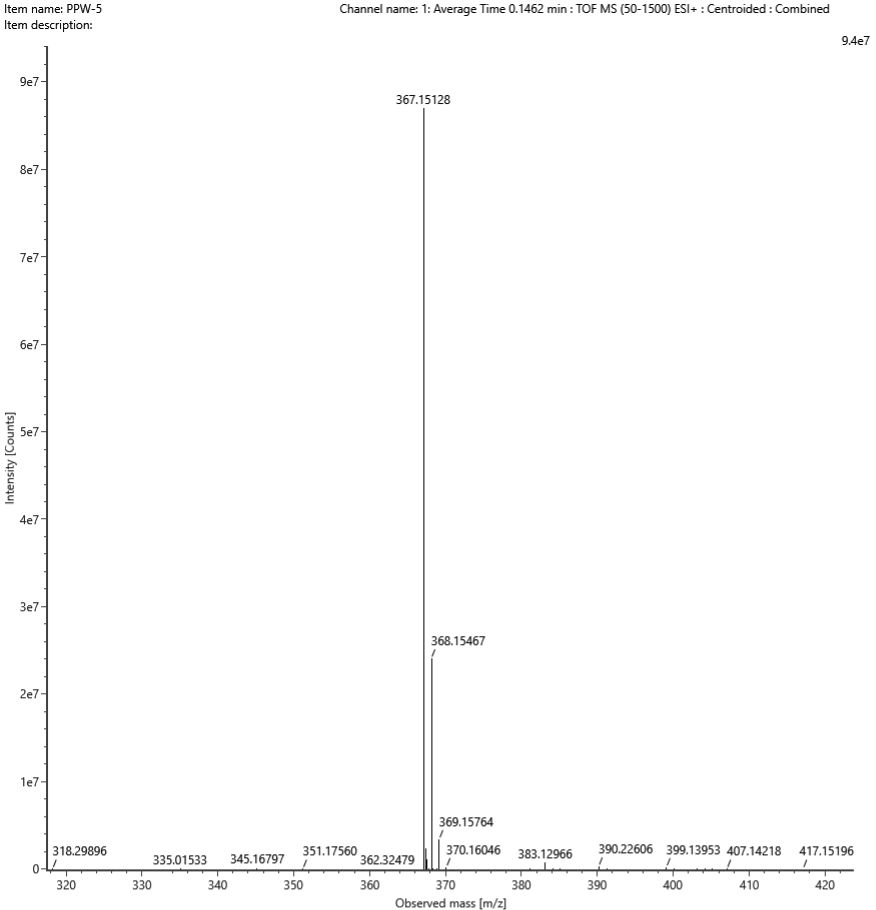


**Figure S9**. HRMS spectrum of compound **4c**


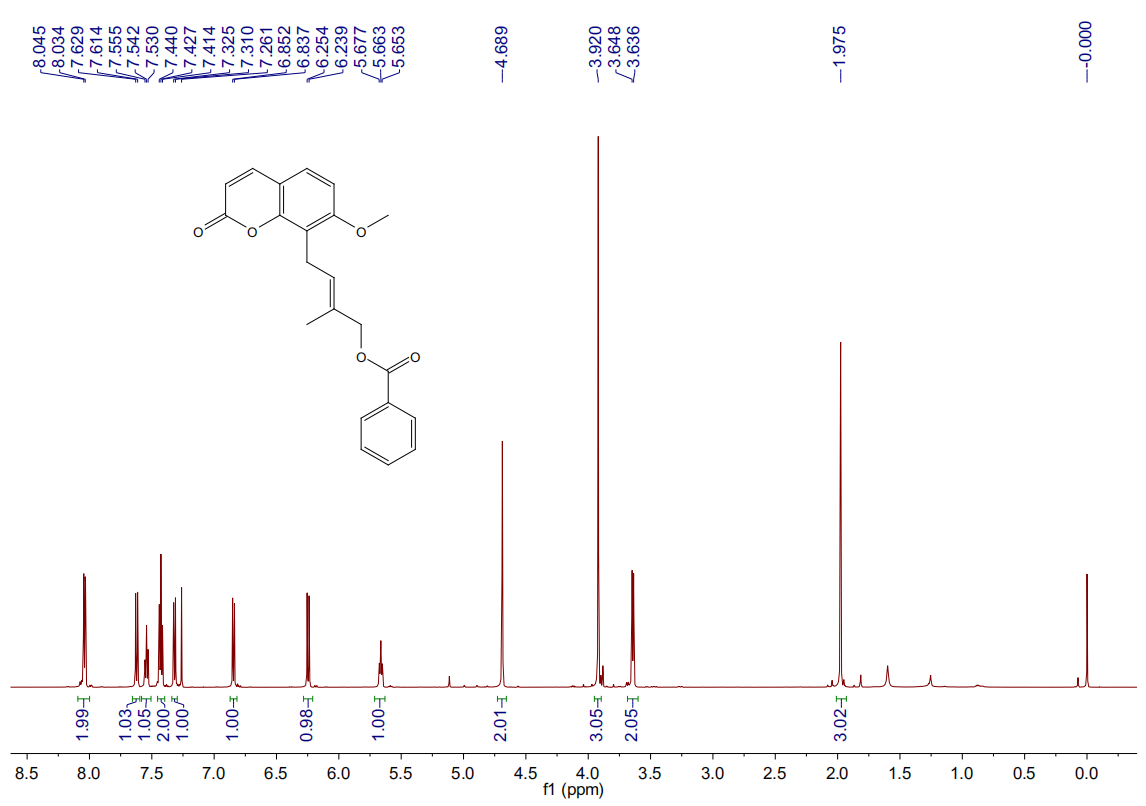


**Figure S10**. ^1^H NMR spectrum of compound **4d**


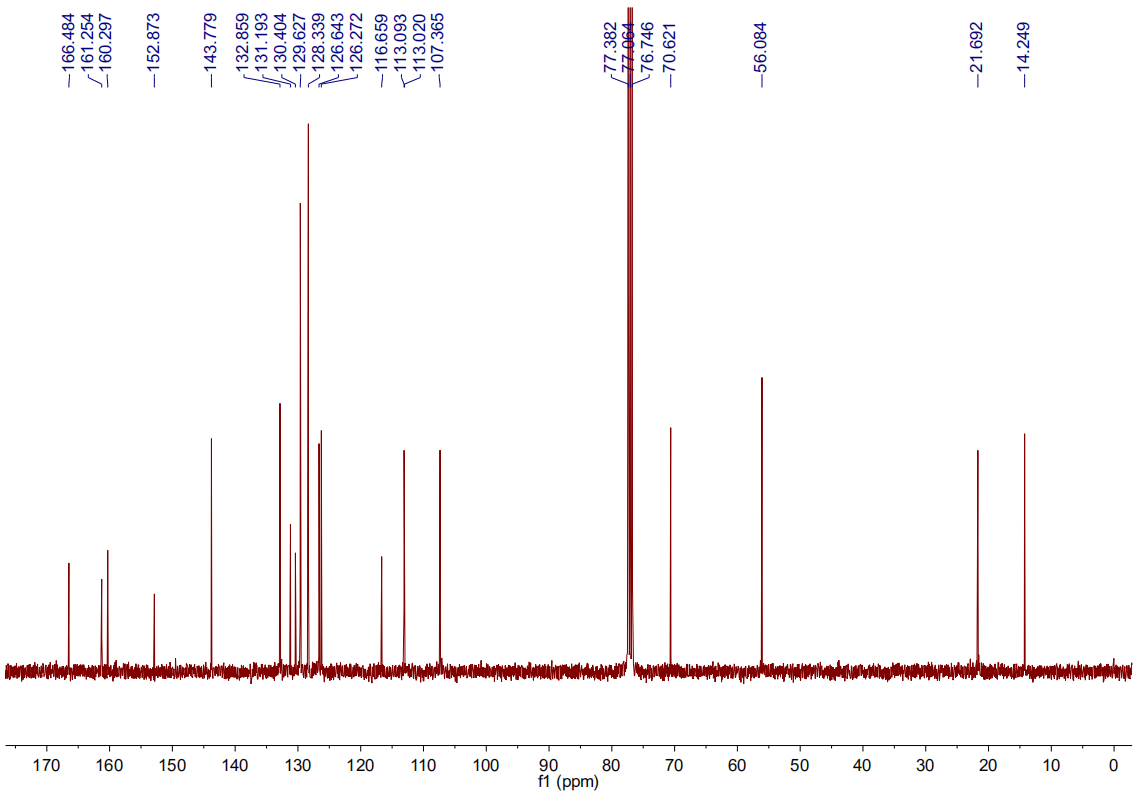


**Figure S11**. ^13^C NMR spectrum of compound **4d**


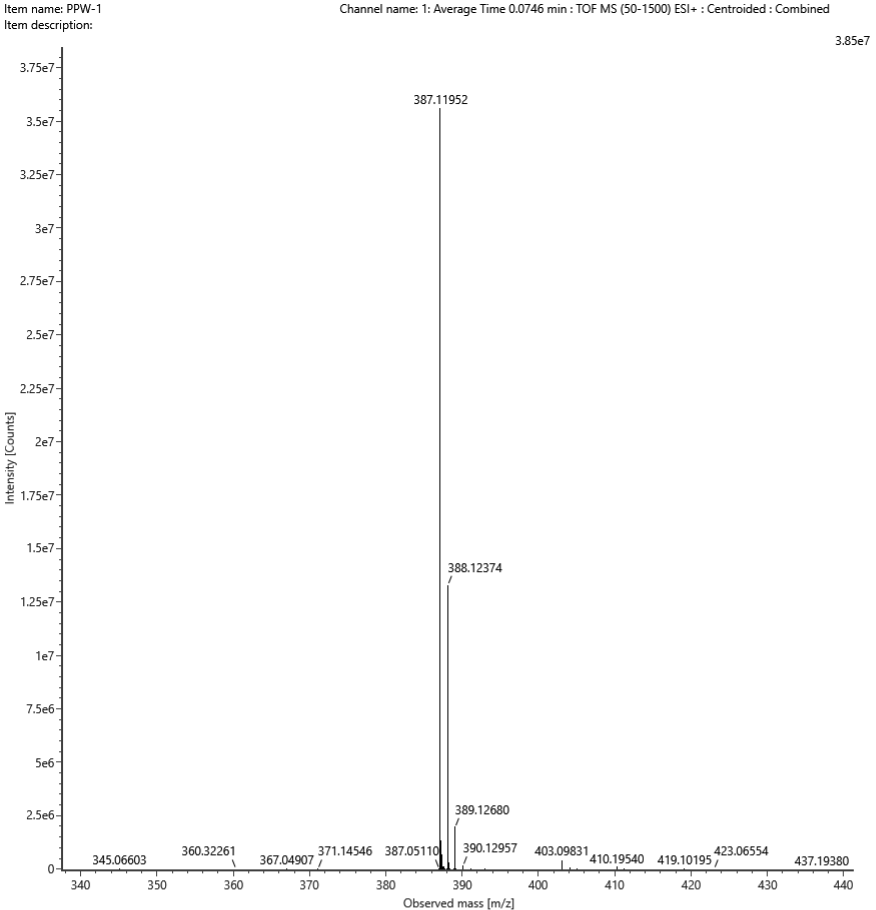


**Figure S12**. HRMS spectrum of compound **4d**


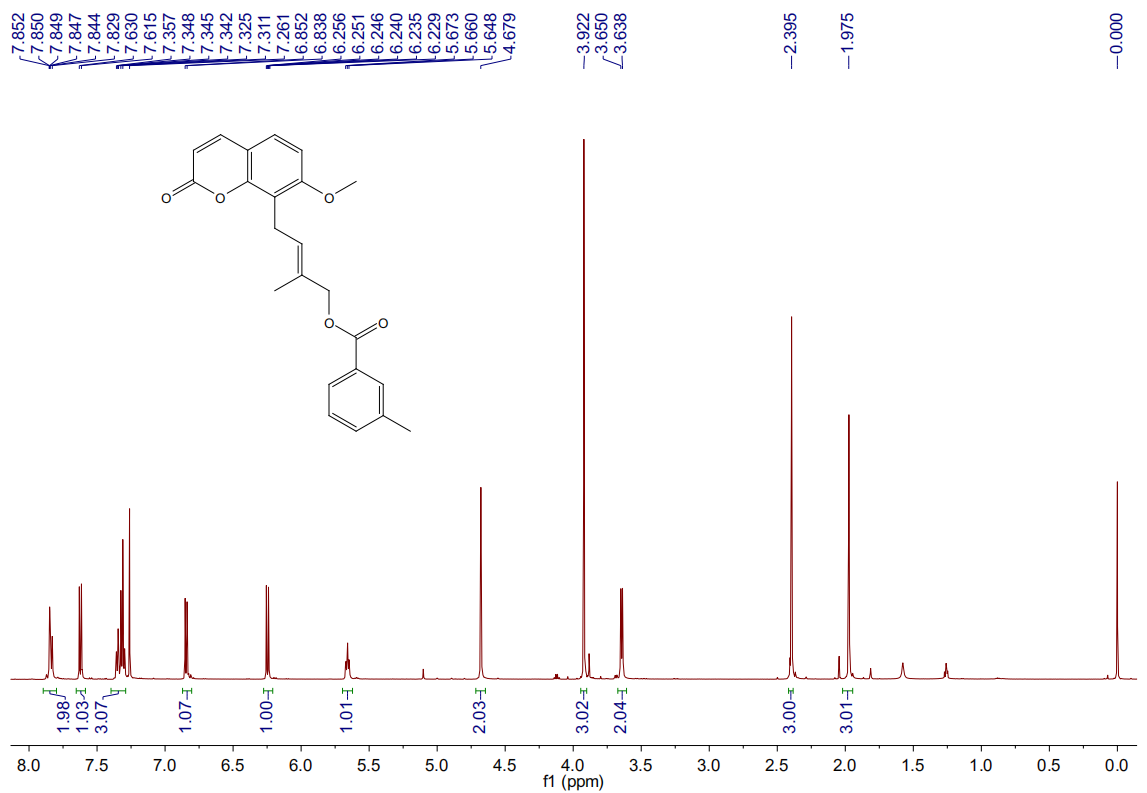


**Figure S13**. ^1^H NMR spectrum of compound **4e**


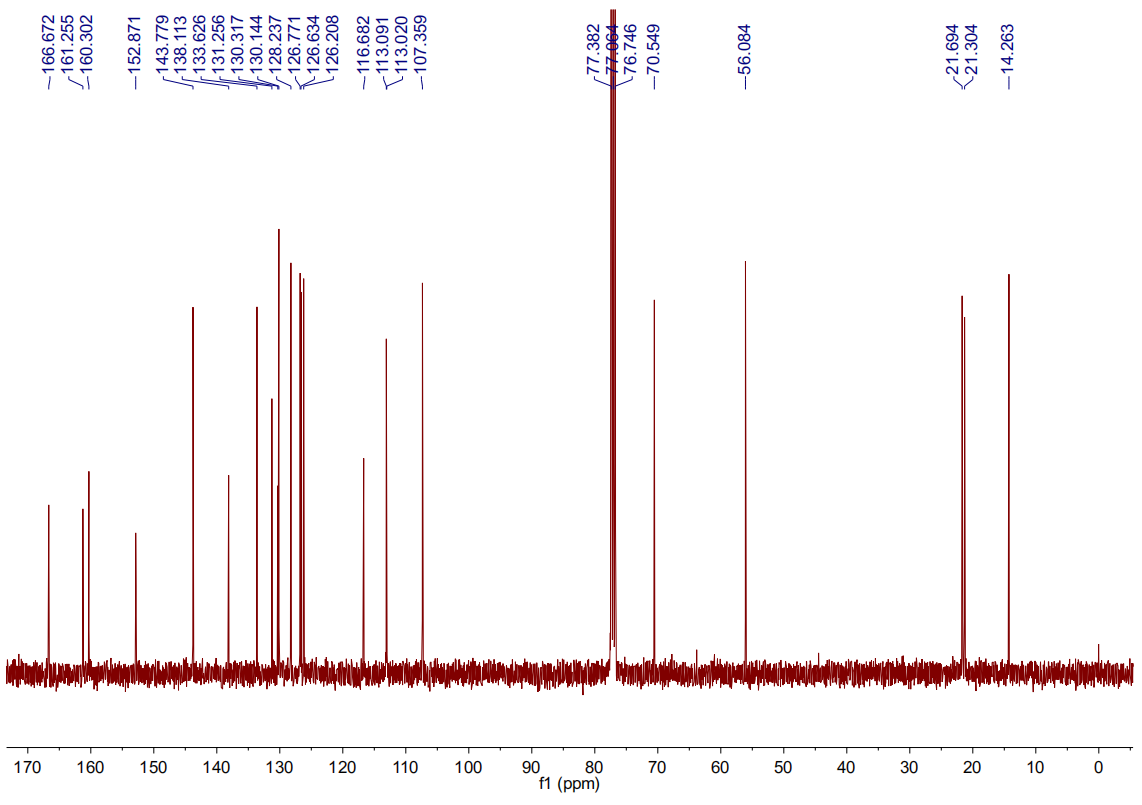


**Figure S14**. ^13^C NMR spectrum of compound **4e**


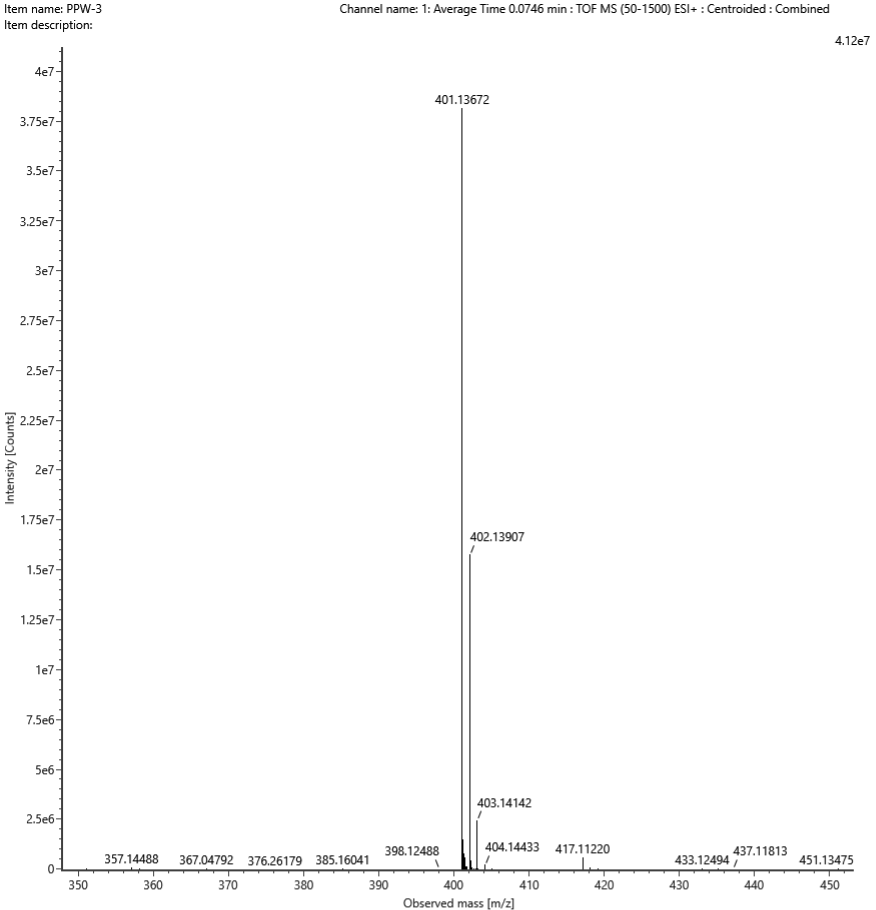


**Figure S15**. HRMS spectrum of compound **4e**


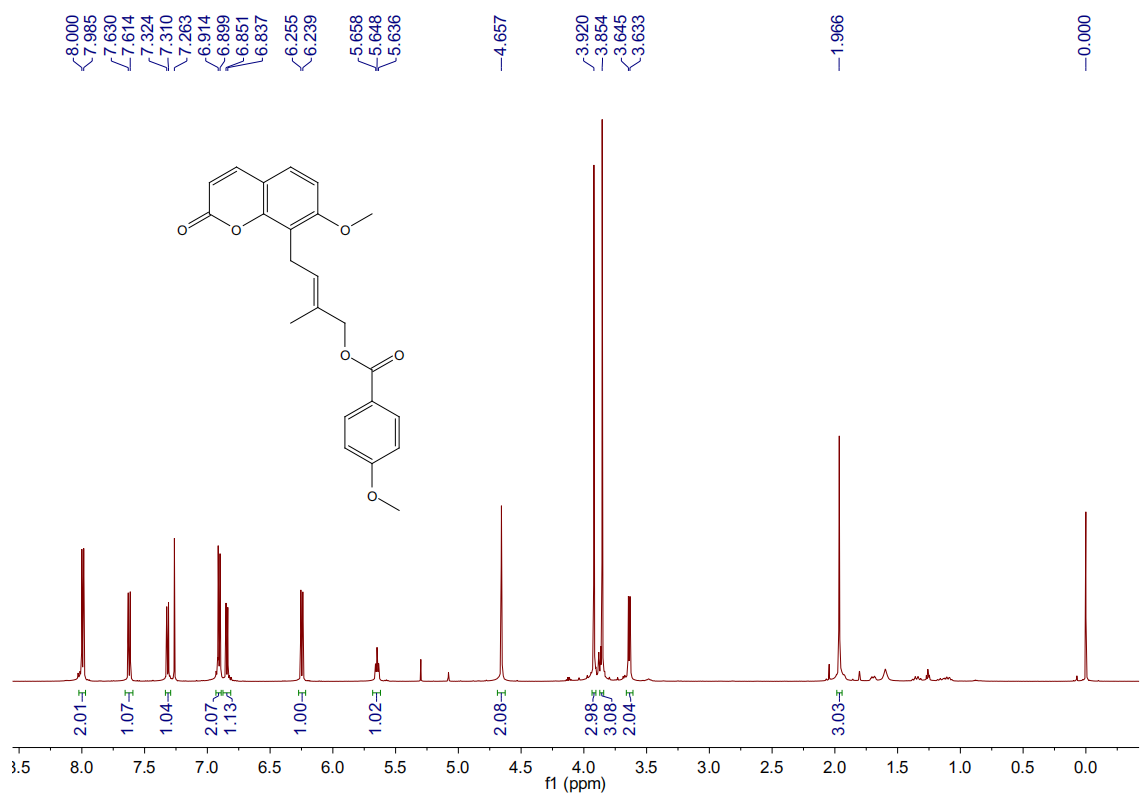


**Figure S16**. ^1^H NMR spectrum of compound **4f**


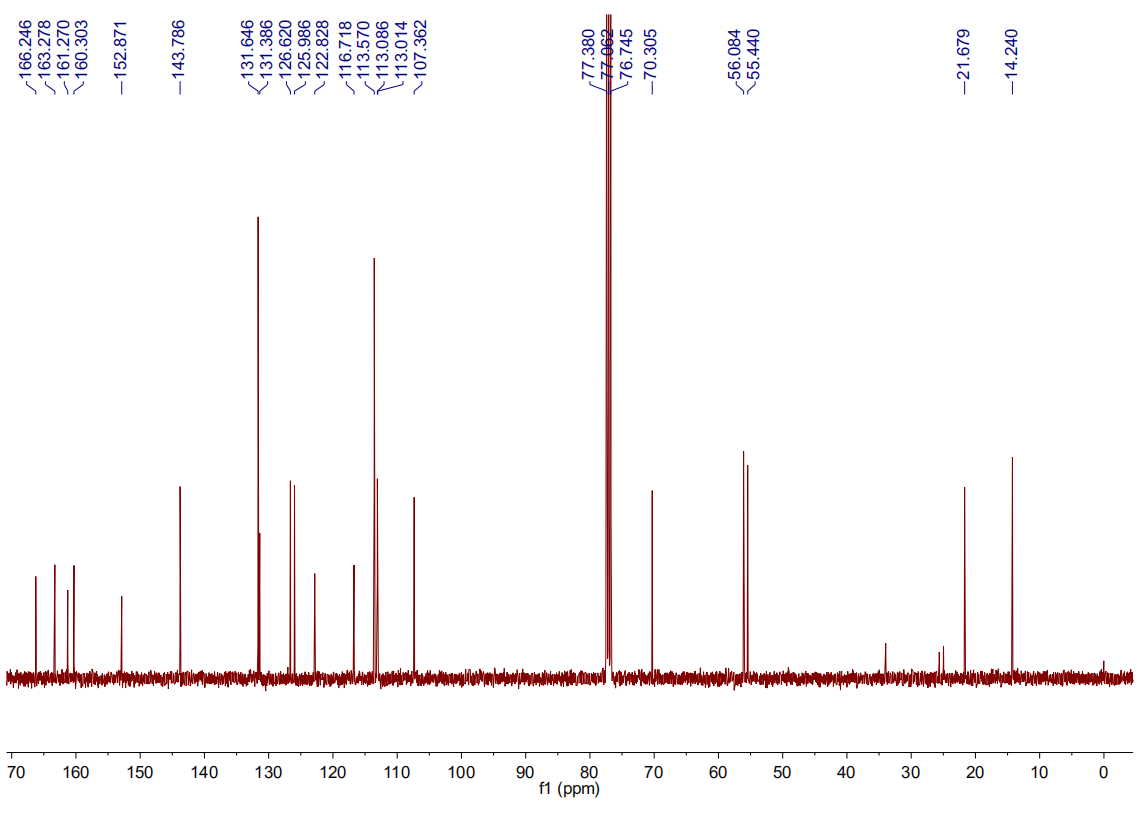


**Figure S17**. ^13^C NMR spectrum of compound **4f**


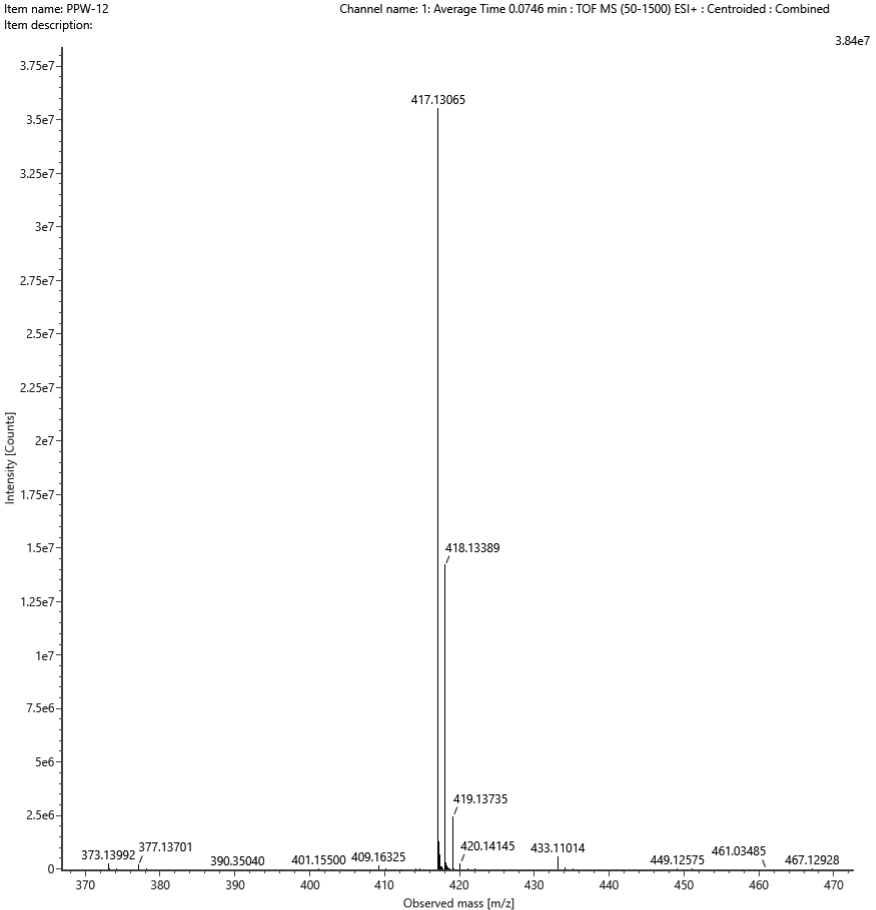


**Figure S18**. HRMS spectrum of compound **4f**


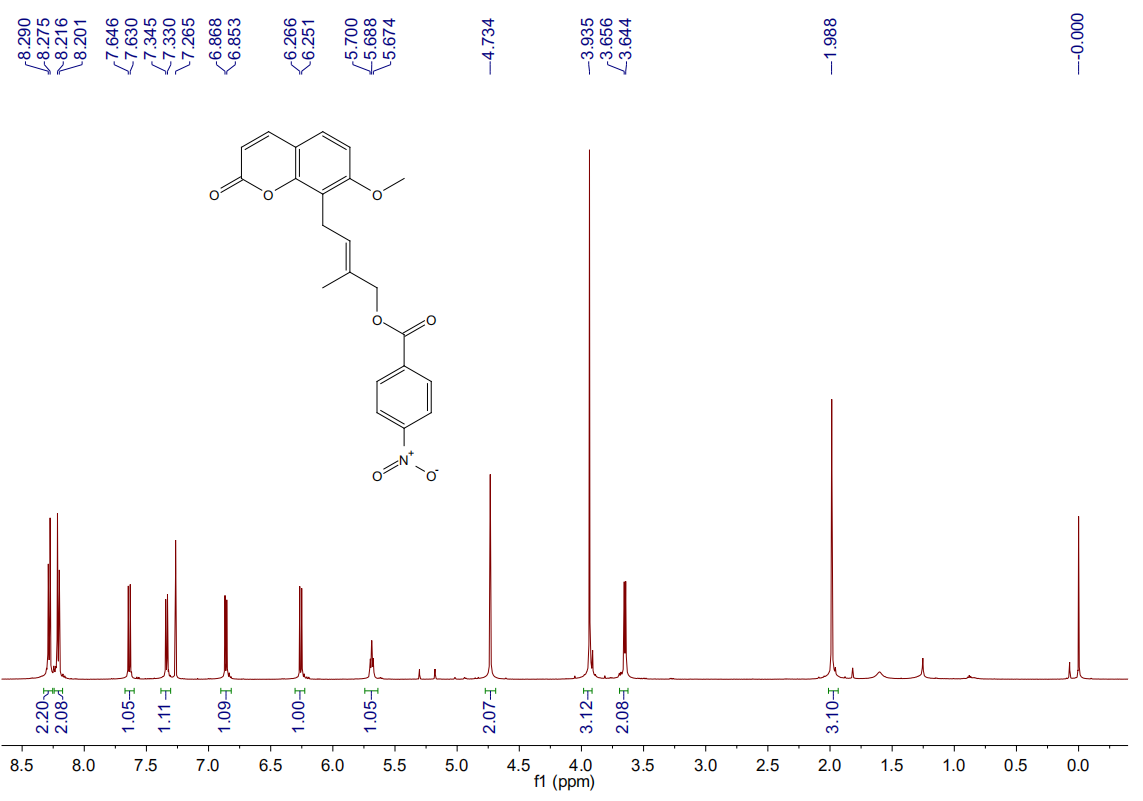


**Figure S19**. ^1^H NMR spectrum of compound **4g**


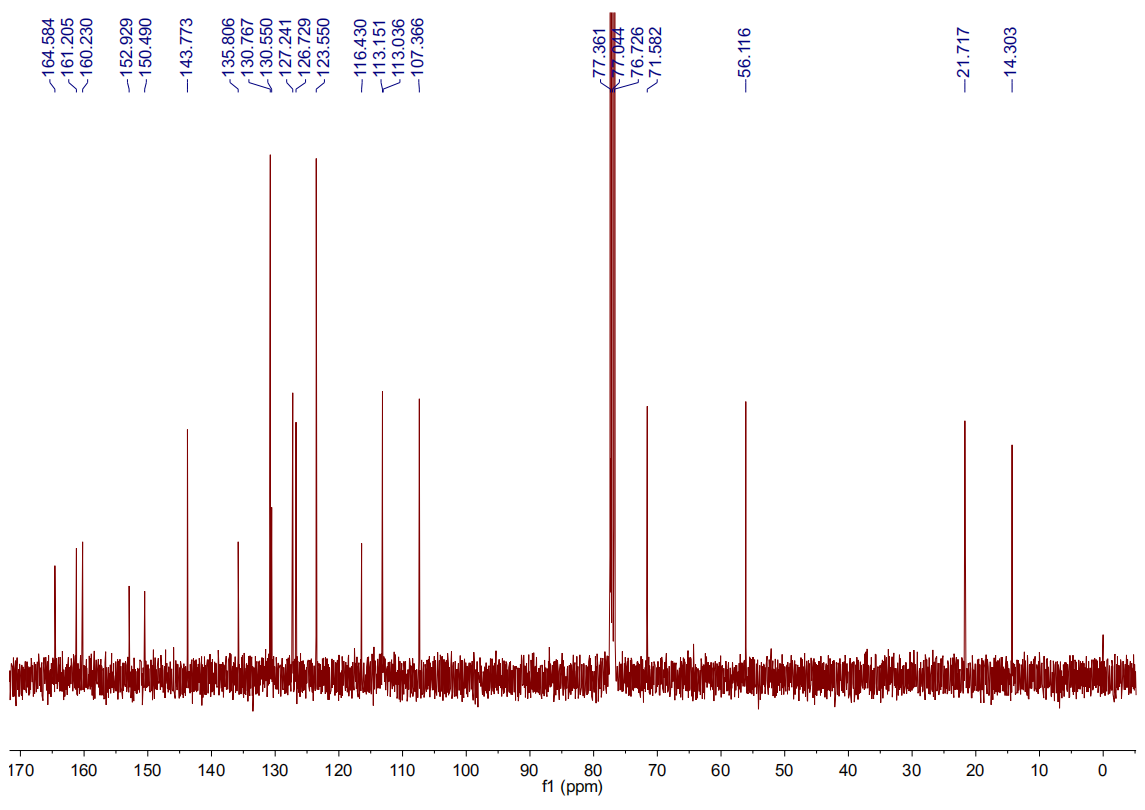


**Figure S20**. ^13^C NMR spectrum of compound **4g**


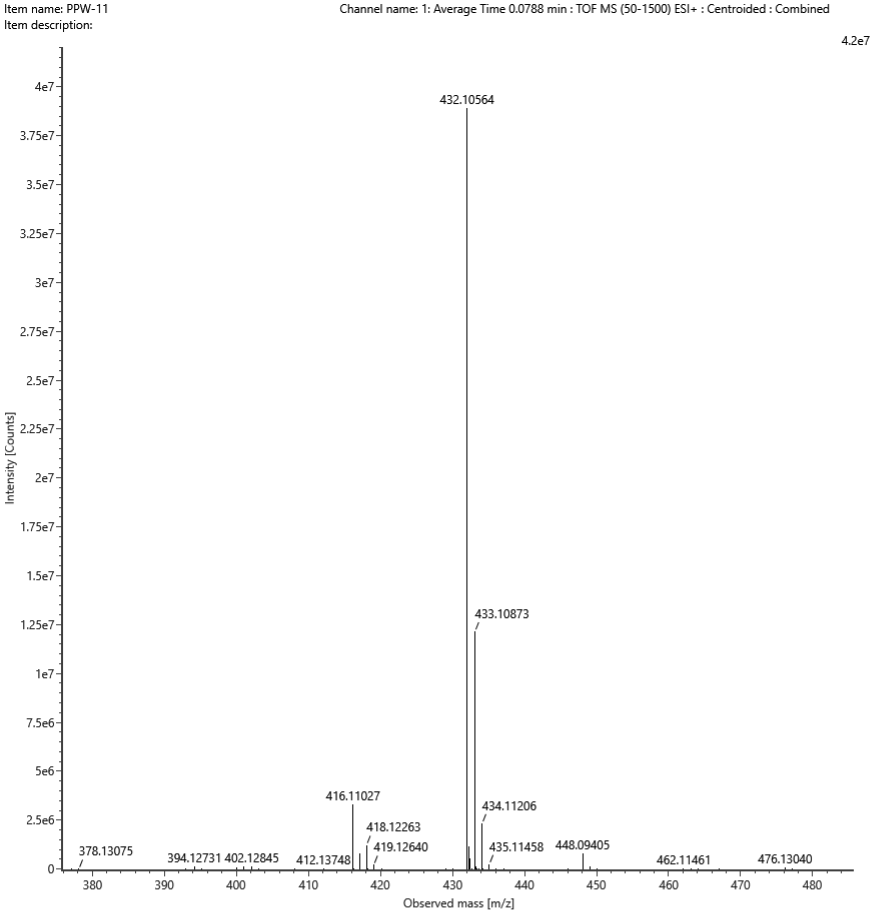


**Figure S21**. HRMS spectrum of compound **4g**


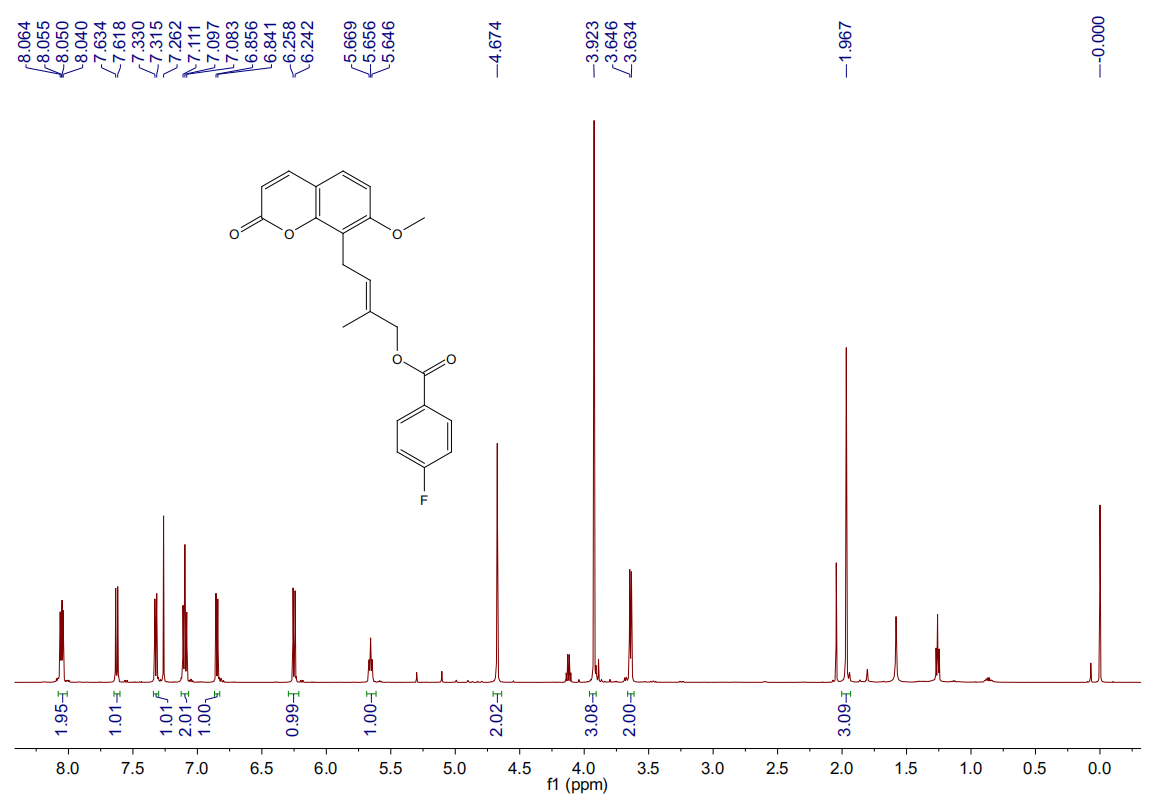


**Figure S22**. ^1^H NMR spectrum of compound **4h**


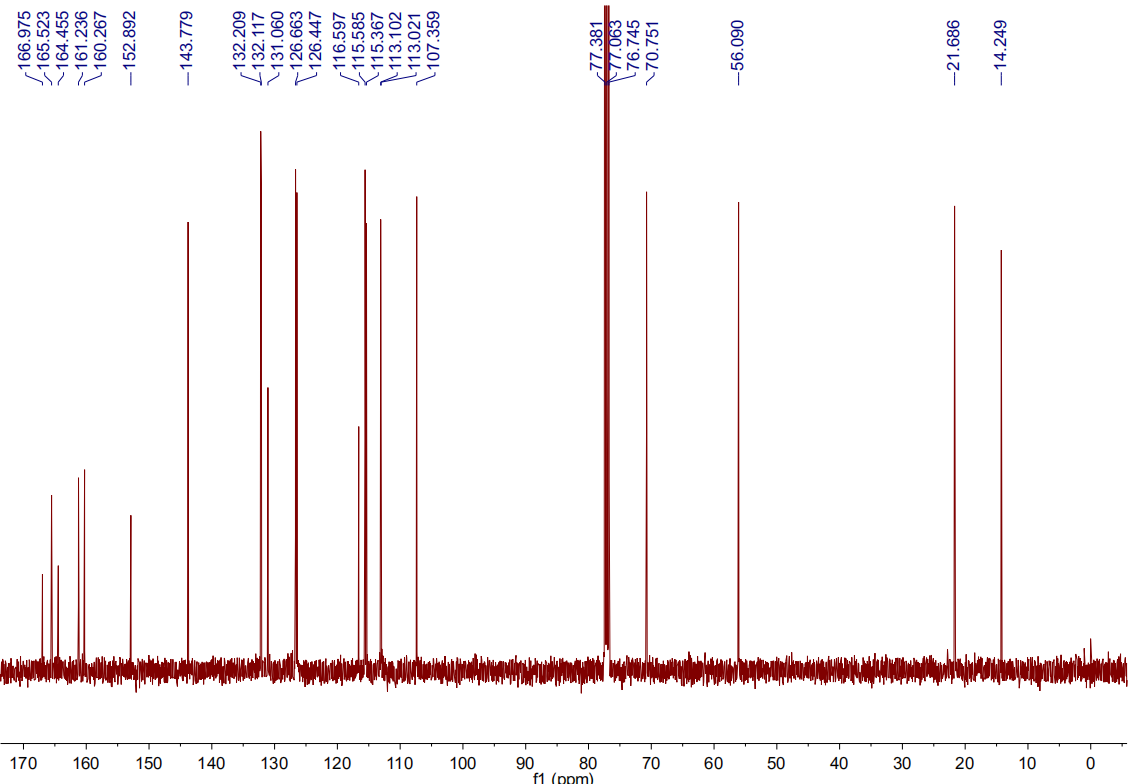


**Figure S23**. ^13^C NMR spectrum of compound **4h**


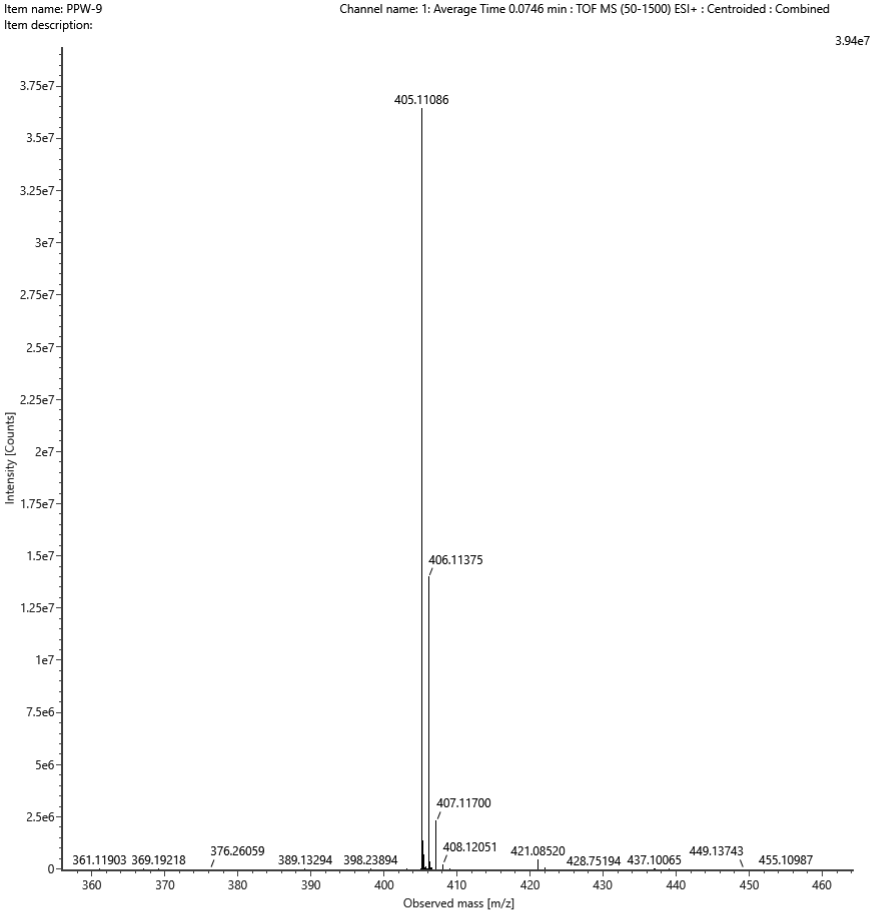


**Figure S24**. HRMS spectrum of compound **4h**


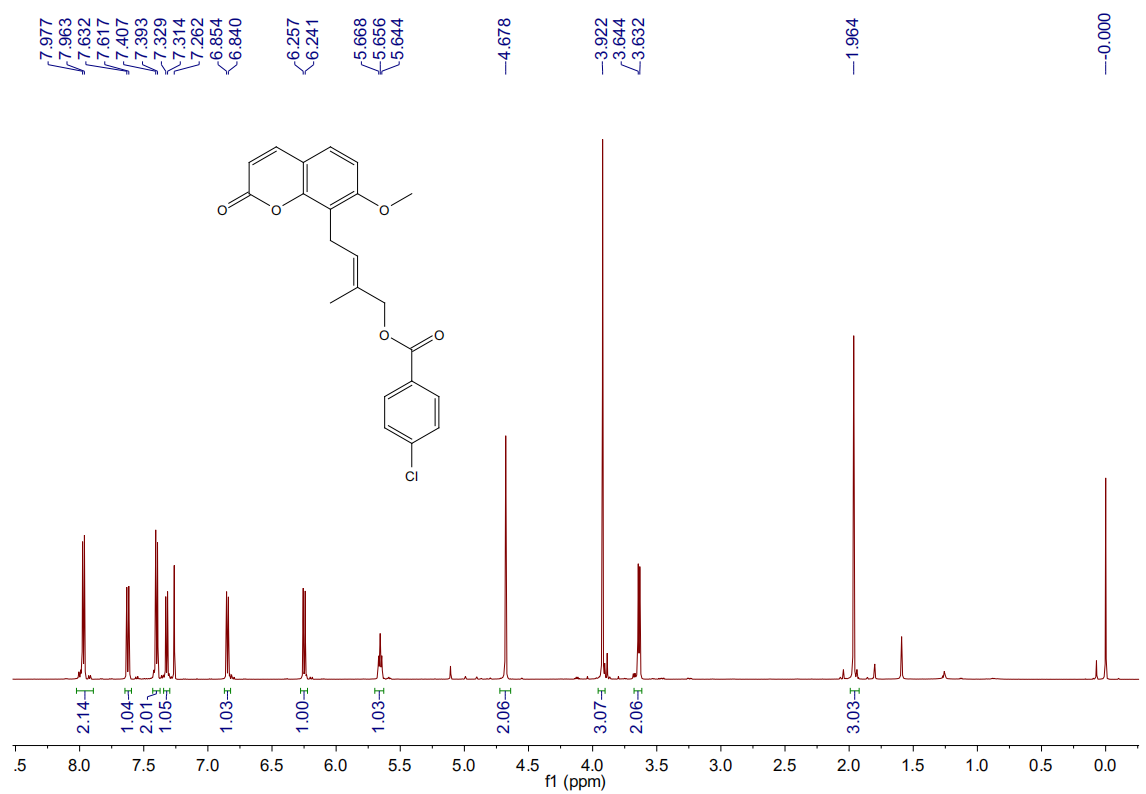


**Figure S25**. ^1^H NMR spectrum of compound **4i**


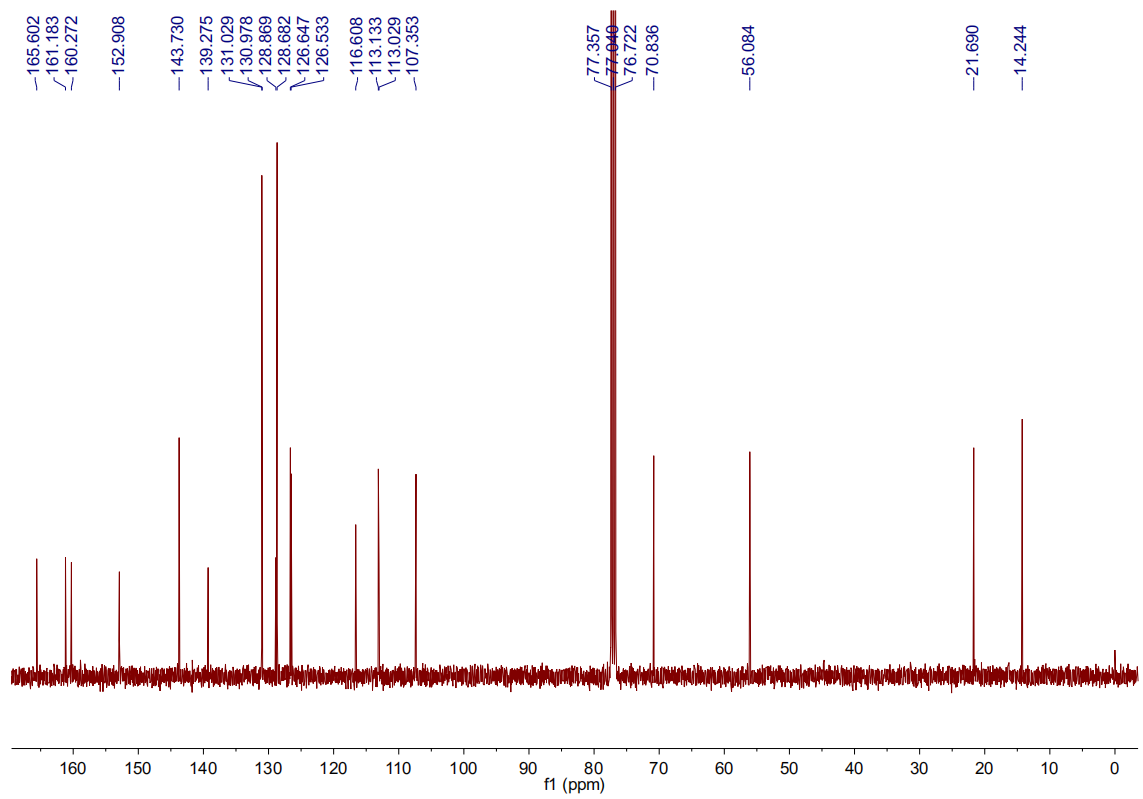


**Figure S26**. ^13^C NMR spectrum of compound **4i**


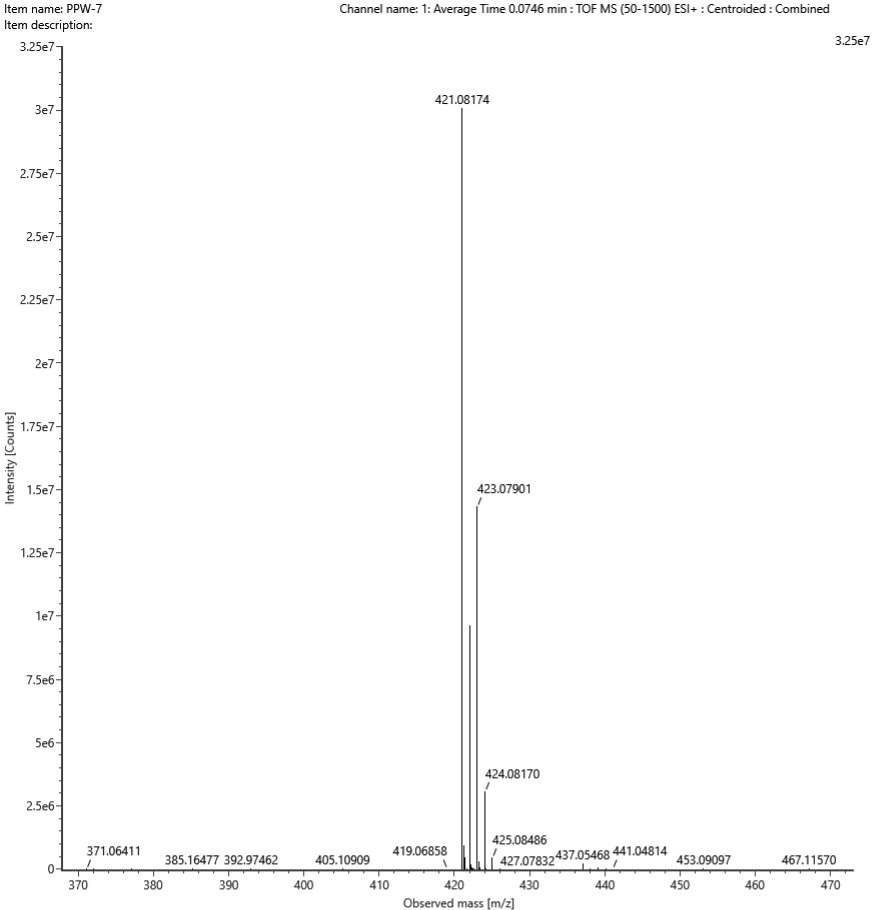


**Figure S27**. HRMS spectrum of compound **4i**


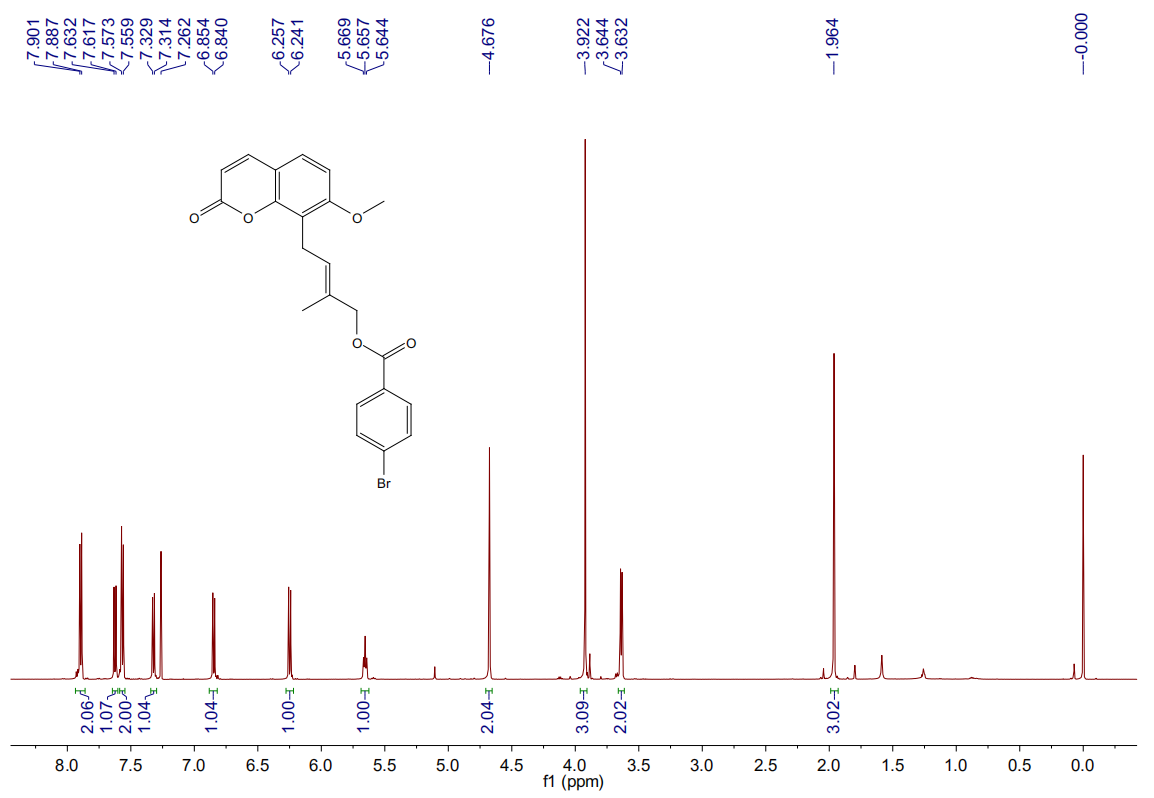


**Figure S28**. ^1^H NMR spectrum of compound **4j**


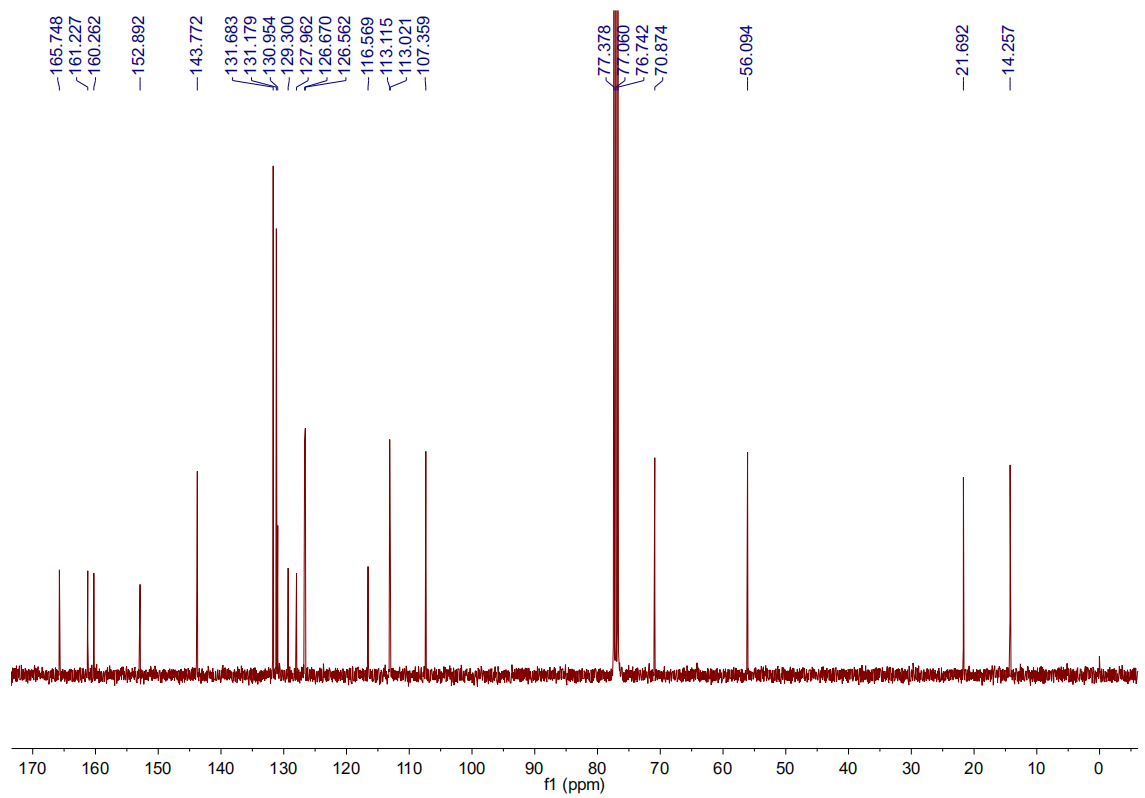


**Figure S29**. ^13^C NMR spectrum of compound **4j**


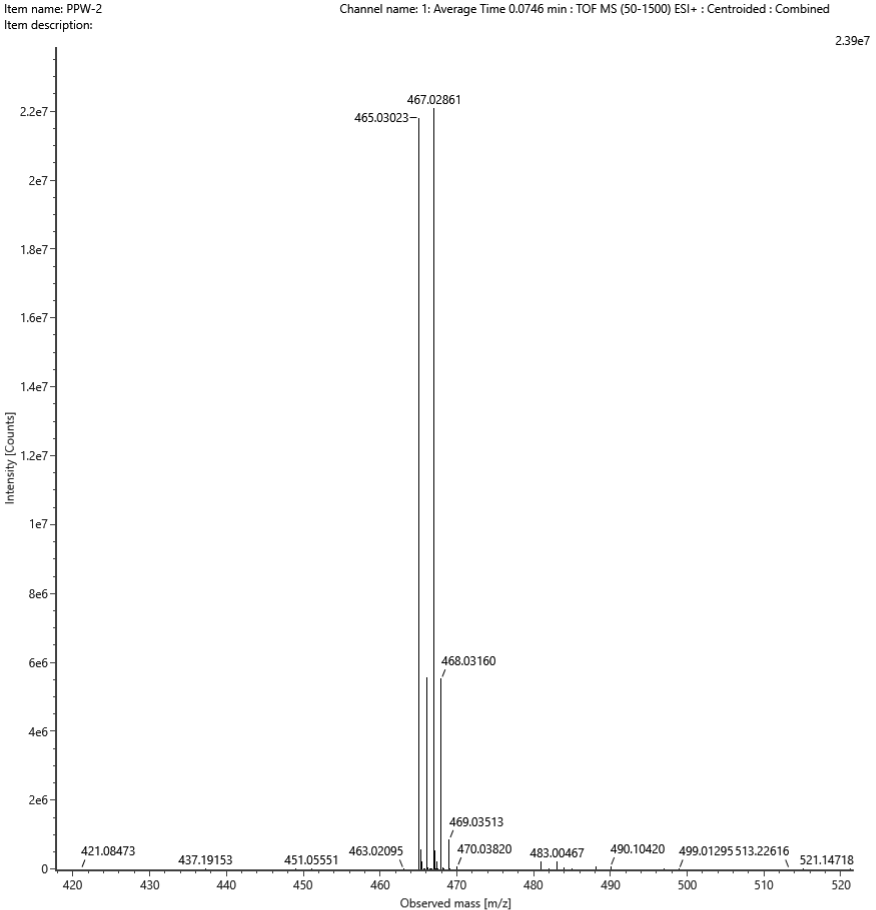


**Figure S30**. HRMS spectrum of compound **4j**


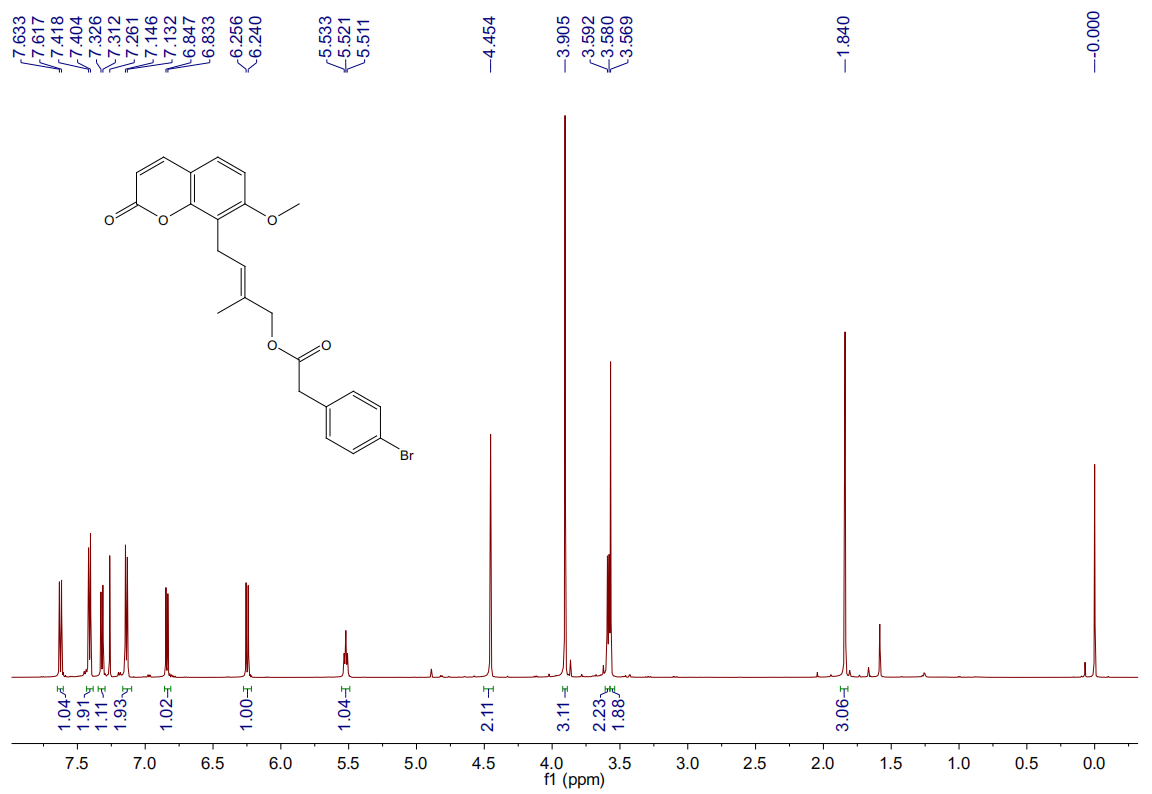


**Figure S31**. ^1^H NMR spectrum of compound **4k**


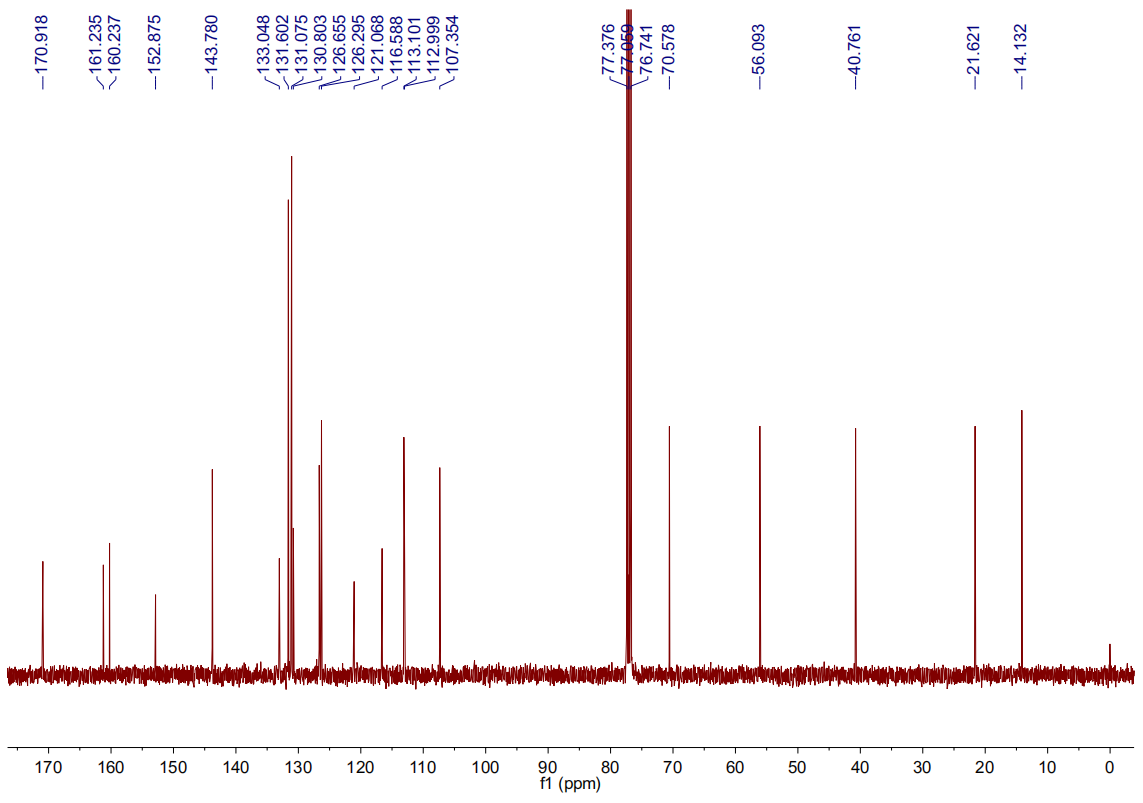


**Figure S32**. ^13^C NMR spectrum of compound **4k**


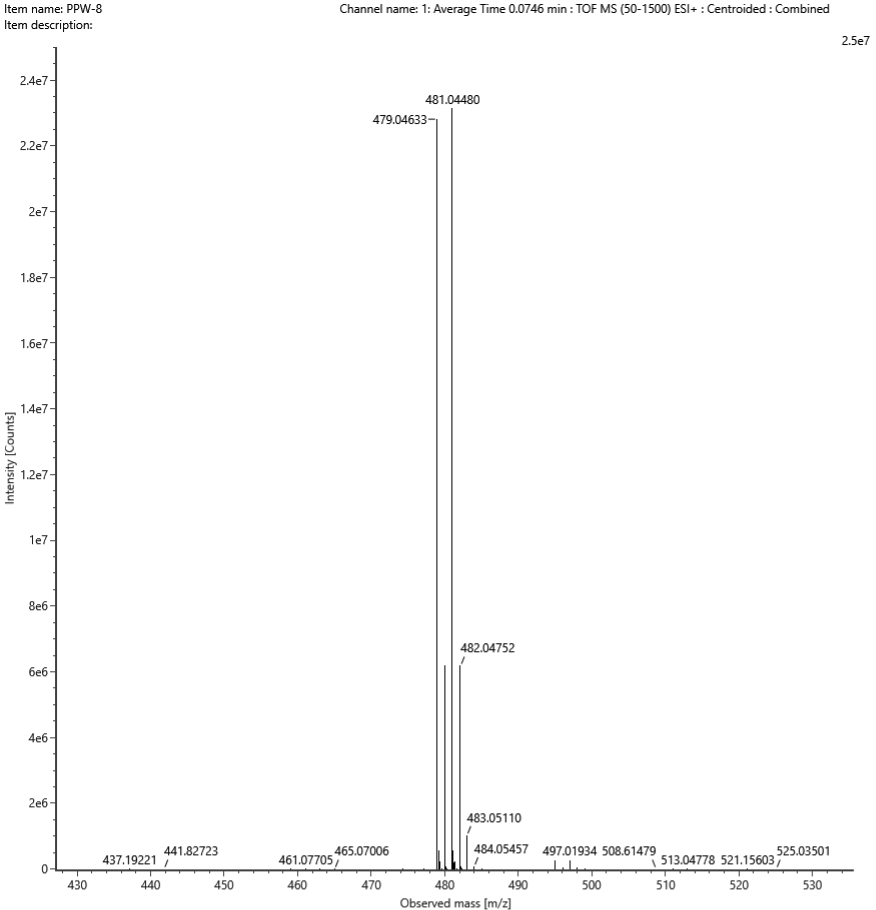


**Figure S33**. HRMS spectrum of compound **4k**


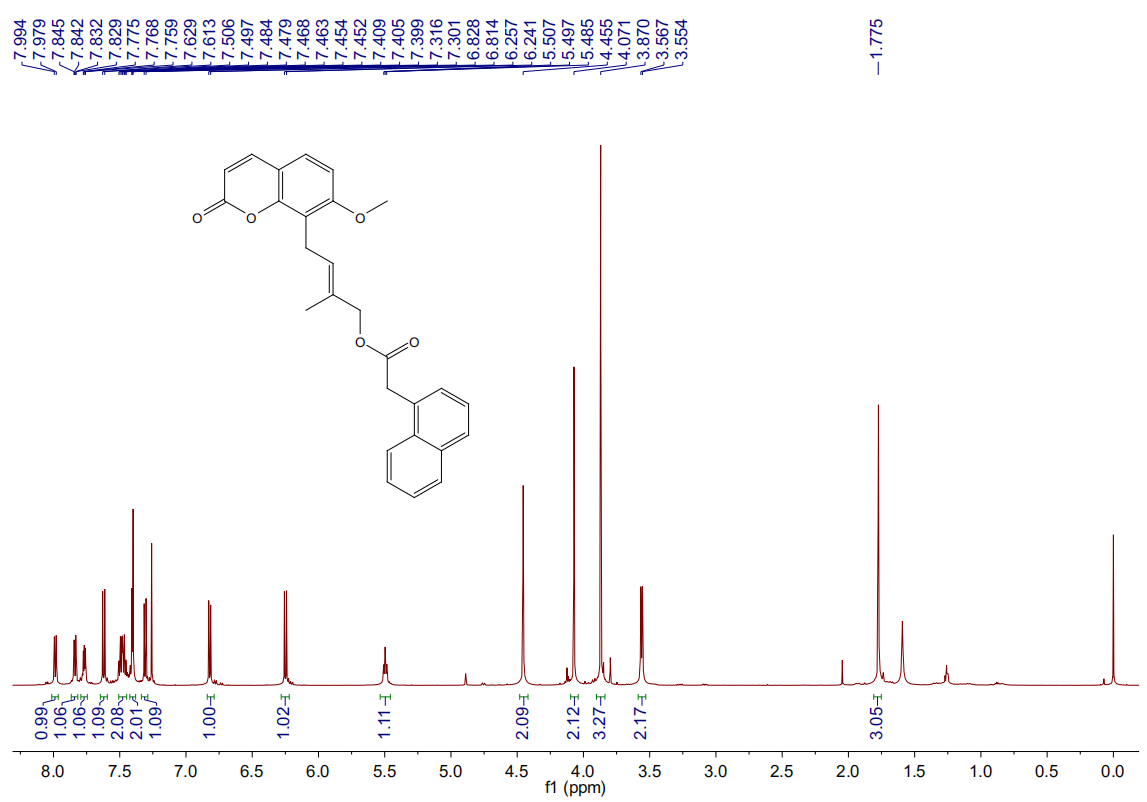


**Figure S34**. ^1^H NMR spectrum of compound **4l**


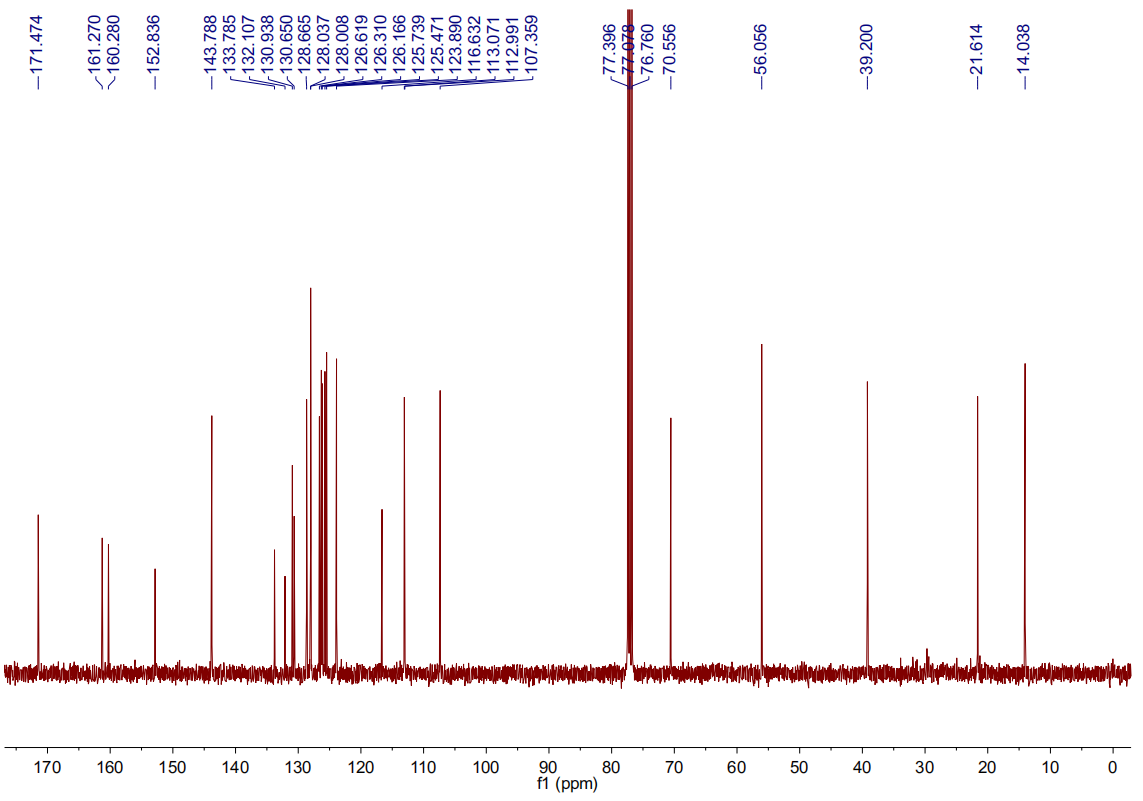


**Figure S35**. ^13^C NMR spectrum of compound **4l**


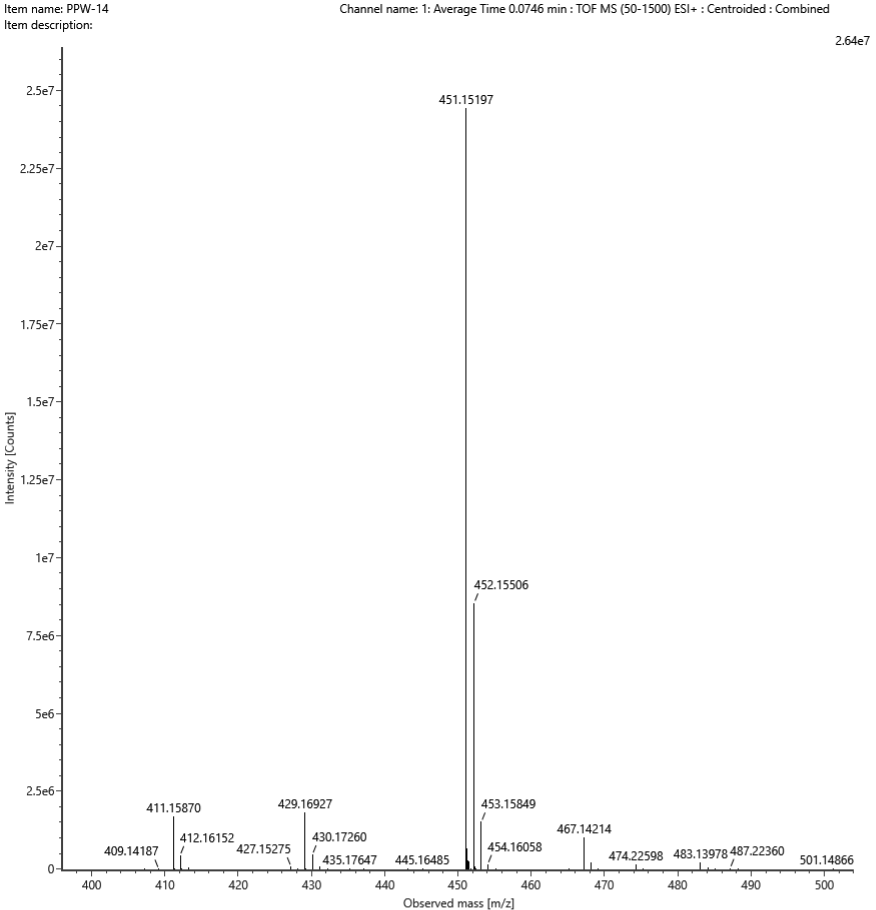


**Figure S36**. HRMS spectrum of compound **4l**


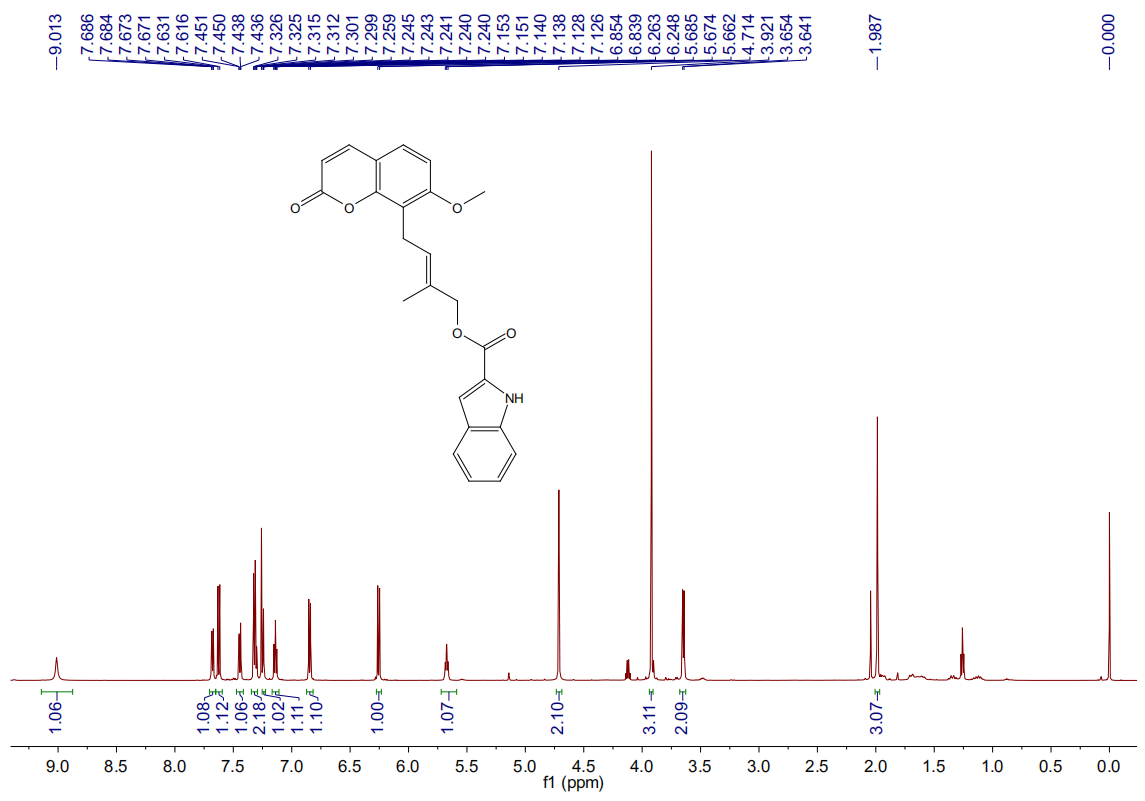


**Figure S37**. ^1^H NMR spectrum of compound **4m**


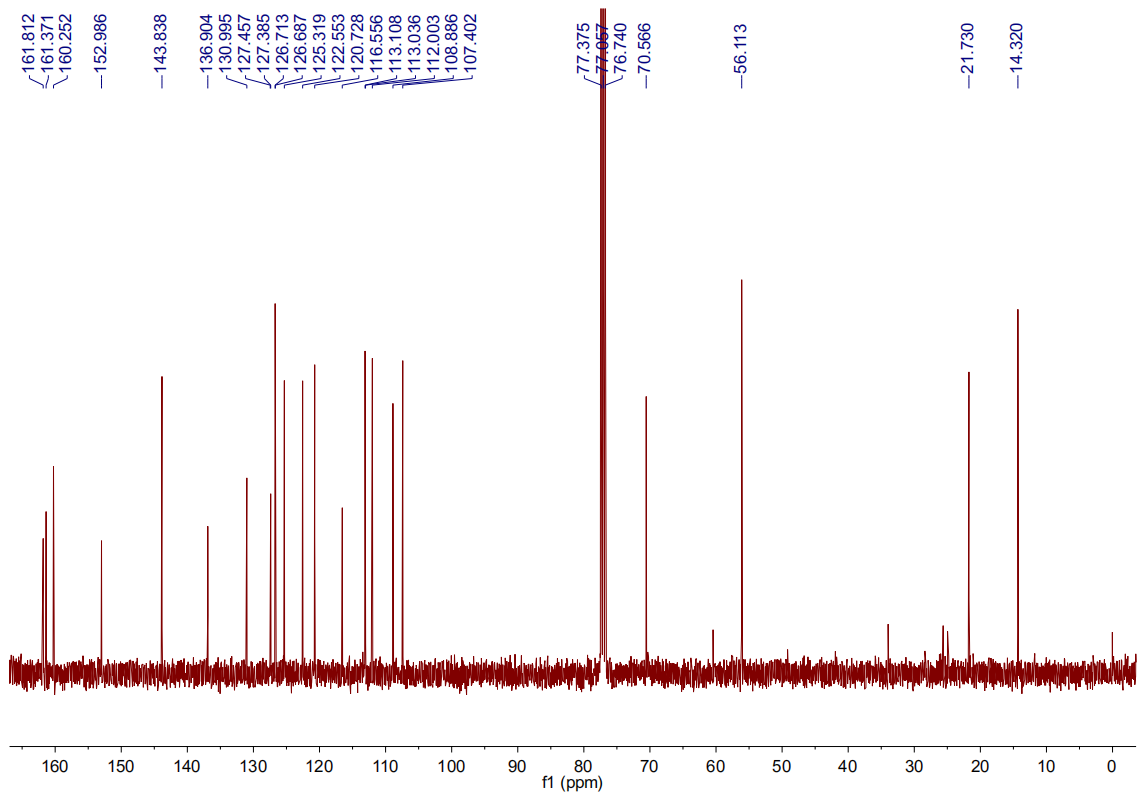


**Figure S38**. ^13^C NMR spectrum of compound **4m**


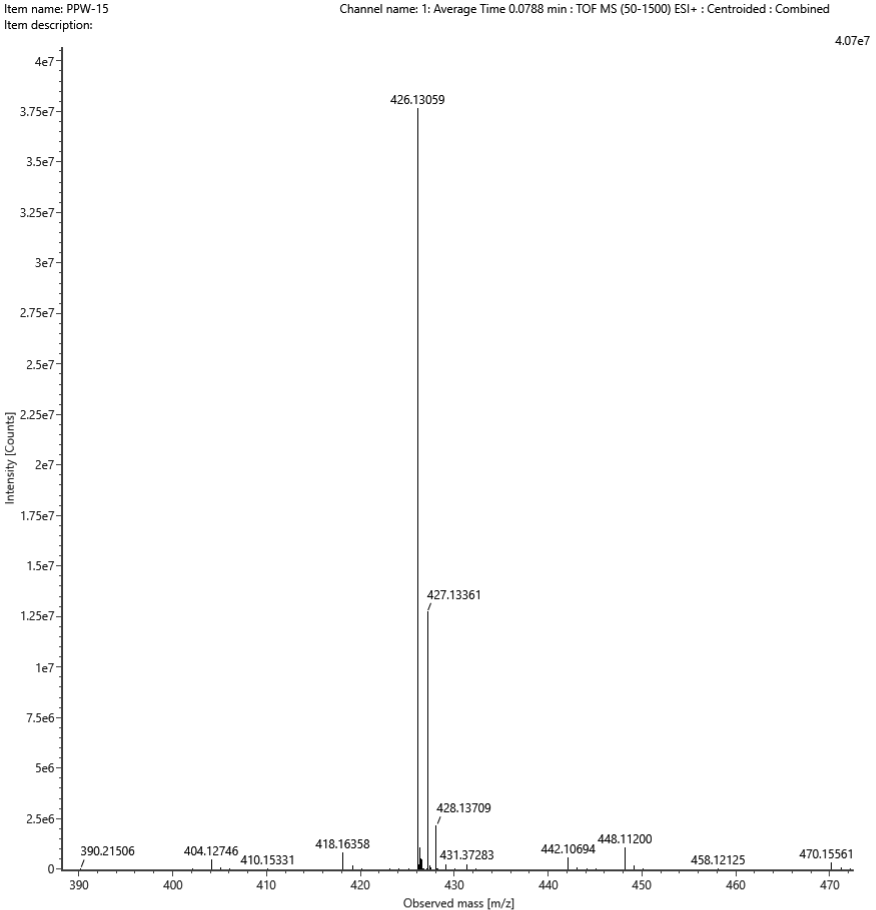


**Figure S39**. HRMS spectrum of compound **4m**


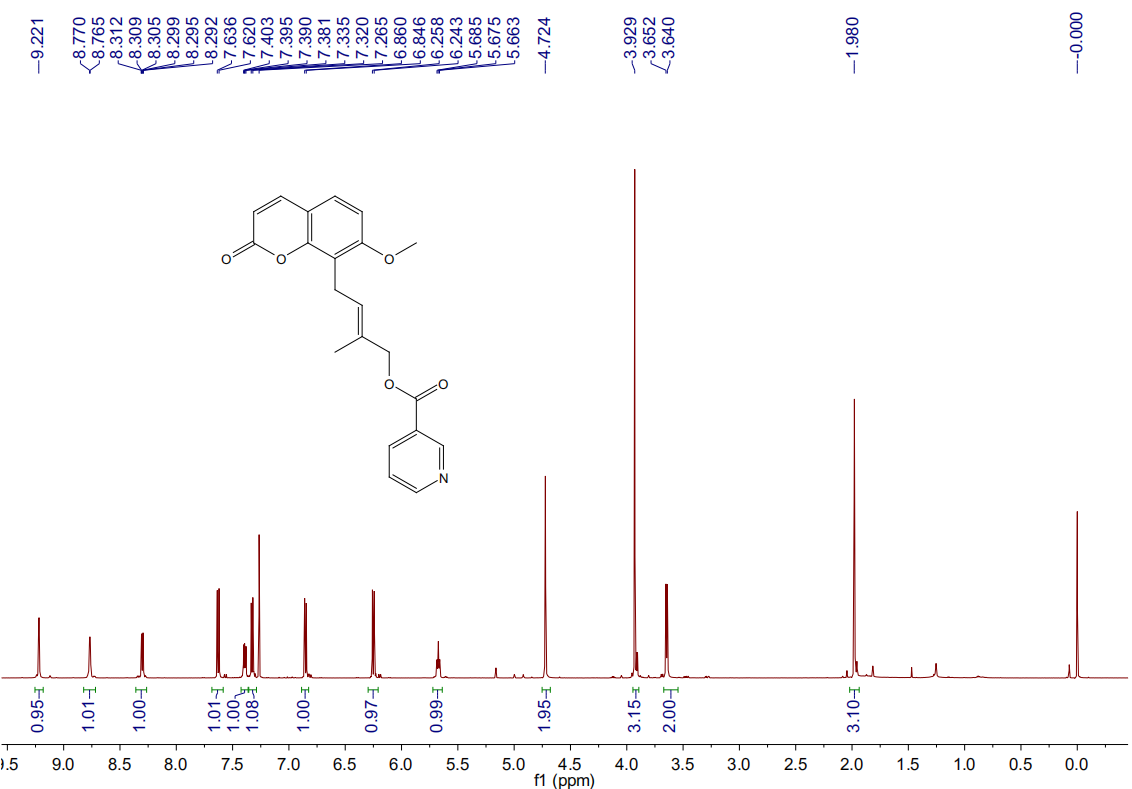


**Figure S40**. ^1^H NMR spectrum of compound **4n**


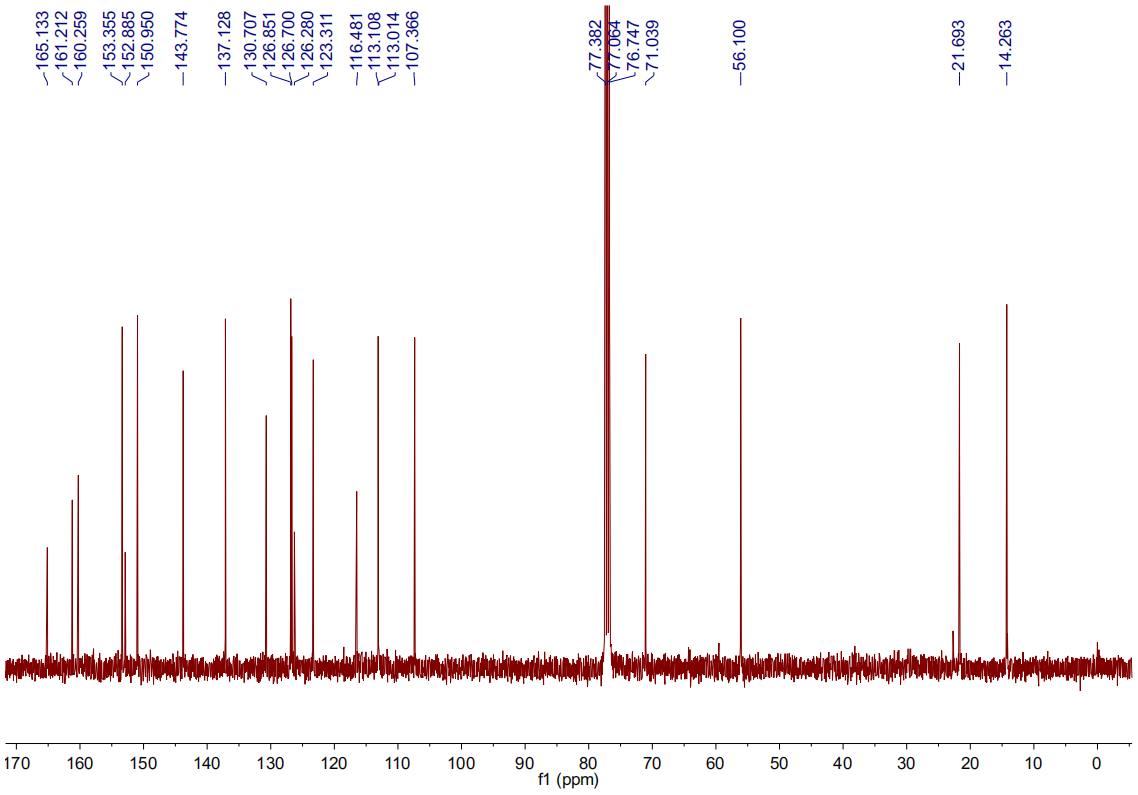


**Figure S41**. ^13^C NMR spectrum of compound **4n**


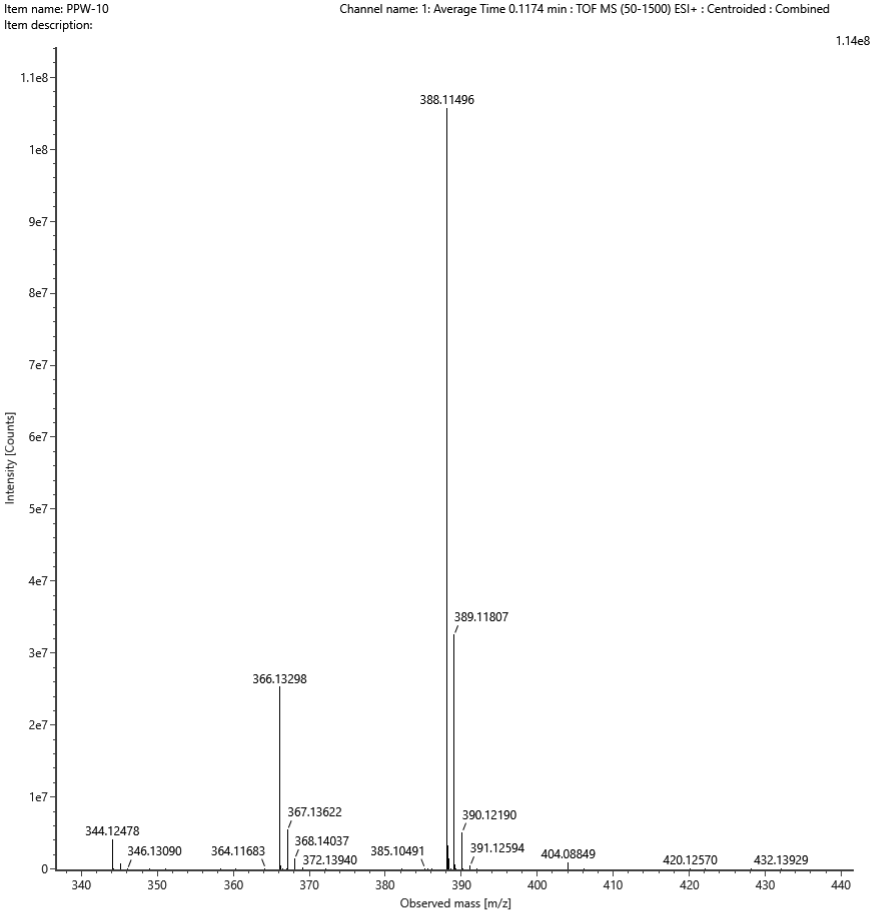


**Figure S42**. HRMS spectrum of compound **4n**


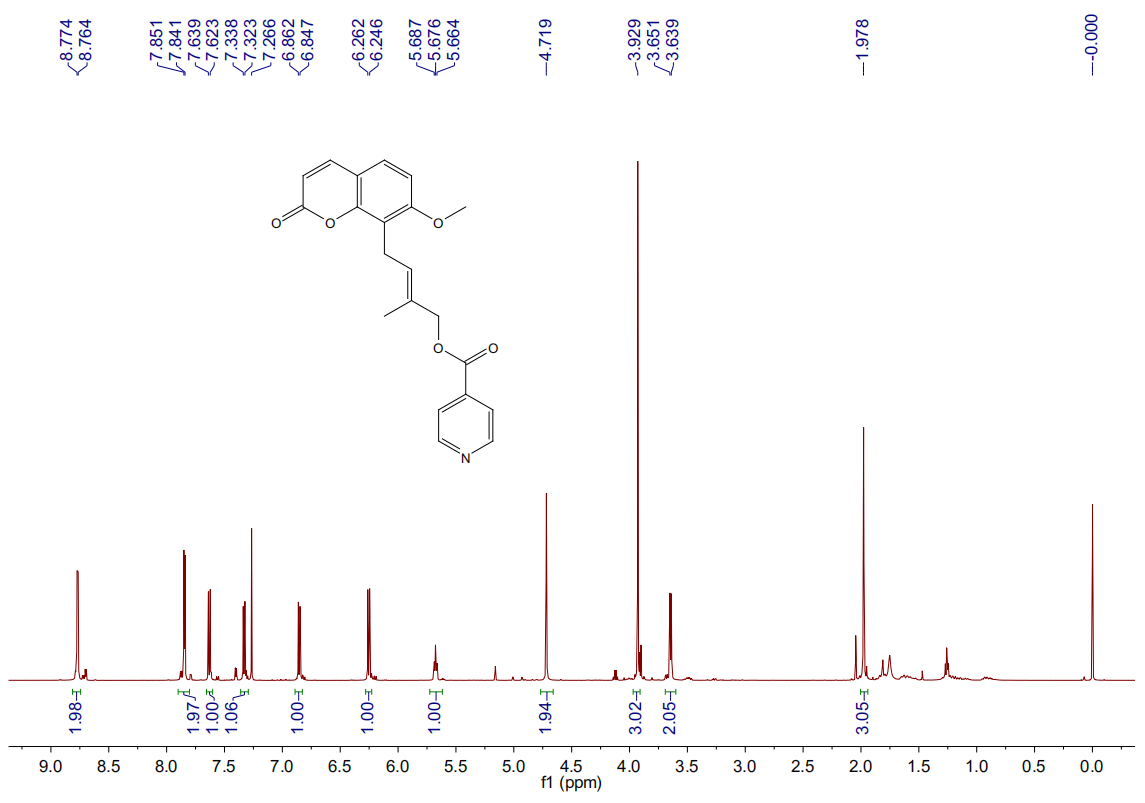


**Figure S43**. ^1^H NMR spectrum of compound **4o**


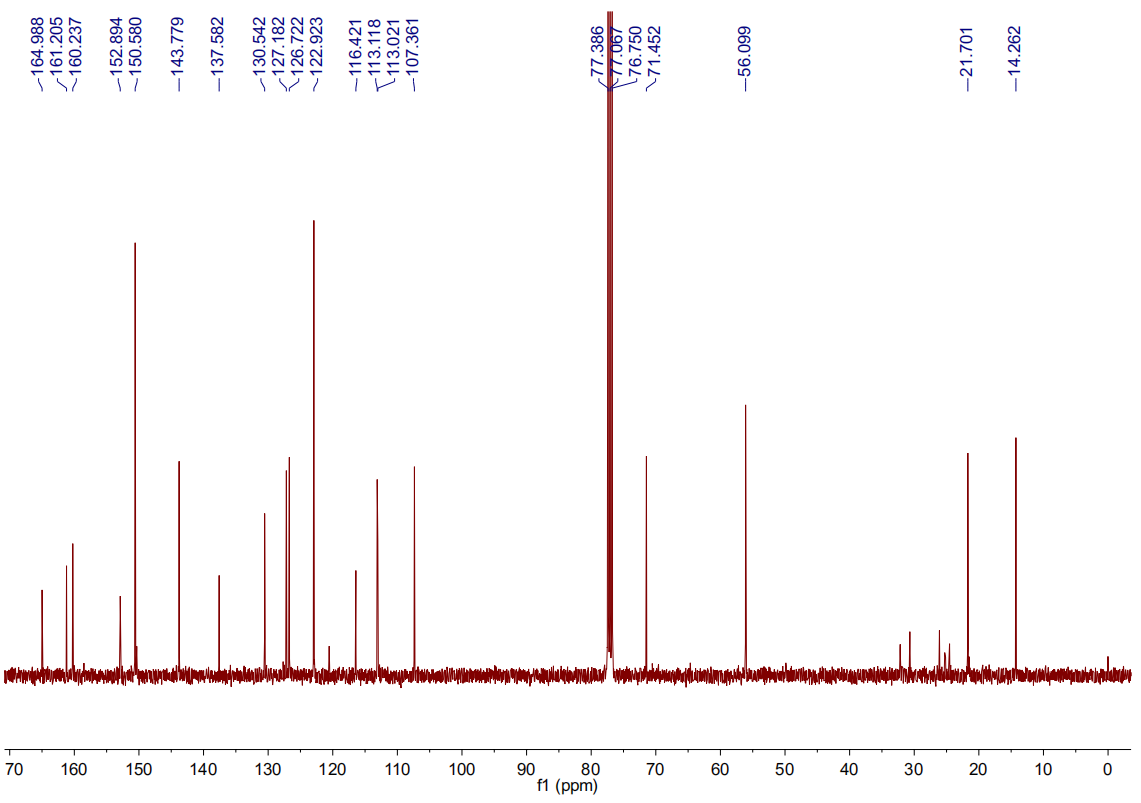


**Figure S44**. ^13^C NMR spectrum of compound **4o**


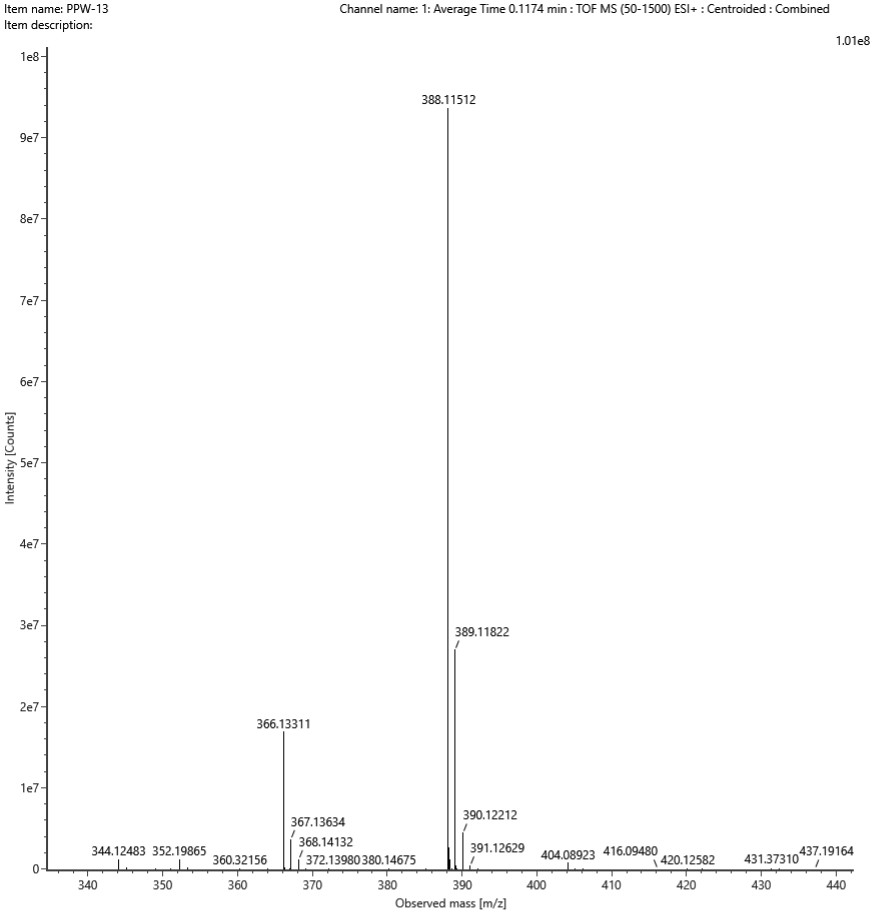


**Figure S45**. HRMS spectrum of compound **4o**


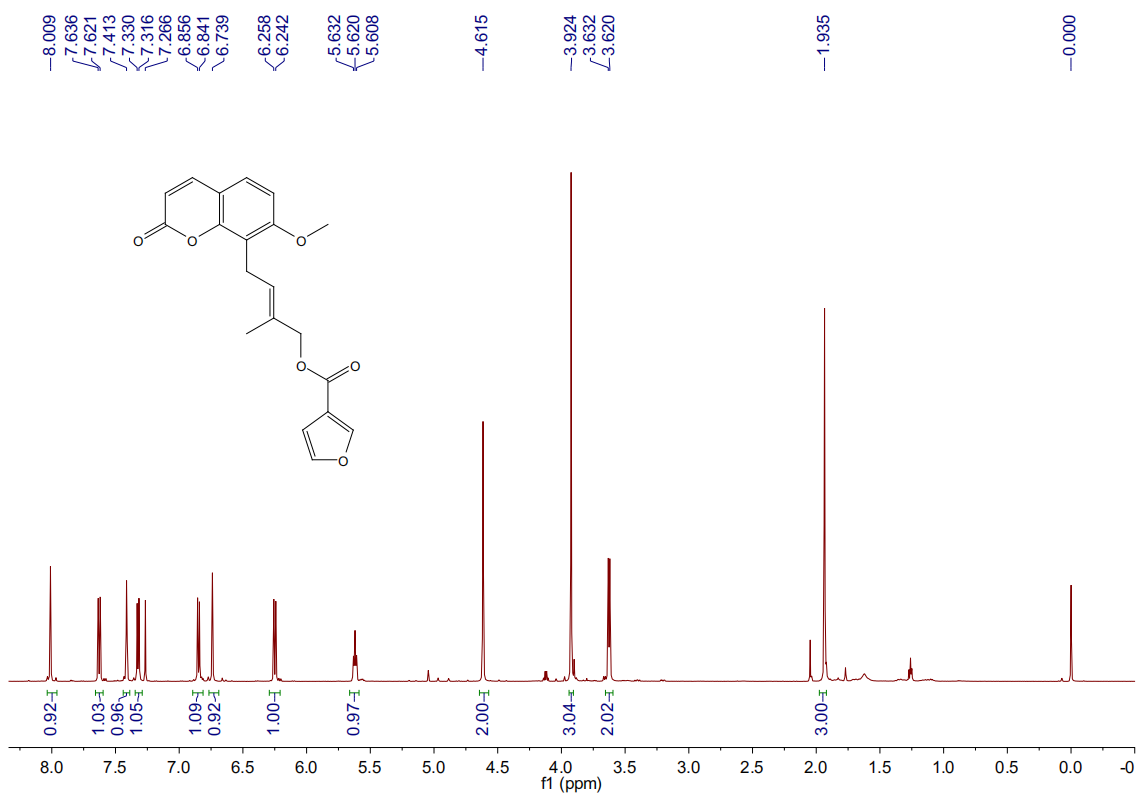


**Figure S46**. ^1^H NMR spectrum of compound **4p**


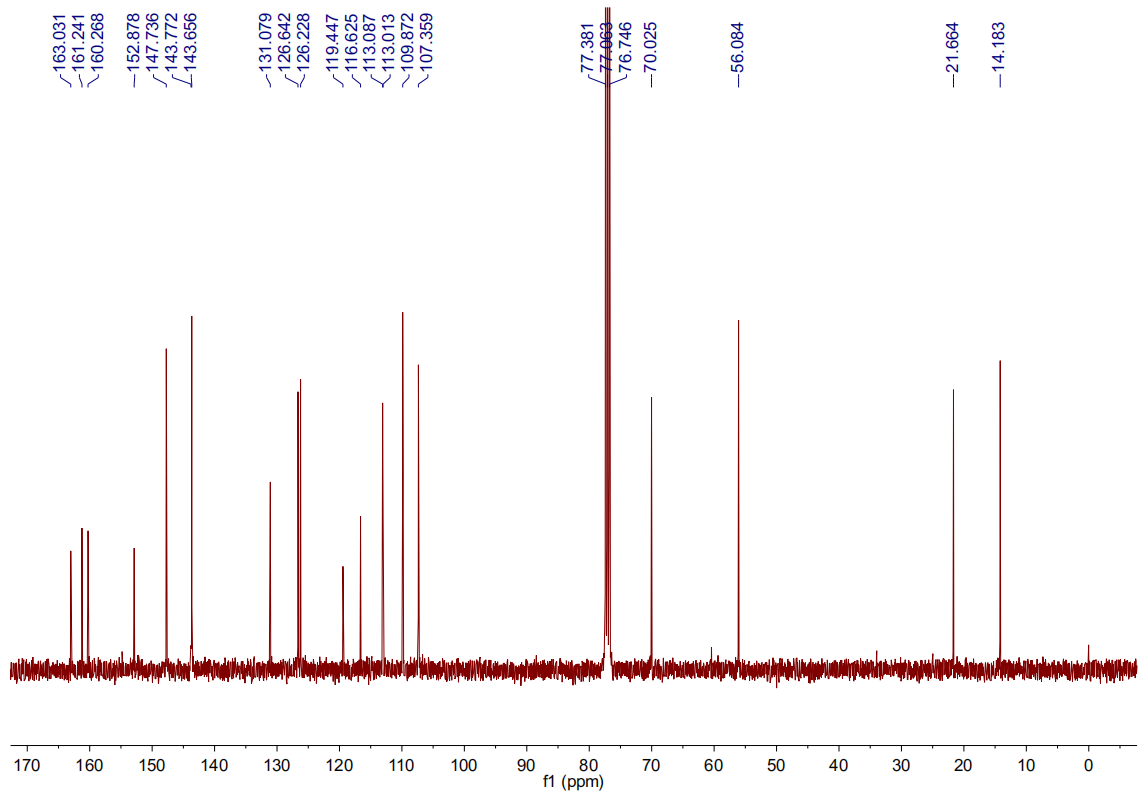


**Figure S47**. ^13^C NMR spectrum of compound **4p**


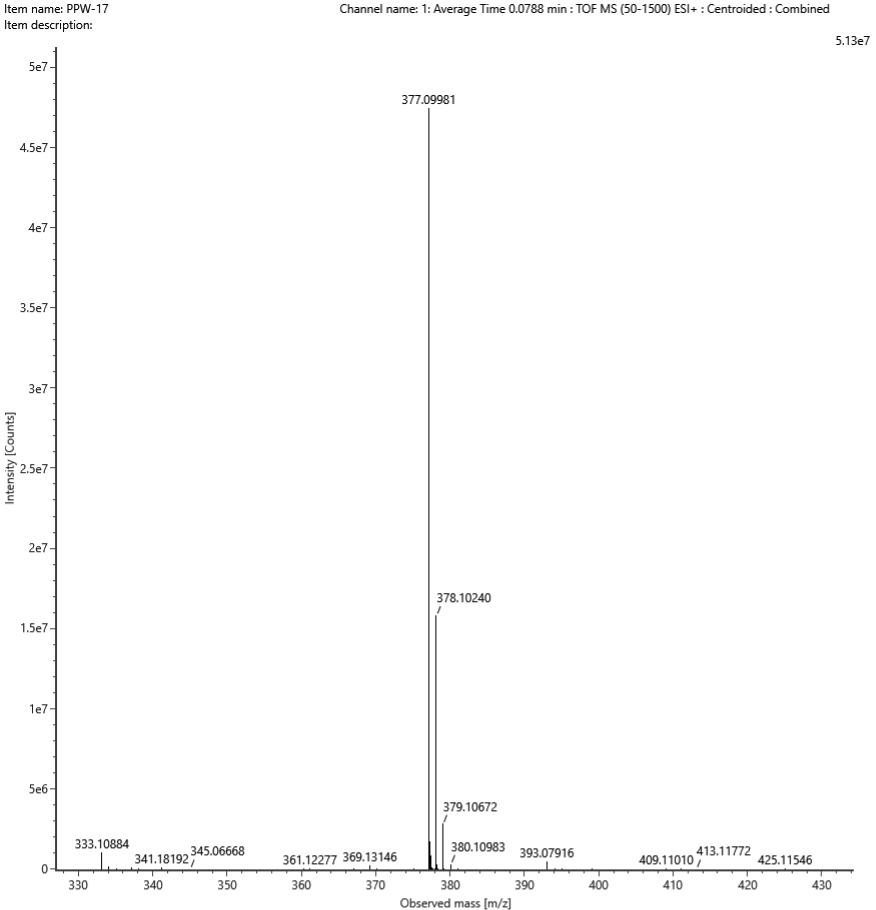


**Figure S48**. HRMS spectrum of compound **4p**


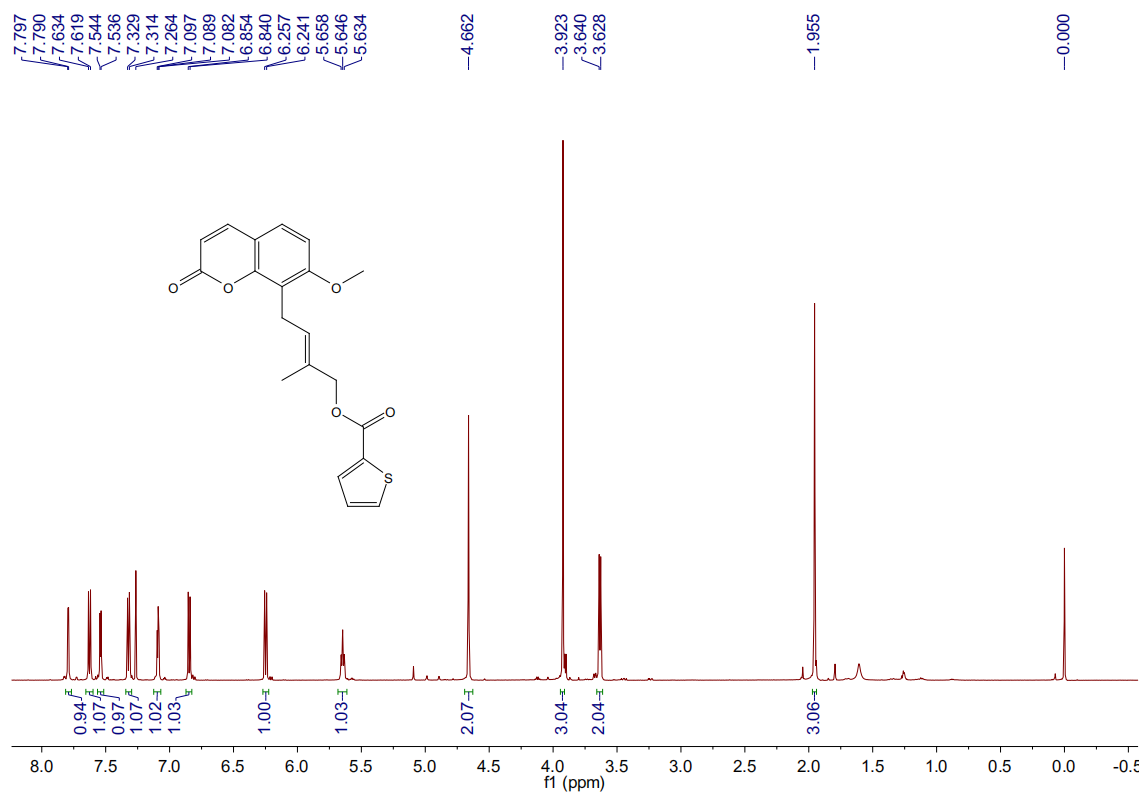


**Figure S49**. ^1^H NMR spectrum of compound **4q**


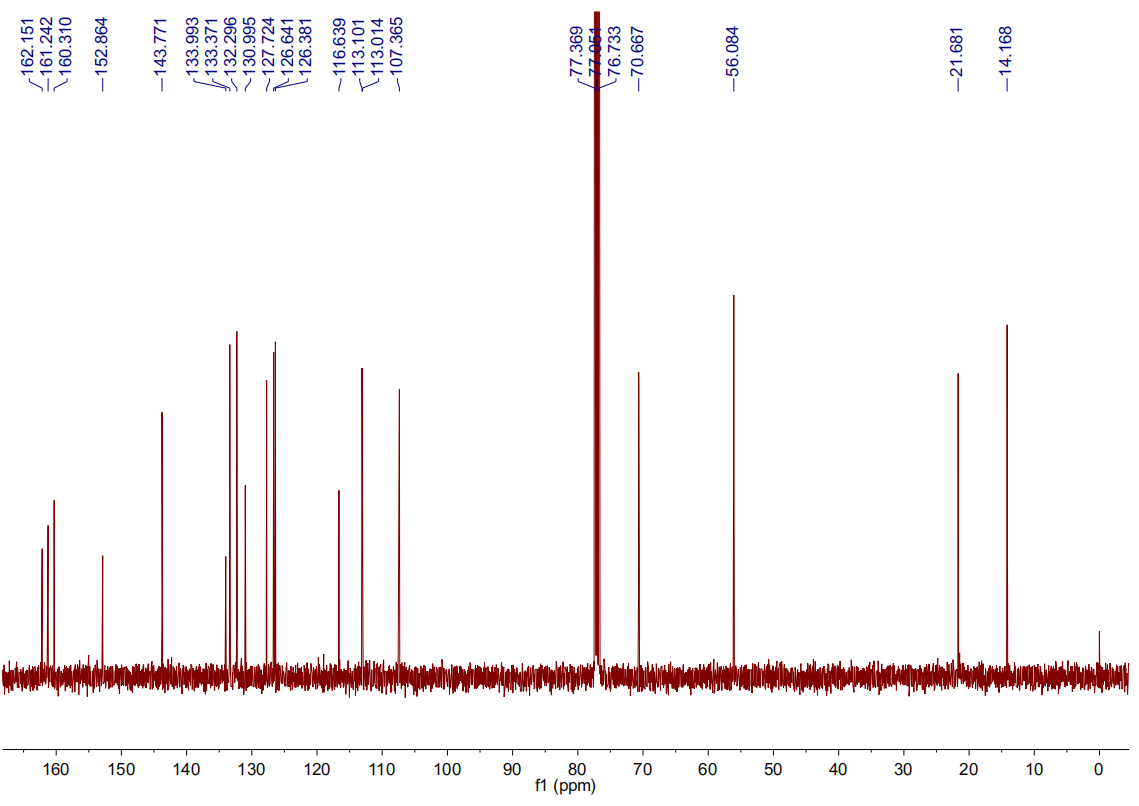


**Figure S50**. ^13^C NMR spectrum of compound **4q**


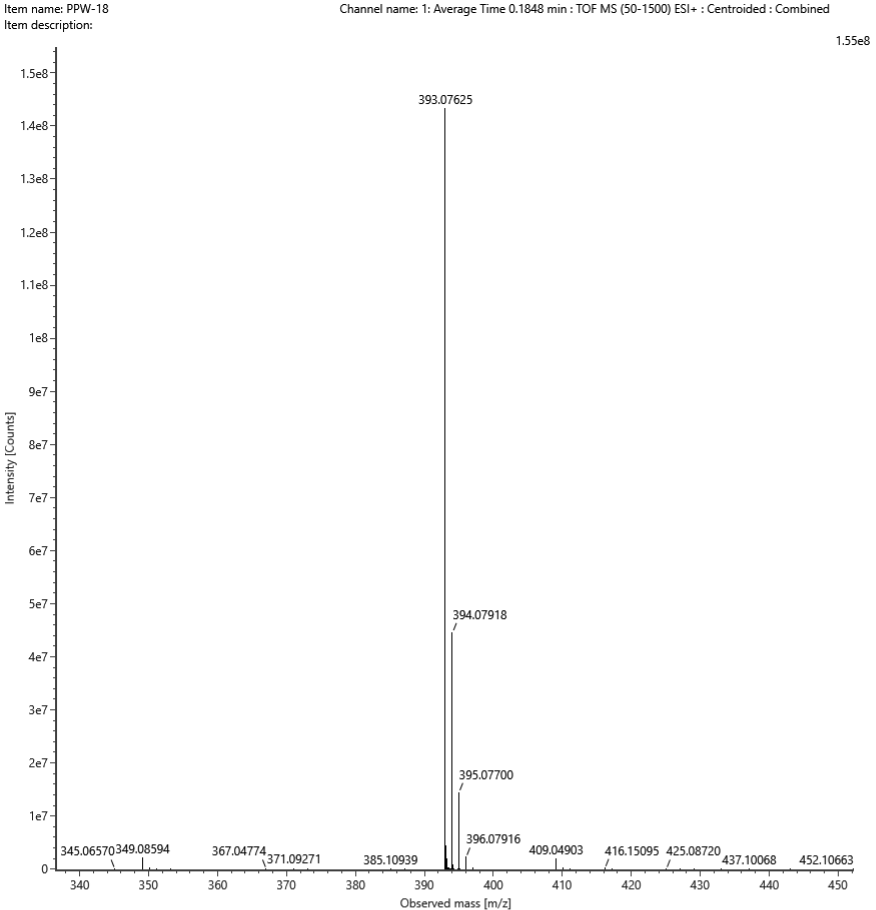


**Figure S51**. HRMS spectrum of compound **4q**


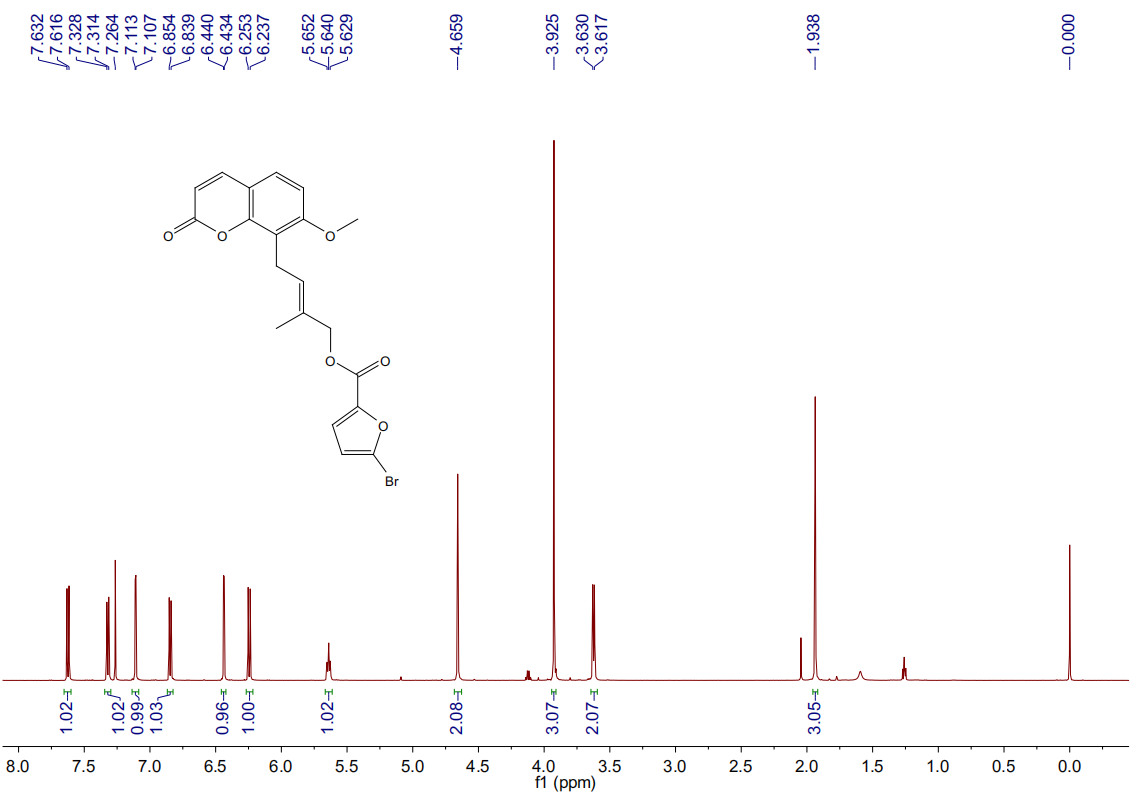


**Figure S52**. ^1^H NMR spectrum of compound **4r**


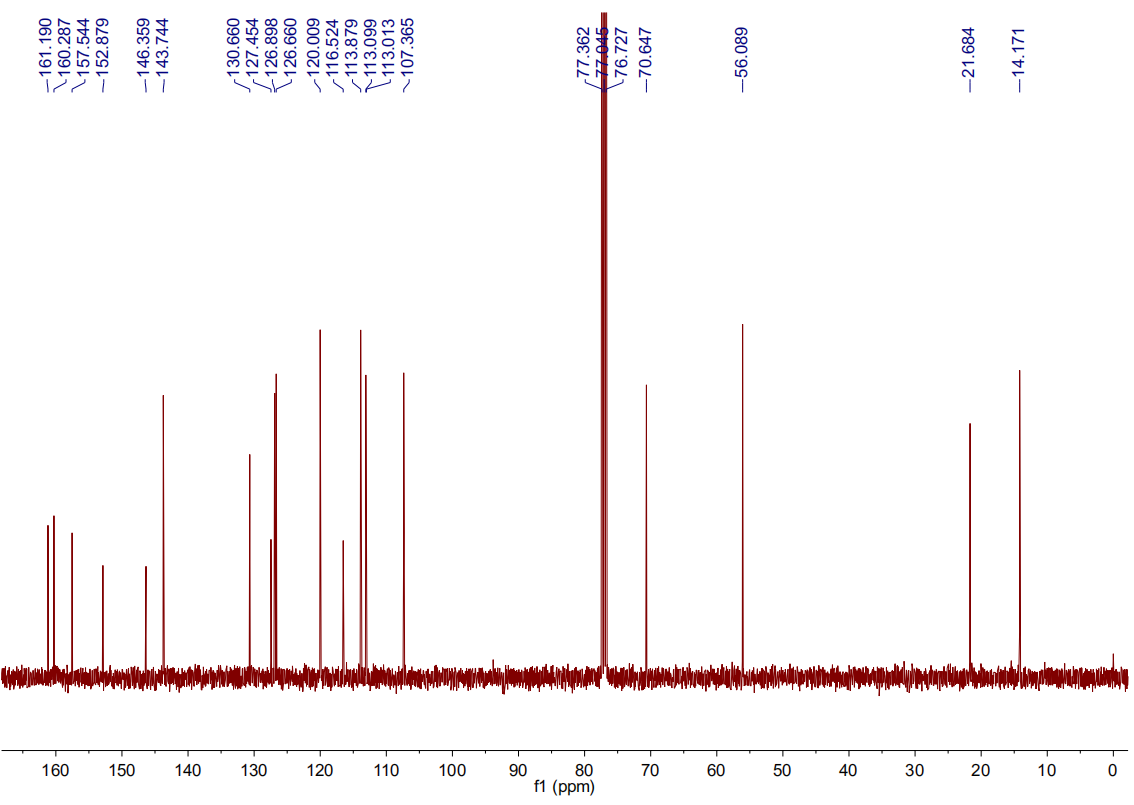


**Figure S53**. ^13^C NMR spectrum of compound **4r**


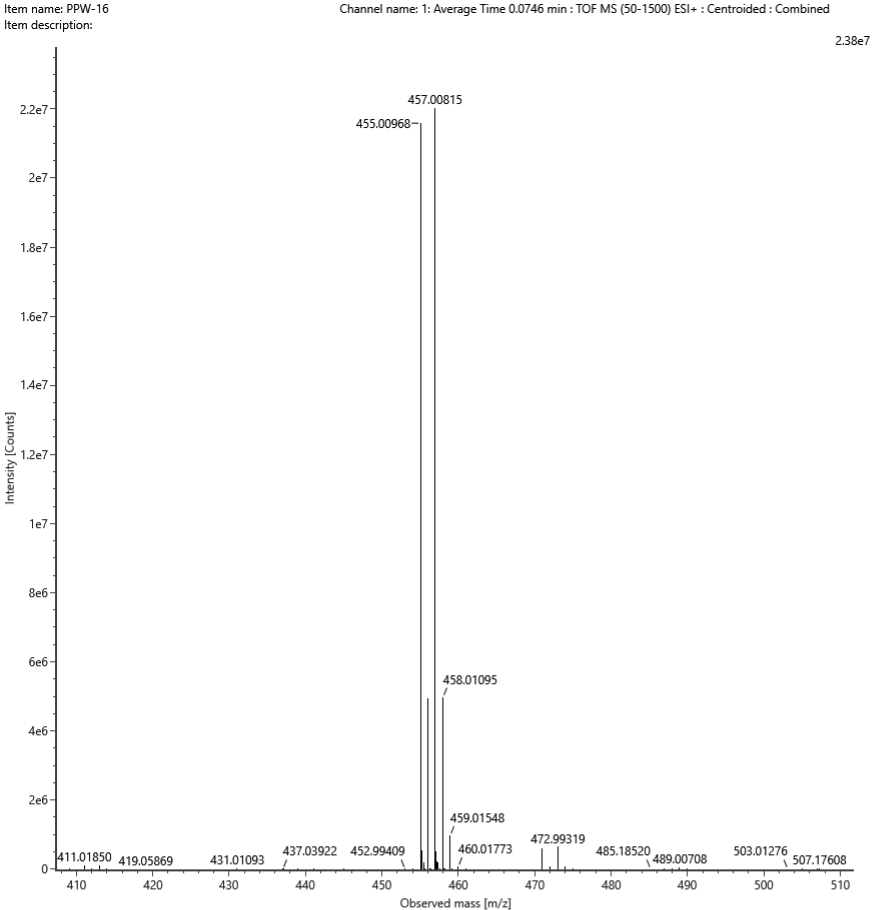


**Figure S54**. HRMS spectrum of compound **4r**
